# Analysis of the peristaltic flow of a variable viscosity Carreau fluid affected by temperature and concentration through an endoscope hollow flexible channel

**DOI:** 10.12688/f1000research.172584.1

**Published:** 2026-01-07

**Authors:** Salwa k. kazem Al-Tamimi, Dheia G. Salih Al-Khafajy

**Affiliations:** 1 Department of Fluid Mechanics, University of Al-Qadisiya, AL-Qadisiya, AL-Qadisiya, 58001, Iraq; 2 Department of Fluid Mechanics, University of Al-Qadisiya, Al-Qadisiya, Al-Qadisiya, 58001, Iraq

**Keywords:** Viscous Carreau fluid, peristaltic flow, endoscopic hollow flexible channel.

## Abstract

**Background:**

Peristaltic or undulating flow plays a significant role in various biomedical and industrial processes, where it provides an efficient mechanism for transporting fluids through flexible conduits such as catheters and endoscopic channels. Understanding such flow behavior is essential for improving medical devices and industrial applications involving non-Newtonian fluids.

**Methods:**

This study investigates the peristaltic motion of a Carreau fluid whose viscosity varies with both temperature and concentration within a flexible, axisymmetric channel composed of two overlapping cylindrical tubes. The outer wall of the channel exhibits a sinusoidal wave pattern, simulating a realistic endoscopic configuration. The governing nonlinear, nonhomogeneous partial differential equations were formulated in cylindrical coordinates under the assumption of a long wavelength and low Reynolds number. The equations were transformed into a dimensionless form and solved using the uniform perturbation method. Graphical analyses were performed using Mathematica software.

**Results:**

The results illustrate the combined effects of temperature-dependent and concentration-dependent viscosity on the velocity distribution and pressure gradient within the channel. Increasing temperature and solute concentration were found to enhance fluid velocity and reduce flow resistance.

**Conclusions:**

The study provides a comprehensive understanding of peristaltic transport in variable-viscosity Carreau fluids under realistic physiological conditions. These findings may contribute to optimizing the design and performance of endoscopic and biomedical fluid transport systems.

## 1. Introduction

An unique and crucial mechanism for carrying fluids via flexible tubes, undulating flow has several medicinal and industrial uses. The non-Newtonian fluid model that describes the behavior of increasing viscosity, the Carreau model, is the main focus of this work. Ali and Hayat compared the results for Newtonian and Carreau fluids, and they looked at the pumping characteristics, axial pressure gradient, and trapping mechanisms. ^7^ Using the long wavelength and low Reynolds number assumption, Nadeem and colleagues studied the propagation of peristaltic waves in Carreau fluid down the horizontal side walls of a rectangular duct. ^10^ Ullah et al. ^12^ investigated Carreau fluid peristaltic flow in an elastic tube. Peristaltic flow of Jeffrey fluid inside a flexible tube was studied by Al-Khalidi and Al-Khafajy. ^6^

The most important and consequential property of fluid motion is viscosity. The medical and food industries rely heavily on viscosity, and a number of mathematical models explain how temperature and fluid concentration affect its flow via different channels. Although fluid velocity shows minimal modification with changes in concentration and position within the channel, most research agrees that raising the temperature boosts it. ^1, 11, 5, 9^ Nadeem et al. ^8^ investigated the peristaltic flow of a reactive viscous fluid with viscosity that depends on temperature, while Akram and Akbar ^2^ performed a biological study of Careau nanofluid within an endoscope with changing viscosity. The impact of concentration and temperature on oscillatory flow in an inclined porous channel was investigated by Al-Khafajy and Labban, ^4^ while the impact of concentration and temperature on the peristaltic flow of a Williamson fluid through an endoscopic hollow flexible channel was studied by Al-Delfi and Al-Khafajy. ^3^

Previous studies inspired us to study a mathematical model of the flow of a non-Newtonian, incompressible, and variable-viscosity fluid, which is the Carreau fluid (similar to human blood), through a flexible wave channel with a catheter tube in the middle. This fluid is influenced by changes in temperature and concentration at the channel wall.

## 2. Mathematical formulation

We study the peristaltic flow of an incompressible Carreau fluid between two cylinders that are in a central location, with an endoscope in the middle of the main channel that has a flexible wall structured like a sine wave. A cylinder's coordinates are specified by the radius of the channel (R) and the tube's axis (Z).

The geometry wall of the flow channel form is

$$\begin{array}{l} \bar{r}=\bar{r_{1}}\left(\bar{z},\bar{t}\right)=a_{1}\\ \bar{r}=\bar{r_{2}}\left(\bar{z},\bar{t}\right)=a_{2}+b\text{sin}\left(\frac{2\pi }{\gamma }\left(\bar{z}-c\bar{t}\right)\right)\; \end{array}$$

Here “the unobstructed radius of the pipe” is represented by $\;a_{1}\;$ , the radius of the disturbed tube is represented by $\;a_{2}$ , *b* is “amplitude of the peristaltic wave”, γ is “a wave length”, c is “a wave propagation speed”, and $\bar{t}\;$ is “a time”.

The basic governing equations of the problem system

$$\nabla .\bar{U}=0\;\left(\text{continuity equation}\right)$$ (1)

$$\rho \left(\bar{U.}\nabla \right)\bar{U}=\nabla \overset{´}{\sigma }+\mathrm{\rho g}\beta_{1}\left(T-T_{0}\right)+\mathrm{\rho g}\beta_{2}\left(∁-{∁}_{0}\right)\;\left(\text{momentum equation}\right)$$ (2)

$$T_{p}.\rho \left(\bar{U.}\nabla \right)T=K.\nabla^{2}T-\nabla .Q_{r}-Q\left(T-T_{0}\right)\;\left(\text{temperature equation}\right)$$ (3)

$$\left(\bar{U.}\bar{\nabla }\right)∁=D_{n}\nabla^{2}∁+\frac{D_{n}K_{T}}{T_{n}}\nabla^{2}T\;\left(\text{concentration equation}\right)$$ (4)

Where $\nabla^{2}=\frac{1}{r}\frac{\partial }{\partial r}\left(r\frac{\partial }{\partial r}\right)$ “Laplace operator”, $\bar{U}=\left({\bar{U}}_{1},0,{\bar{U}}_{3}\right)$ is “the velocity field”, ρ is a “density”, $\overset{´}{\;\sigma }$ is “the Cauchy stress tensor”, *T* is “the temperature”, ∁ is a concentration of the fluid, $T_{p}$ is “the specific heat capacity at constant pressure”, $Q_{r}$ is “the radiation heat flux”, $D_{n}$ is “the coefficient of mass diffusivity”, $T_{n}$ is “the mean fluid temperature”, $K_{T}$ is “the thermal diffusion ratio”.

The equation of incompressible Carreau fluid with variable viscosity as the distance travelled is given by ^7^

$$\overset{´}{\sigma }=-\bar{P}\;\bar{I}+\bar{S}$$ (5)

$$\bar{S}=-\mu \left(T\right)\;\left[1+\left(\frac{n-1}{2}\right)\Gamma^{2}{\bar{\dot{\alpha }}}^{2}\right]\bar{\dot{\alpha }}$$ (6)

Where $\bar{S}$ is “extra stress tensor”, $\bar{P}\;$ "pressure", $\bar{I}$ “identity tensor”, μ “dynamic viscosity”, Γ “time constant”, n “dimensionless power law index” and $\dot{\alpha }\;$ is defined as;

$$\dot{\alpha }=\sqrt{\frac{1}{2}\sum_{i=1}^{3}\sum_{j=1}^{3}{\dot{\alpha }}_{\mathrm{ij}}{\dot{\alpha }}_{\mathrm{ji}}}$$ (7)

The model can be reduced to a Newtonian model n=1orΓ=0 , so we investigate the case for Γ≠0 . To understand how an elastic wall behaves, the equation $L^{**}=\bar{P}-{\bar{p}}_{0}$ , where $L^{**}$ is “an operator”, which is used to represent the motion of stretched membrane with viscosity damping forces such that, see ^3^

$$L^{**}=B\frac{\partial^{4}}{\partial {\bar{Z}}^{4}}-\mathbb{C}\frac{\partial^{2}}{\partial {\bar{Z}}^{2}}+m\frac{\partial^{2}}{\partial {\bar{t}}^{2}}+D\frac{\partial }{\partial \bar{t}}+A_{L}$$

Wall flexural rigidity is denoted by B, longitudinal tension per unit width by ℂ , mass per unit area by m, coefficient of viscous damping by D, and spring stiffness by $A_{L}$ .

This is the equation that controls the properties of a flexible wall canal at $\bar{r}=\bar{r_{2}}$ , is obtained as;

$$\frac{\partial \bar{P}}{\partial \bar{Z}}=\frac{\partial }{\partial \bar{Z}}\left(B\frac{\partial^{4}}{\partial {\bar{Z}}^{4}}-\mathbb{C}\frac{\partial^{2}}{\partial {\bar{Z}}^{2}}+m\frac{\partial^{2}}{\partial {\bar{t}}^{2}}+D\frac{\partial }{\partial \bar{t}}+A_{L}\right)\left(\bar{r_{2}}\right)$$ (8)

## 3. Solution method

For the sake of accuracy in writing the continuity equation and the momentum equations, in addition to the temperature and concentration equations, we use the velocity components $\bar{U_{1}}\left(\bar{R},\bar{Z},\bar{t}\right)$ and $\bar{U_{3}}\left(\bar{R},\bar{Z},\bar{t}\right)$ , which represent the radial and axial velocity components, respectively, in an unsteady two-dimensional flow. The fluid temperature and concentration functions are expressed in terms of $T=T\left(\bar{R},\bar{Z},\bar{t}\right)$ and $∁=∁\left(\bar{R},\bar{Z},\bar{t}\right)$ , respectively. Now, by substituting the governing equations for the problem (1) - (4), we obtain the following system of nonlinear, nonhomogeneous partial differential equations;

$$\frac{\partial \bar{U_{1}}}{\partial \bar{R}}+\frac{\bar{U_{1}}}{\bar{R}}+\frac{\partial \bar{U_{3}}}{\partial \bar{Z}}=0$$ (9)

$$\rho \left(\frac{\partial \bar{U_{1}}}{\partial \bar{t}}+\bar{U_{1}}\frac{\partial \bar{U_{1}}}{\partial \bar{R}}+\bar{U_{3}}\frac{\partial \bar{U_{1}}}{\partial \bar{Z}}\right)=-\frac{\partial \bar{p}}{\partial \bar{R}}+\frac{1}{\bar{R}}\frac{\partial }{\partial \bar{R}}\left(\bar{R}{\bar{S}}_{\bar{R}\bar{R}}\right)+\frac{\partial {\bar{S}}_{\bar{R}\bar{Z}}}{\partial \bar{Z}}$$ (10)

$$\rho \left(\frac{\partial \bar{U_{3}}}{\partial \bar{t}}+\bar{U_{1}}\;\frac{\partial \bar{U_{3}}}{\partial \bar{R}}+\bar{U_{3}}\frac{\partial \bar{U_{3}}}{\partial \bar{Z}}\right)=-\frac{\partial \bar{p}}{\partial \bar{Z}}+\frac{1}{\bar{R}}\frac{\partial }{\partial \bar{R}}\left(\bar{R}{\bar{S}}_{\bar{Z}\bar{R}}\;\right)+\frac{\partial {\bar{S}}_{\bar{Z}\bar{Z}}}{\partial \bar{Z}}+\mathrm{\rho g}\beta_{1}\left(T-T_{0}\right)+\mathrm{\rho g}\beta_{2}\left(∁-{∁}_{0}\right)$$ (11)

$$\frac{\partial T}{\partial \bar{t}}+\bar{U_{1}}\frac{\partial T}{\partial \bar{R}}+\bar{U_{3}}\frac{\partial T}{\partial \bar{Z}}=\frac{T_{n}}{T_{p}\rho }\left(\frac{1}{\bar{R}}\frac{\partial T}{\partial \bar{R}}+\frac{\partial^{2}T}{\partial {\bar{R}}^{2}}+\frac{\partial^{2}T}{\partial {\bar{Z}}^{2}}\right)+\frac{16\sigma_{0}T_{2}^{E}}{3k_{0}T_{p}\;\rho }\left(\frac{1}{\bar{R}}\frac{\partial T}{\partial \bar{R}}+\frac{\partial^{2}T}{\partial {\bar{R}}^{2}}\right)-\frac{q}{T_{p}\;\rho }\left(T-T_{0}\right)$$ (12)

$$\frac{\partial ∁}{\partial \bar{t}}+\bar{U_{1}}\frac{\partial ∁}{\partial \bar{R}}+\bar{U_{3}}\frac{\partial ∁}{\partial \bar{Z}}=D_{n}\left(\frac{1}{\bar{R}}\frac{\partial ∁}{\partial \bar{R}}+\frac{\partial^{2}∁}{\partial {\bar{R}}^{2}}+\frac{\partial^{2}∁}{\partial {\bar{Z}}^{2}}\right)+\frac{D_{n}K_{T}}{T_{n}}\left(\frac{1}{\bar{R}}\frac{\partial T}{\partial \bar{R}}+\frac{\partial^{2}T}{\partial {\bar{R}}^{2}}+\frac{\partial^{2}T}{\partial {\bar{Z}}^{2}}\right)$$ (13)

The component ${\bar{S}}_{\bar{R}\bar{Z}}$ of the shear stress is

$${\bar{S}}_{\bar{R}\bar{Z}}=\mu \left(T\right)\left\{1+\left(\frac{n-1}{2}\right)\Gamma^{2}\left(2\left[{\left(\frac{\partial \bar{U_{1}}}{\partial \bar{R}}\right)}^{2}+{\left(\frac{\bar{U_{1}}}{\bar{R}}\right)}^{2}+{\left(\frac{\partial \bar{U_{3}}}{\partial \bar{Z}}\right)}^{2}\right]+\left[{\left(\frac{\partial \bar{U_{1}}}{\partial \bar{Z}}+\frac{\partial \bar{U_{3}}}{\partial \bar{R}}\right)}^{2}\right]\right)\right\}\left(\frac{\partial \bar{U_{1}}}{\partial \bar{Z}}+\frac{\partial \bar{U_{3}}}{\partial \bar{R}}\right)$$

We use generic and specific frame coordinate transformations as shown below. $\bar{U_{1}}=\bar{u_{1}}$ , $\bar{U_{3}}=\bar{u_{3}}+c$ , $\bar{R}=\bar{r}$ , and $\bar{Z}=\bar{z}$ *.* Substituting these transformations into a system (9) - (13), we get:

$$\frac{\partial \bar{u_{1}}}{\partial \bar{r}}+\frac{\bar{u_{1}}}{\bar{r}}+\frac{\partial \left(\bar{u_{3}}+c\right)}{\partial \bar{z}}=0$$ (14)

$$\rho \left(\frac{\partial \bar{u_{1}}}{\partial \bar{t}}+\bar{u_{1}}\frac{\partial \bar{u_{1}}}{\partial \bar{r}}+\left(\bar{u_{3}}+c\right)\frac{\partial \bar{u_{1}}}{\partial \bar{z}}\right)=-\frac{\partial \bar{p}}{\partial \bar{r}}+\frac{1}{\bar{r}}\frac{\partial }{\partial \bar{r}}\left(\bar{r}{\bar{S}}_{\bar{r}\bar{r}}\right)+\frac{\partial {\bar{S}}_{\bar{r}\bar{z}}}{\partial \bar{z}}$$ (15)

$$\rho \left(\frac{\partial \left(\bar{u_{3}}+c\right)}{\partial \bar{t}}+\bar{u_{1}}\;\frac{\partial \left(\bar{u_{3}}+c\right)}{\partial \bar{r}}+\left(\bar{u_{3}}+c\right)\frac{\partial \left(\bar{u_{3}}+c\right)}{\partial \bar{z}}\right)=-\frac{\partial \bar{p}}{\partial \bar{z}}+\frac{1}{\bar{r}}\frac{\partial }{\partial \bar{r}}\left(\bar{r}{\bar{S}}_{\bar{r}\bar{z}}\;\right)+\frac{\partial {\bar{S}}_{\bar{z}\bar{z}}}{\partial \bar{z}}+\mathrm{\rho g}\beta_{1}\left(T-T_{0}\right)+\mathrm{\rho g}\beta_{2}\left(∁-{∁}_{0}\right)$$ (16)

$$\frac{\partial T}{\partial \bar{t}}+\frac{\bar{u_{1}}\partial T}{\partial \bar{r}}+\left(\bar{u_{3}}+c\right)\frac{\partial T}{\partial \bar{z}}=\frac{T_{n}}{T_{p}\rho }\left(\frac{1}{\bar{r}}\frac{\partial T}{\partial \bar{r}}+\frac{\partial^{2}T}{\partial {\bar{r}}^{2}}+\frac{\partial^{2}T}{\partial {\bar{z}}^{2}}\right)+\frac{16\sigma_{0}T_{2}^{E}}{3k_{0}T_{p}\;\rho }\left(\frac{1}{\bar{r}}\frac{\partial T}{\partial \bar{r}}+\frac{\partial^{2}T}{\partial {\bar{r}}^{2}}\right)-\frac{q}{T_{p}\;\rho }\left(T-T_{0}\right)$$ (17)

$$\frac{\partial ∁}{\partial \bar{t}}+\frac{\bar{u_{1}}\partial ∁}{\partial \bar{r}}+\left(\bar{u_{3}}+c\right)\frac{\partial ∁}{\partial \bar{z}}=D_{n}\left(\frac{1}{\bar{r}}\frac{\partial ∁}{\partial \bar{r}}+\frac{\partial^{2}∁}{\partial {\bar{r}}^{2}}+\frac{\partial^{2}∁}{\partial {\bar{z}}^{2}}\right)+\frac{D_{n}K_{T}}{T_{n}}\left(\frac{1}{\bar{r}}\frac{\partial T}{\partial \bar{r}}+\frac{\partial^{2}T}{\partial {\bar{r}}^{2}}+\frac{\partial^{2}T}{\partial {\bar{z}}^{2}}\right)$$ (18)

The corresponding boundary conditions of the problem are:

$$\begin{array}{c} \bar{u_{1}}=0,\bar{u_{3}}+c=0,T=T_{0},∁={∁}_{1}\;\mathrm{at}\;\bar{r}=\bar{r_{1}}=a_{1}\;\\ \bar{u_{1}}=0,\bar{u_{3}}+c=0,T=T_{1},∁={∁}_{0}\;\mathrm{at}\;\bar{r}=\bar{r_{2}}=a_{2}+b\text{sin}\left(\frac{2\pi }{\gamma }\left(\bar{z}-c\bar{t}\right)\right) \end{array}\}$$ (19)

Where the motion equation with condition of the elastic wall as follows:

$$\begin{array}{l} \frac{\partial }{\partial \bar{z}}\left(B\frac{\partial^{4}}{\partial {\bar{Z}}^{4}}-C\frac{\partial^{2}}{\partial {\bar{Z}}^{2}}+m\frac{\partial^{2}}{\partial {\bar{t}}^{2}}+D\frac{\partial }{\partial \bar{t}}+A_{L}\right)\left(\bar{r_{2}}\right)=\frac{\partial \bar{p}}{\partial \bar{z}}=-\rho \left(\frac{\partial \left(\bar{u_{3}}+c\right)}{\partial \bar{t}}+\bar{u_{1}}\;\frac{\partial \left(\bar{u_{3}}+c\right)}{\partial \bar{r}}+\left(\bar{u_{3}}+c\right)\frac{\partial \left(\bar{u_{3}}+c\right)}{\partial \bar{z}}\right)\\ +\frac{1}{\bar{r}}\frac{\partial }{\partial \bar{r}}\left(\bar{r}{\bar{S}}_{\bar{r}\bar{z}}\;\right)+\frac{\partial {\bar{S}}_{\bar{z}\bar{z}}}{\partial \bar{z}}+\mathrm{\rho g}\beta_{1}\left(T-T_{0}\right)+\mathrm{\rho g}\beta_{2}\left(∁-{∁}_{0}\right) \end{array}$$ (20)

To simplify the governing equations of the problem and to show the important parameters that affect the fluid flow, we introduce the following dimensionless transformations:

u1=u1¯γa2c,u3=u3¯c,r=r¯a2,z=z¯γ,S=a2S¯μvc,p=a22p¯μvcγ,φ=ba2,t=ct¯γ,r2=r2¯a2,r1=r1¯a2,a1a2=ε<1,Ω=qa22μvTp,δ=a2γ,Re=ρca2μv,Pr=μvTpTn,M(H)=μ(T)μv,We=Γca2,α˙=a2α˙¯c,Rn=K0μTp4T2Eσ0,H=T−T0T1−T0,ξ=∁−∁0∁1−∁0,G1=ρgβ1a22(T0−T1)μvs,G2=ρgβ2a22(C1−C0)μvs,S1=ρDnKT(T1−T0)μvTn(C1−C0),S2=μvρDn} (21)

where φ “amplitude ratio”, Re “Reynolds number”, $P_{r}$ “Prandtl number”, $R_{n}$ “thermal radiation parameter”, $S_{2}$ “Schmidt number”, $S_{1}$ “Soret number”, $G_{1}$ “thermal Grashof number”, $G_{2}$ “Solutal Grashof number”, δ “dimensionless wave number”, Ω “heat source/sink parameter”, and $W_{e}$ is the Weissenberg number, $\mu_{v}$ “viscosity constant”.

Substituting Equations (21) into Eqs. (14) - (20), we reformulate the governing equations and accompanying boundary conditions as follows:

$$\left(\frac{c}{\gamma }\right)\left(\frac{\partial u_{1}}{\partial r}+\frac{u_{1}}{r}+\frac{\partial u_{3}}{\partial z}\right)=0$$ (22)

Reδ3(∂u1∂t+u1∂u1∂r+(u3+1)∂u1∂z)=−∂p∂r+δ1r∂∂r(rSrr)+δ2∂Srz∂z (23)

Reδ(∂u3∂t+u1∂u3∂r+(u3+1)∂u3∂z)=−∂p∂z+1r∂∂r(rSrr)+δ∂Szz∂z+G2ξ+G1H (24)

Reδ(∂H∂t+u1∂H∂r+(u3+1)∂H∂z)=1Pr(1r∂H∂r+∂2H∂r2+δ2∂2H∂z2)+43Rn(1r∂H∂r+∂2H∂r2)−ΩH (25)

Reδ(∂ξ∂t+u1∂ξ∂r+(u3+1)∂ξ∂z)=1S2(1r∂ξ∂r+∂2ξ∂r2+δ2∂2ξ∂z2)+S1(1r∂H∂r+∂2H∂r2+δ2∂2H∂z2) (26)

The component $S_{\mathrm{rz}}$ of the shear stress in dimensionless transformation form is

$$S_{\mathrm{rz}}=M\left(H\right)\left\{1+\left(\frac{n-1}{2}\right){\text{We}}^{2}\left(2\delta^{2}\left[{\left(\frac{\partial u_{1}}{\partial r}\right)}^{2}+{\left(\frac{u_{1}}{r}\right)}^{2}+{\left(\frac{\partial u_{3}}{\partial z}\right)}^{2}\right]+\left[{\left(\delta^{2}\frac{\partial u_{1}}{\partial z}+\frac{\partial u_{3}}{\partial r}\right)}^{2}\right]\right)\right\}\left(\delta^{2}a_{2}\frac{\partial u_{1}}{\partial z}+\frac{\partial u_{3}}{\partial r}\right)$$ (27)

The corresponding dimensionless boundary conditions of the problem are

$$\begin{array}{c} u_{1}=0,u_{3}=-1,H=0,\xi =1\;\mathrm{at}\;r=r_{1}=\varepsilon \;\\ u_{1}=0,u_{3}=-1,H=1,\xi =0\;\mathrm{at}\;r=r_{2}=1+\varphi \text{sin}\left(2\pi \left(z-t\right)\right) \end{array}\}$$ (28)

(e1∂5∂z5−e2∂3∂z3+e3∂3∂z∂t2+e4∂2∂z∂t+e5∂∂z)r2=1r∂∂r(rSrz)+δ∂Szz∂z−Reδ(∂u3∂t+u1∂u3∂r+(u3+1)∂u3∂z)+G2ξ+G1H (29)

where $e_{1}=\frac{Ba_{2}^{3}}{\mu_{v}c\gamma^{5}}$ is the flexural stiffness of the wall, $e_{2}=\frac{Ca_{2}^{3}}{\mu_{v}c\gamma^{3}}$ is the longitudinal tension per unit width, $e_{3}=\frac{\mathrm{mc}a_{2}^{3}}{\mu_{v}\gamma^{3}}$ is the mass per unit area, $e_{4}=\frac{Da_{2}^{3}}{\mu_{v}\gamma^{2}}$ is the coefficient of viscid damping, and $e_{4}=\frac{A_{L}a_{2}^{3}}{\mu_{v}\mathrm{c\gamma}}$ is spring stiffness, respectively.

It is very difficult to solve the system of Equations (22) - (27) and (29), so we assume a very small wave number ( δ ≪ 1) concerning the width of the channel to its length. Thus, the system becomes in the following form after abbreviating its writing, taking into account the condition of the flexibility of the outer wall of the flow channel:

$$\left(e_{1}\frac{\partial^{5}}{\;\partial z^{5}}-e_{2}\frac{\partial^{3}}{\;\partial z^{3}}+e_{3}\frac{\;\partial^{3}}{\;\mathrm{\partial z}\;{\mathrm{\partial t}}^{2}}+e_{4}\frac{\partial^{2}}{\;\mathrm{\partial z\partial t}}+e_{5}\frac{\partial }{\mathrm{\partial z}}\right)r_{2}=\frac{1}{r}\frac{\partial }{\partial r}\left(rS_{\mathrm{rz}}\right)+G_{2}\xi +G_{1}H$$ (30)

$$\left(\frac{1}{\mathrm{Pr}}+\frac{4}{3\mathrm{Rn}}\right)\left(\frac{\partial^{2}H}{\partial r^{2}}+\frac{1}{r}\frac{\partial H}{\partial r}\right)-\mathrm{\Omega H}=0$$ (31)

$$\frac{1}{S_{2}}\left(\frac{\partial^{2}\xi }{\partial r^{2}}+\frac{1}{r}\frac{\partial \xi }{\partial r}\right)+S_{1}\left(\frac{\partial^{2}H}{\partial r^{2}}+\frac{1}{r}\frac{\partial H}{\partial r}\right)=0$$ (32)

with

$$S_{\mathrm{rz}}=M\left(\mu \right)\left\{1+\left(\frac{n-1}{2}\right){\text{We}}^{2}{\left(\frac{\partial u_{3}}{\partial r}\right)}^{2}\right\}\left(\frac{\partial u_{3}}{\partial r}\right)$$ (33)

## 4. Solve the problem

This section involves solving the heat and concentration equations, then substituting the result into the velocity equation to solve it.

### 4.1 Temperature and concentration function

The solution to the equations for heat fluid (31) and concentration fluid (32) based on the boundary condition Equation (28) are respectively:

$$\begin{array}{c} H=J\left[0,i\sqrt{A}r\right]B_{1}+Y\left[0,-i\sqrt{A}r\right]B_{2},\\ \xi =B_{4}+B_{3}\log\left[r\right]+\frac{\Sigma \left(-1+I\left[0,\sqrt{Ar^{2}}\right]B_{1}\right)}{A}+\frac{\Sigma \left(Y[0,-i\sqrt{A}r]\right)B_{2}}{A} \end{array}.$$

where $=\frac{\Omega }{\left(\frac{1}{\mathrm{Pr}}+\frac{4}{3\mathrm{Rn}}\right)}$ , $\Sigma =-S_{1}S_{2}A$ , and

$$\begin{array}{l} B_{1}=Y\left[0,-i\sqrt{A}\varepsilon \right]/\left(-J\left[0,i\sqrt{A}\varepsilon \right]Y\left[0,-i\sqrt{A}h\right]+J\left[0,i\sqrt{A}h\right]Y\left[0,-i\sqrt{A}\varepsilon \right]\right),\\ B_{2}=J\left[0,i\sqrt{A}\varepsilon \right]/\left(J\left[0,i\sqrt{A}\varepsilon \right]Y\left[0,-i\sqrt{A}h\right]-J\left[0,i\sqrt{A}h\right]Y\left[0,-i\sqrt{A}\varepsilon \right]\right).\\ B_{3}=-\frac{1}{A\left((\mathrm{Log}\left[h\right]-\mathrm{Log}\left[\varepsilon \right]\right)}\left(A+\Sigma I\left[0,\sqrt{Ah^{2}}\right]B_{1}-\Sigma I\left[0,\sqrt{A\varepsilon^{2}}\right]B_{1}+\Sigma Y\left[0,-i\sqrt{A}h\right]B_{2}-\Sigma Y\left[0,-i\sqrt{A}\varepsilon \right]B_{2}\right),\\ B_{4}=-\frac{1}{A\left(\mathrm{Log}\left[h\right]-\mathrm{Log}\left[\varepsilon \right]\right)}\left(-A\text{Log}\left[h\right]-\Sigma \text{Log}\left[h\right]B_{1}+\Sigma I\left[0,\sqrt{A\varepsilon^{2}}\right]\mathrm{Log}\left[h\right]B_{1}+\Sigma \text{Log}\left[\varepsilon \right]B_{1}-\Sigma I\left[0,\sqrt{Ah^{2}}\right]\mathrm{Log}\left[\varepsilon \right]B_{1}+\Sigma Y\left[0,-i\sqrt{A}\varepsilon \right]\mathrm{Log}\left[h\right]B_{2}-\Sigma Y\left[0,-i\sqrt{A}h\right]\mathrm{Log}\left[\varepsilon \right]B_{2}\right). \end{array}$$

### 4.3 Velocity function

The formula for the velocity equation under the influence of the elasticity of the outer wall of the flow channel, after substituting the shear stress equation in Equation (30), is

$$\left(e_{1}\frac{\partial^{5}}{\;\partial z^{5}}-e_{2}\frac{\partial^{3}}{\;\partial z^{3}}+e_{3}\frac{\;\partial^{3}}{\;\mathrm{\partial z}\;{\mathrm{\partial t}}^{2}}+e_{4}\frac{\partial^{2}}{\;\mathrm{\partial z\partial t}}+e_{5}\frac{\partial }{\mathrm{\partial z}}\right)r_{2}=G_{2}\xi +G_{1}H+\frac{1}{r}\frac{\partial }{\partial r}\left(r\;M\left(H\right)\left\{1+\left(\frac{n-1}{2}\right){W_{e}}^{2}{\left(\frac{\partial u_{3}}{\partial r}\right)}^{2}\right\}\left(\frac{\partial u_{3}}{\partial r}\right)\right)$$ (34)

For the variable viscosity M(H) , we use Reynolds’ model of viscosity $M\left(H\right)=e^{-\mathrm{\alpha H}}$ . By using the Maclaurin series, we have M(H)=1−αH when α≪1 , where α is the coefficient of variable viscosity, the viscosity is fixed at α=0 . Thus, the final form of the velocity equation will be

$$\left(e_{1}\frac{\partial^{5}}{\;\partial z^{5}}-e_{2}\frac{\partial^{3}}{\;\partial z^{3}}+e_{3}\frac{\;\partial^{3}}{\;\mathrm{\partial z}\;{\mathrm{\partial t}}^{2}}+e_{4}\frac{\partial^{2}}{\;\mathrm{\partial z\partial t}}+e_{5}\frac{\partial }{\mathrm{\partial z}}\right)r_{2}=G_{2}\xi +G_{1}H+\frac{1}{r}\frac{\partial }{\partial r}\left(r\;\left(1-\mathrm{\alpha H}\right)\left\{1+\left(\frac{n-1}{2}\right){W_{e}}^{2}{\left(\frac{\partial u_{3}}{\partial r}\right)}^{2}\right\}\left(\frac{\partial u_{3}}{\partial r}\right)\right)$$ (35)

Equation (35) is a non-linear and non-homogeneous partial differential equation, which is difficult to find an exact solution for it, so the perturbation method (twice in terms of $W_{e}$ parameter first, then in terms of the α parameter) will be used to find the solution to the problem, as follows: First let $u_{3}=u_{30}+{W_{e}}^{2}u_{32}+O\left({W_{e}}^{4}\right)$ , and second $u_{3i}=u_{3i0}+\alpha u_{3i1}+O\left(\alpha^{2}\right)$ , for i=0,2 . Therefore, the final form of the velocity function will be $u_{3}=\left(u_{300}+\alpha u_{301}\right)+{W_{e}}^{2}\left(u_{320}+\alpha u_{321}\right)$ .

We will simplify the order of the equations by equating the similar powers of $W_{e}$ and α , respectively.

**4.3.1 Zero order system (** ${W_{e}}^{0}$ **)**

$$\left(e_{1}\frac{\partial^{5}}{\;\partial z^{5}}-e_{2}\frac{\partial^{3}}{\;\partial z^{3}}+e_{3}\frac{\;\partial^{3}}{\;\partial z\;\partial t^{2}}+e_{4}\frac{\partial^{2}}{\;\partial z\partial t}+e_{5}\frac{\partial }{\partial z}\right)r_{2}-G_{2}\xi -G_{1}H=\frac{1}{r}\frac{\partial u_{30}}{\partial r}-\frac{1}{r}\mathrm{\alpha H}\frac{\partial u_{30}}{\partial r}+\frac{\partial^{2}u_{30}}{\partial r^{2}}-\mathrm{\alpha H}\frac{\partial^{2}u_{30}}{\partial r^{2}}-\alpha \frac{\partial H}{\partial r}\left(\frac{\partial u_{30}}{\partial r}\right)$$

(i) **Zero order system (** $\alpha^{0})$

$$\left(e_{1}\frac{\partial^{5}}{\;\partial z^{5}}-e_{2}\frac{\partial^{3}}{\;\partial z^{3}}+e_{3}\frac{\;\partial^{3}}{\;\partial z\;\partial t^{2}}+e_{4}\frac{\partial^{2}}{\;\partial z\partial t}+e_{5}\frac{\partial }{\partial z}\right)r_{2}-G_{2}\xi -G_{1}H=\frac{1}{r}\frac{\partial u_{300}}{\partial r}+\frac{\partial^{2}u_{300}}{\partial r^{2}}$$

The associated boundary conditions $u_{300}\left(r_{1}\right)=u_{300}\left(r_{2}\right)=-1$ .

(ii) **First order system (** α **)**

$$\frac{\partial^{2}u_{301}}{\partial r^{2}}+\frac{1}{r}\frac{\partial u_{301}}{\partial r}=H\left(\frac{1}{r}\frac{\partial u_{300}}{\partial r}+\frac{\partial^{2}u_{300}}{\partial r^{2}}\right)-\frac{\partial H}{\partial r}\left(\frac{\partial u_{300}}{\partial r}\right)$$

The associated boundary conditions $u_{301}\left(r_{1}\right)=u_{301}\left(r_{2}\right)=0$ .

**4.3.2 Second order system (** ${W_{e}}^{2}$ **)**

$$\begin{array}{c} Ο=\frac{1}{r}\frac{\partial u_{31}}{\partial r}+\frac{1}{r}\left(\frac{n-1}{2}\right){\left(\frac{\partial u_{30}}{\partial r}\right)}^{3}-\frac{1}{r}\mathrm{\alpha H}\left(\frac{n-1}{2}\right){\left(\frac{\partial u_{30}}{\partial r}\right)}^{3}-\frac{1}{r}\mathrm{\alpha H}\frac{\partial u_{31}}{\partial r}+\frac{\partial^{2}u_{31}}{\partial r^{2}}+3\left(\frac{n-1}{2}\right)\;\\ {\left(\frac{\partial u_{30}}{\partial r}\right)}^{2}\left(\frac{\partial^{2}u_{30}}{\partial r^{2}}\right)-\mathrm{\alpha H}\frac{\partial^{2}u_{31}}{\partial r^{2}}-3\mathrm{\alpha H}\left(\frac{n-1}{2}\right){\left(\frac{\partial u_{30}}{\partial r}\right)}^{2}\;\left(\frac{\partial^{2}u_{30}}{\partial r^{2}}\right)-\alpha \frac{\partial H}{\partial r}\left(\frac{\partial u_{31}}{\partial r}\right)-\alpha \frac{\partial H}{\partial r}\left(\frac{n-1}{2}\right){\left(\frac{\partial u_{30}}{\partial r}\right)}^{3} \end{array}$$

(i) **Zero order system (** $\alpha^{0})$

$$\frac{\partial^{2}u_{310}}{\partial r^{2}}+\frac{1}{r}\frac{\partial u_{310}}{\partial r}=-\frac{1}{r}\left(\frac{n-1}{2}\right){\left(\frac{\partial u_{300}}{\partial r}\right)}^{3}-3\left(\frac{n-1}{2}\right){\left(\frac{\partial u_{300}}{\partial r}\right)}^{2}\left(\frac{\partial^{2}u_{300}}{\partial r^{2}}\right)$$

The associated boundary conditions $u_{320}\left(r_{1}\right)=u_{320}\left(r_{2}\right)=0$ .

(ii) **First order system (** α **)**

$$\begin{array}{l} \frac{\partial^{2}u_{311}}{\partial r^{2}}+\frac{1}{r}\frac{\partial u_{311}}{\partial r}=H\left(\frac{\partial^{2}u_{310}}{\partial r^{2}}+\frac{1}{r}\frac{\partial u_{310}}{\partial r}\right)+3\left(\frac{1-n}{2}\right)\left(\frac{1}{r}\left(\frac{\partial u_{301}}{\partial r}\right)+\left(\frac{\partial^{2}u_{301}}{\partial r^{2}}\right)\right){\left(\frac{\partial u_{300}}{\partial r}\right)}^{2}-3\left(n-1\right)\\ \left(\frac{\partial u_{300}}{\partial r}\right)\left(\frac{\partial u_{301}}{\partial r}\right)\left(\frac{\partial^{2}u_{300}}{\partial r^{2}}\right)+\left(\frac{n-1}{2}\right)\;H\left(3\left(\frac{\partial^{2}u_{300}}{\partial r^{2}}\right){\left(\frac{\partial u_{300}}{\partial r}\right)}^{2}+\frac{1}{r}{\left(\frac{\partial u_{300}}{\partial r}\right)}^{3}\right)-\frac{\partial H}{\partial r}\left[(\frac{n-1}{2}\right){\left(\frac{\partial u_{300}}{\partial r}\right)}^{3}\\ +\left(\frac{\partial u_{301}}{\partial r}\right)] \end{array}$$

The associated boundary conditions $u_{321}\left(r_{1}\right)=u_{321}\left(r_{2}\right)=0$ .

We obtain very long solutions for the velocity and stream function, known as $u_{3}=\frac{1}{r}\frac{\partial \psi }{\partial r}$ , that mean $\psi =\int r\left(\left(u_{300}+\alpha u_{301}\right)+{W_{e}}^{2}\left(u_{320}+\alpha u_{321}\right)\right)\mathrm{dr}$ . The associated constants can be determined using the associated boundary conditions. Therefore, we will discuss these solutions graphically in the next section.

## 5. Solution analysis

Through the graphs of the fluid velocity function, we discussed and analysed the effect of changing temperature on the viscosity of a Carreau fluid and thus on its velocity through a hollow flexible channel. The program “MATHEMATICA 14” was used in this analysis. The following values were adopted to plot the fluid velocity function: $e_{1}=0.3$ , $e_{2}=0.7$ , $e_{3}=0.5$ , $e_{4}=0.5$ , $e_{5}=0.2$ , Ω=0.5 , $G_{1}=2$ , $G_{2}=1$ , $S_{1}=0.7$ , $S_{2}=0.3$ , $R_{n}=0.5$ , $P_{r}=1.7$ , ε=0.2 , φ=0.15 , $W_{e}=0.2$ , α=0.1 , n=0.3 .

The general shape of the fluid velocity function is a downward-concave curve where the maximum value of the curve is close to the catheter tube around the value of r=0.3 , also the ends of the curve are close to zero at the walls of the channel (the rigid inner and the flexible outer), which matches the boundary condition of the problem. Through Figures 2- 9, we discussed the effect of the important parameters affecting the fluid velocity. We began by examining the elasticity parameters of the outer wall of the flow channel, it was observed when increasing the parameters $e_{1}$ , $e_{2}$ , and $e_{5}$ the velocity fluid increases, as indicated by Figure 2 and Figure 4. In contrast, the parameters $e_{3}$ and $e_{4}$ harmed the velocity fluid, as shown in Figure 3. The temperature and concentration parameters had a mixed effect on the fluid velocity, with increase in the parameters $G_{1}$ , $G_{2}$ , $S_{1}$ , and $S_{2}$ the fluid velocity increases, as shown in Figure 5 and Figure 6. In contrast, increasing parameters Ω , $R_{n}$ , and $P_{r}$ the fluid velocity decreases, as shown in Figure 4 and Figure 7. We also noticed that the effect of the outer wall wave parameter φ is positive on the fluid velocity, while the catheter tube radius ε hurts the fluid velocity, Figure 8. As for the two perturbation parameters $W_{e}$ and α , their effect was clear and positive from Figure 9.

**Figure 1.**
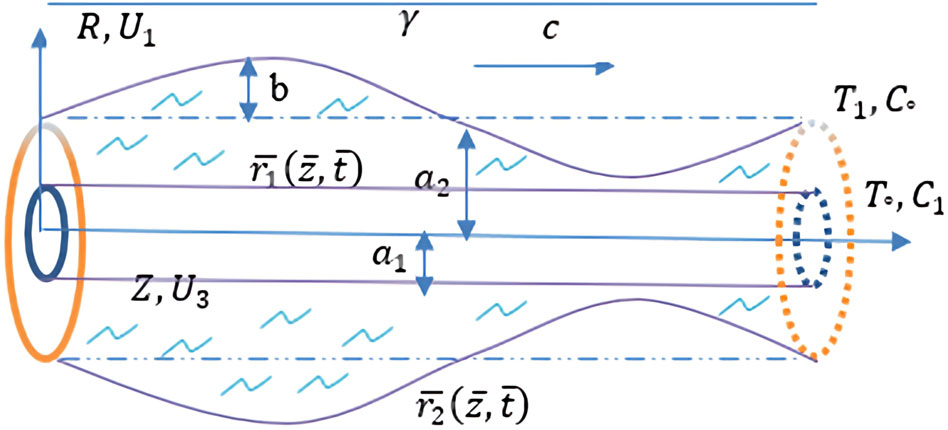
The problem ometry.

**Figure 2.**
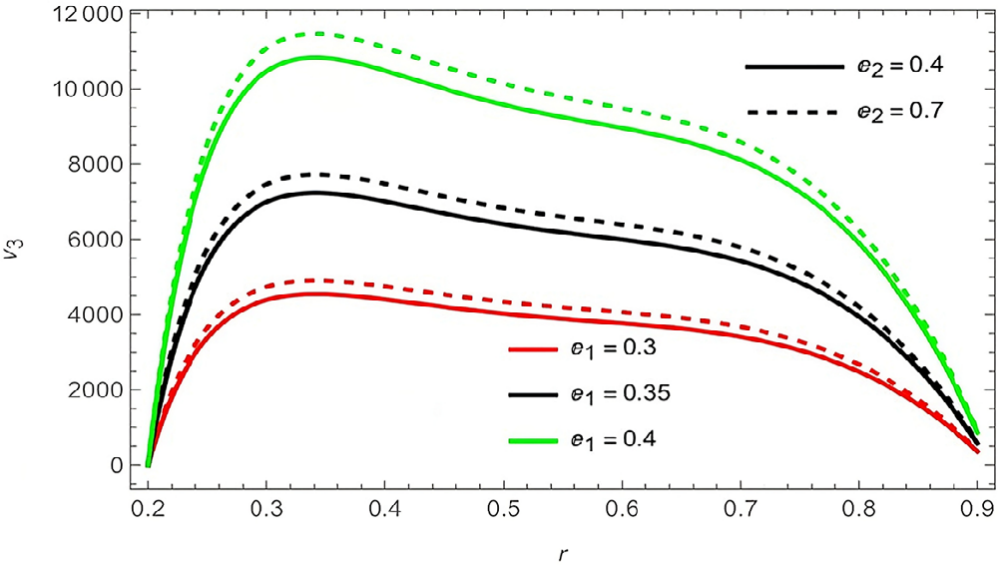
For different values of $e_{1}$ and $e_{2}$ .

**Figure 3.**
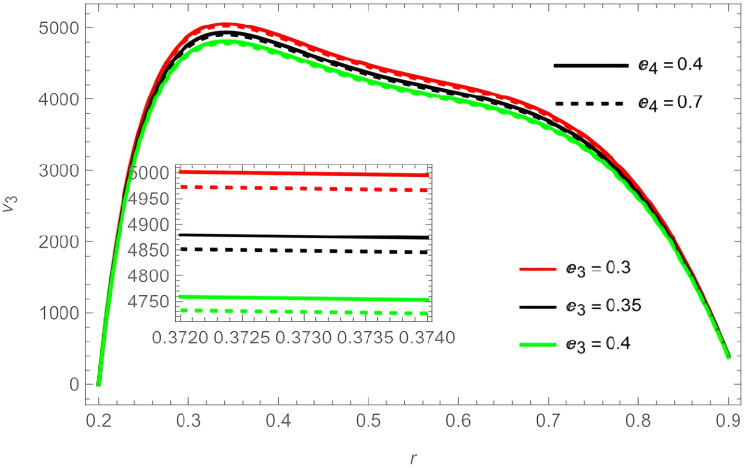
For different values of $e_{3}$ and $e_{4}$ .

**Figure 4.**
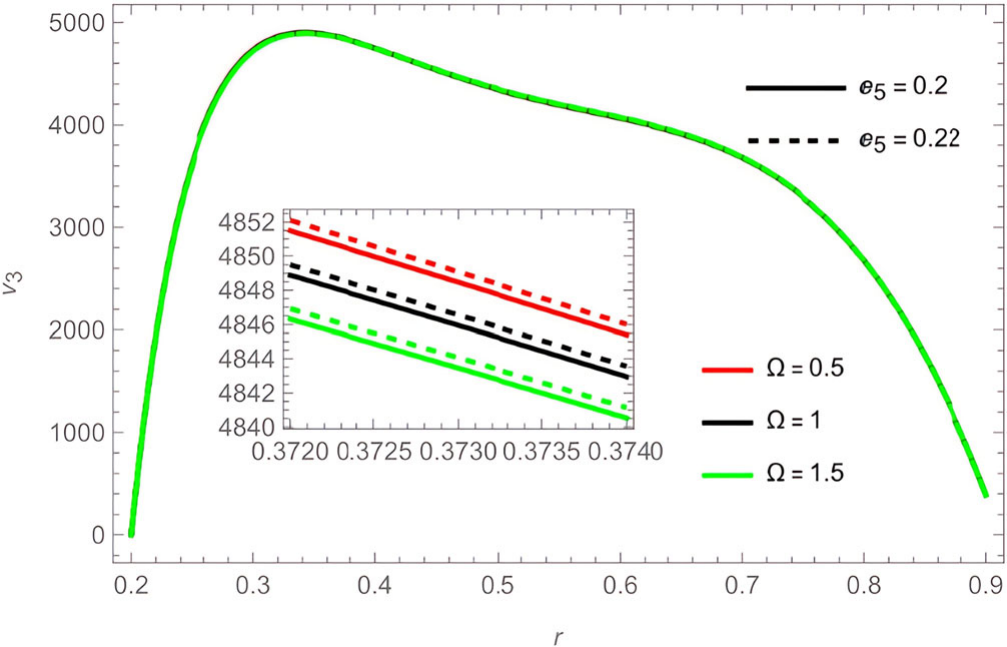
For different values of Ω and $e_{5}$ .

**Figure 5.**
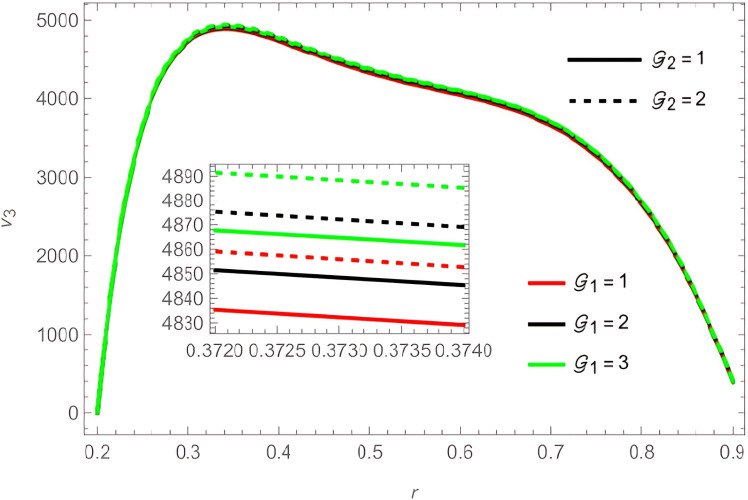
For different values of $G_{1}$ and $G_{2}$ .

**Figure 6.**
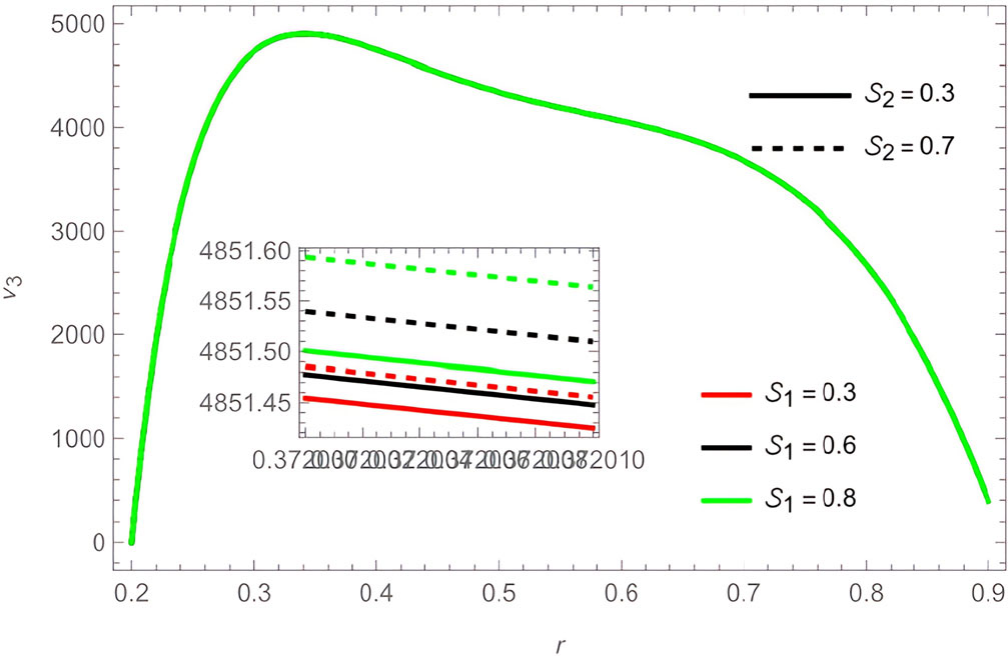
For different values of $S_{1}$ and $S_{2}$ .

**Figure 7.**
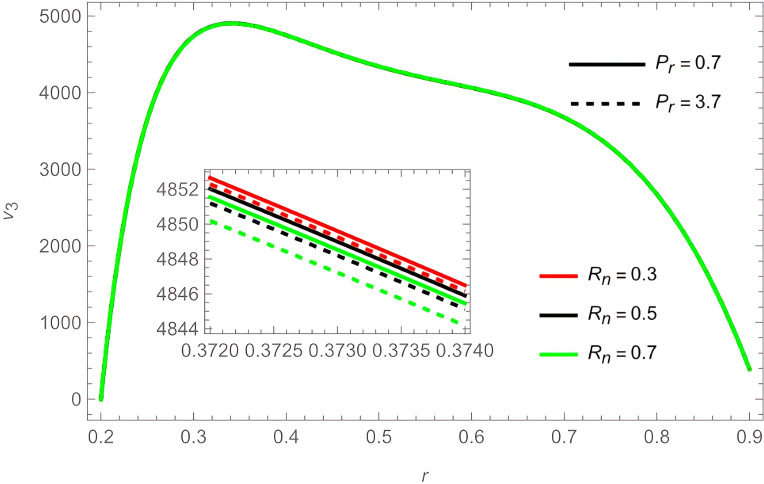
For different values of $R_{n}$ and $P_{r}$ .

**Figure 8.**
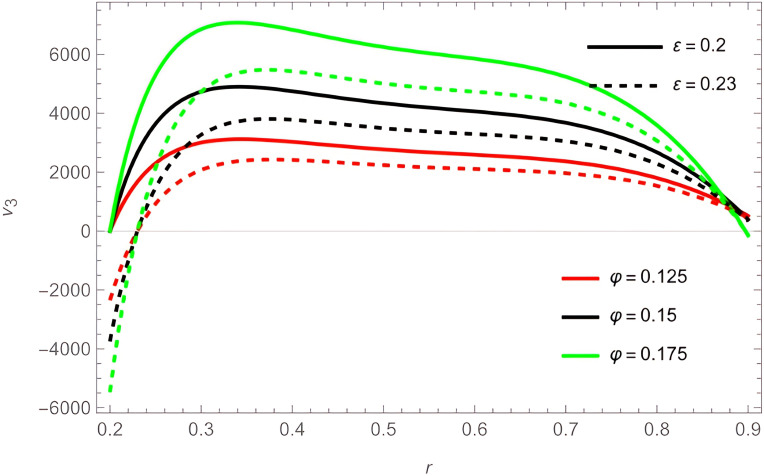
For different values of φ and ε .

**Figure 9.**
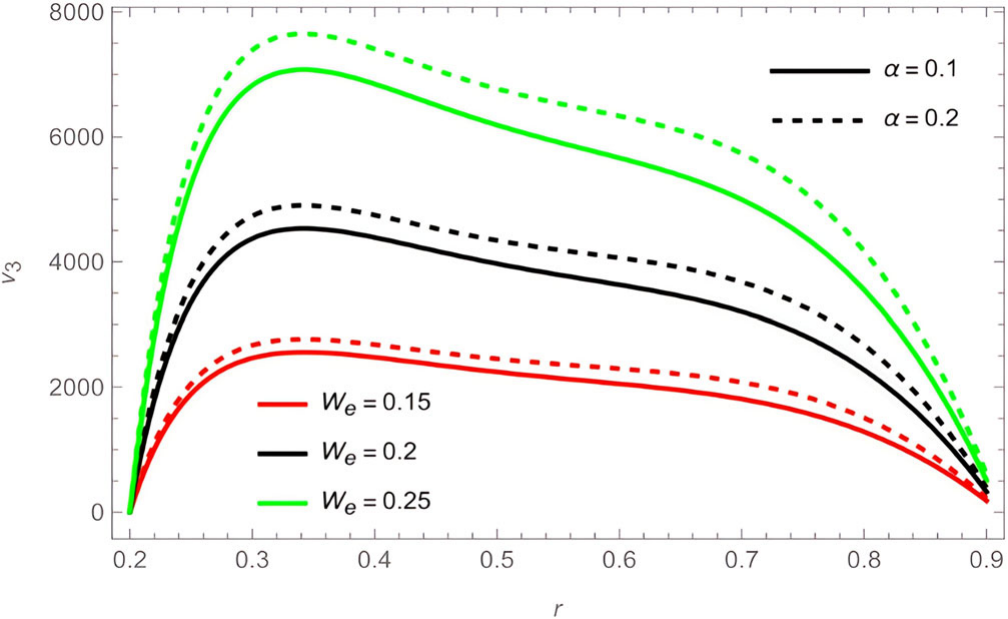
For different values of $W_{e}$ and α .

**Figure 10.**
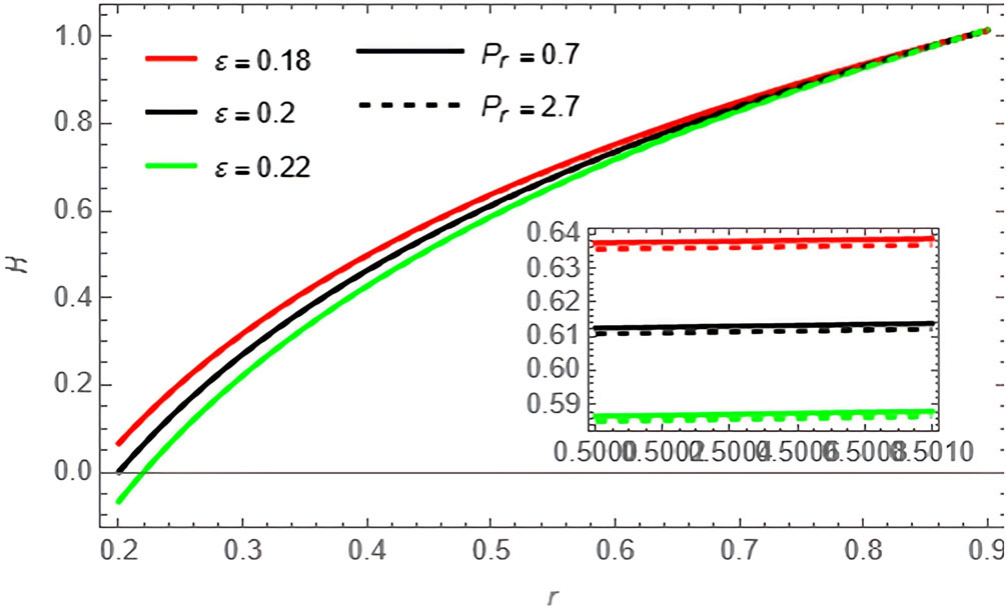
Temperature profile for different values of $P_{r}$ and ε .

**Figure 11.**
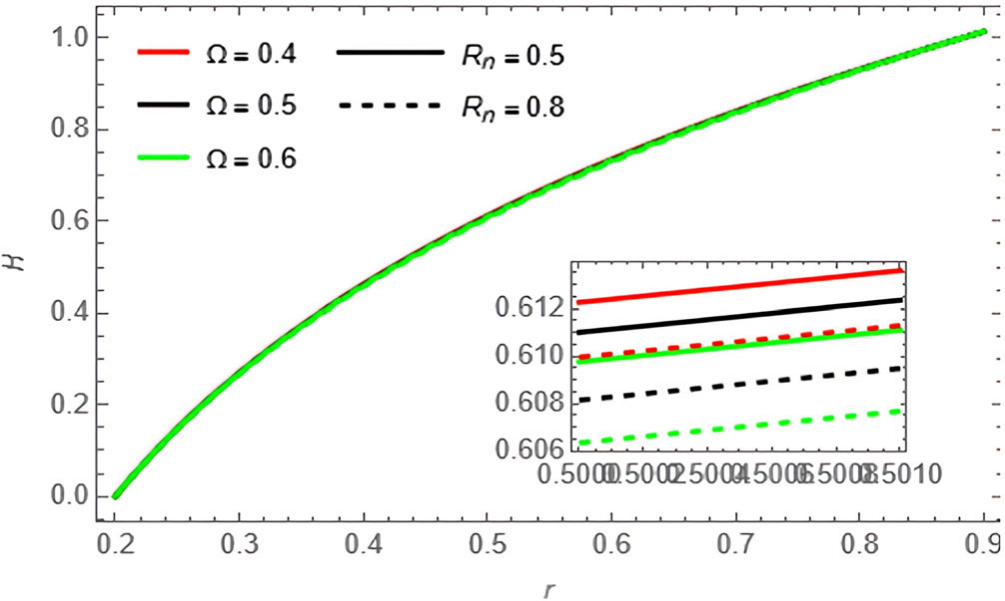
Temperature profile for different values of $R_{n}$ and Ω .

**Figure 12.**
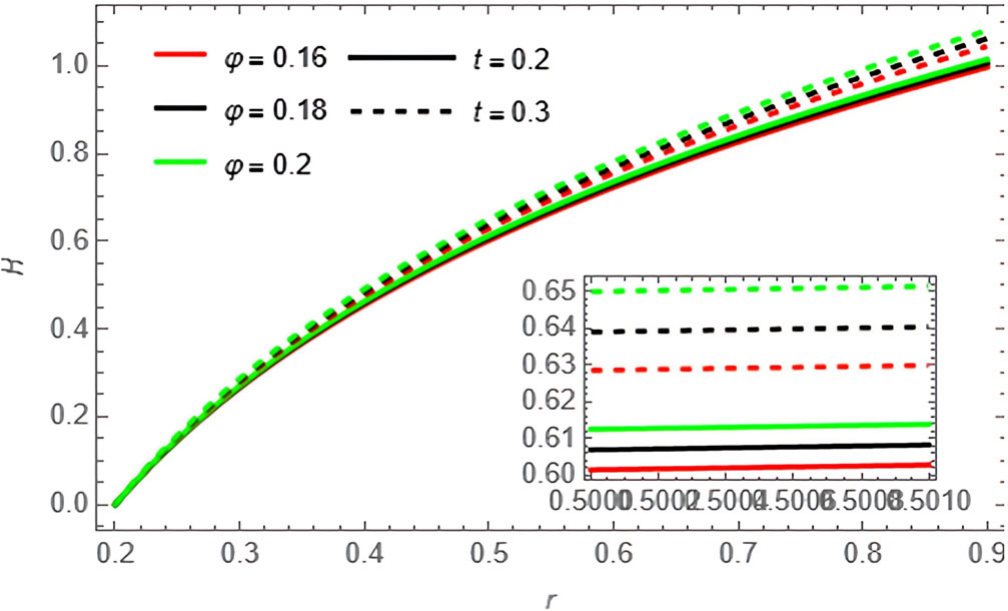
Temperature profile for different values of φ and t .

**Figure 13.**
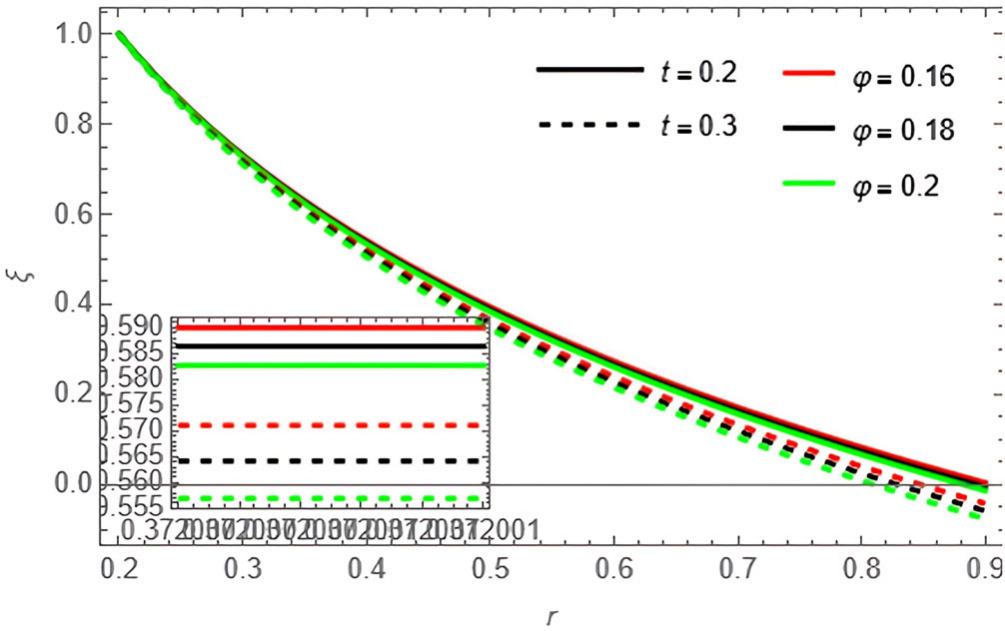
Concentration profile for different values of φ and t .

**Figure 14.**
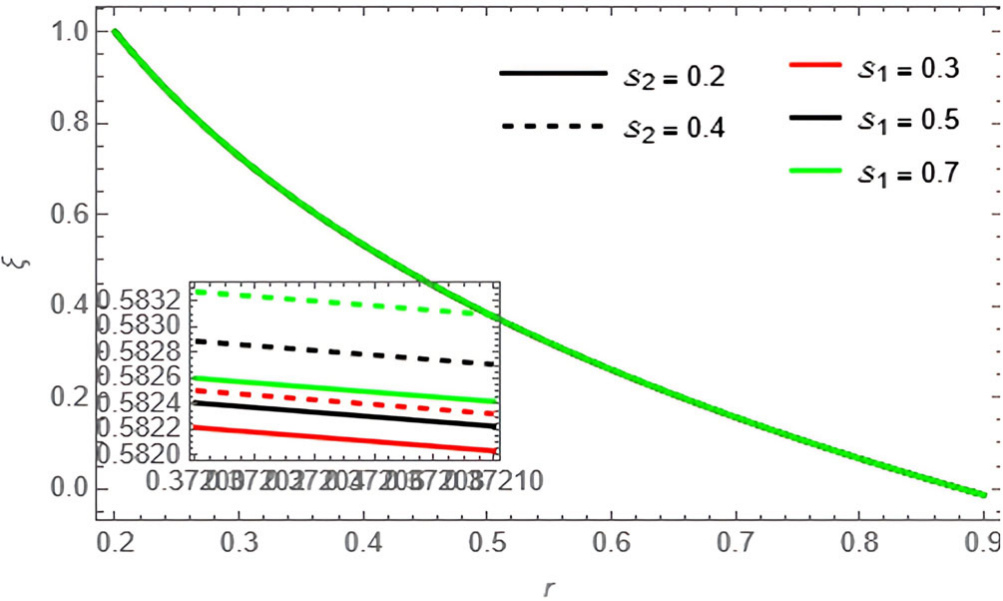
Concentration profile for different values of $S_{1}$ and $S_{2}$ .

**Figure 15.**
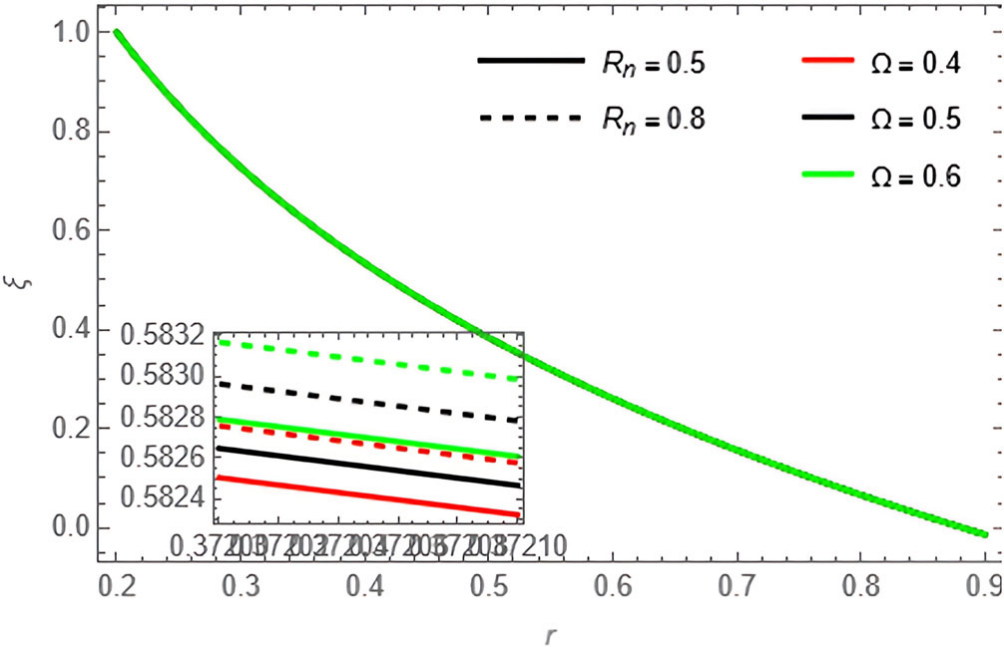
Concentration profile for different values of $R_{n}$ and Ω .

Through the Figures 10- 15, we discuss the temperature and concentration degree function consists of upward-curving lines that are nearly concave, starting from a value close to zero at the left end and gradually increasing until approaching one at the right end. We notice in the two Figures 10 and 11 that the temperature of the fluid decreases with increase of the variables $\varepsilon ,P_{r},\Omega ,$ and $R_{n}$ , respectively, while the opposite is true in Figure 12, where the temperature of the fluid increases with increasing φ,t . In Figure 13, the concentration of the fluid decreases with the increase of the variables φ,t , respectively, while the opposite is true in Figures 14, and 15 where the concentration of the fluid with increasing, $S_{1},S_{2},R_{n}$ , and Ω.

**Figure 16.**
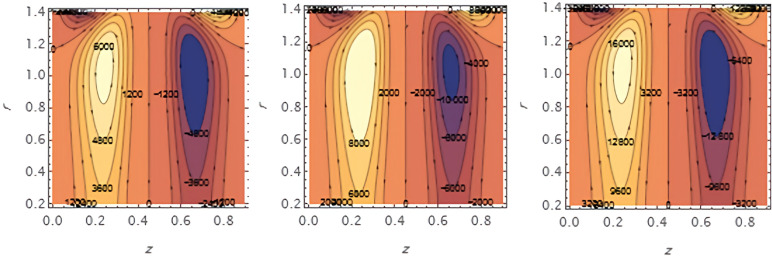
Wave frame streamlines for different values of.

**Figure 17.**
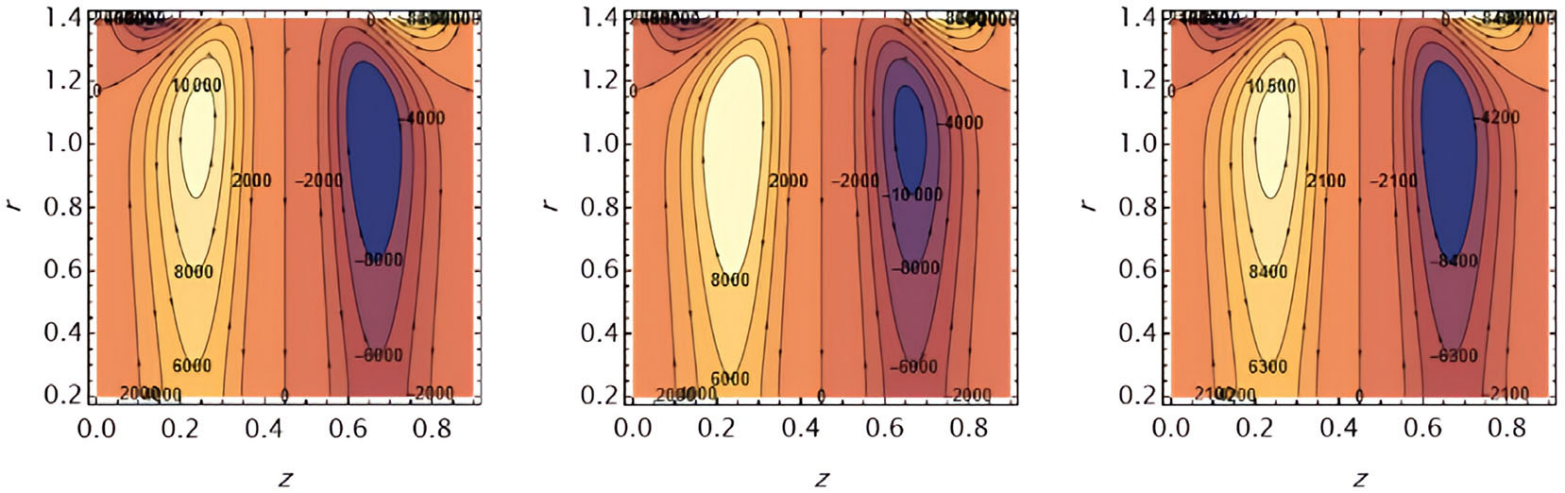
Wave frame streamlines for different values of $e_{2}=0.6,0.7,0.8$ .

**Figure 18.**
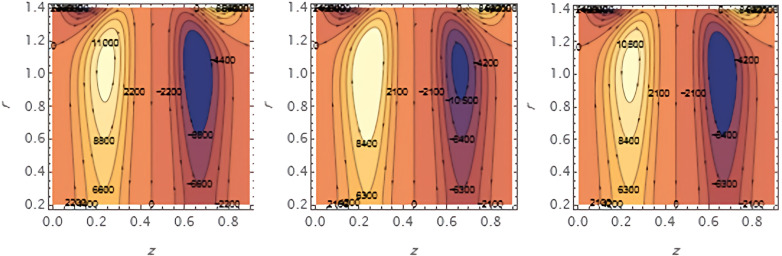
Wave frame streamlines for different values of $e_{3}=0.2,0.3,0.4$ .

**Figure 19.**
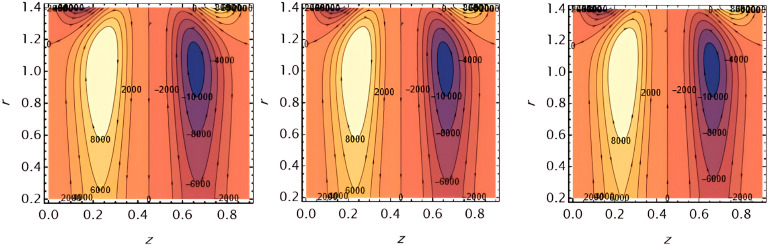
Wave frame streamlines for different values of $G_{2}=1,3,5$ .

**Figure 20.**
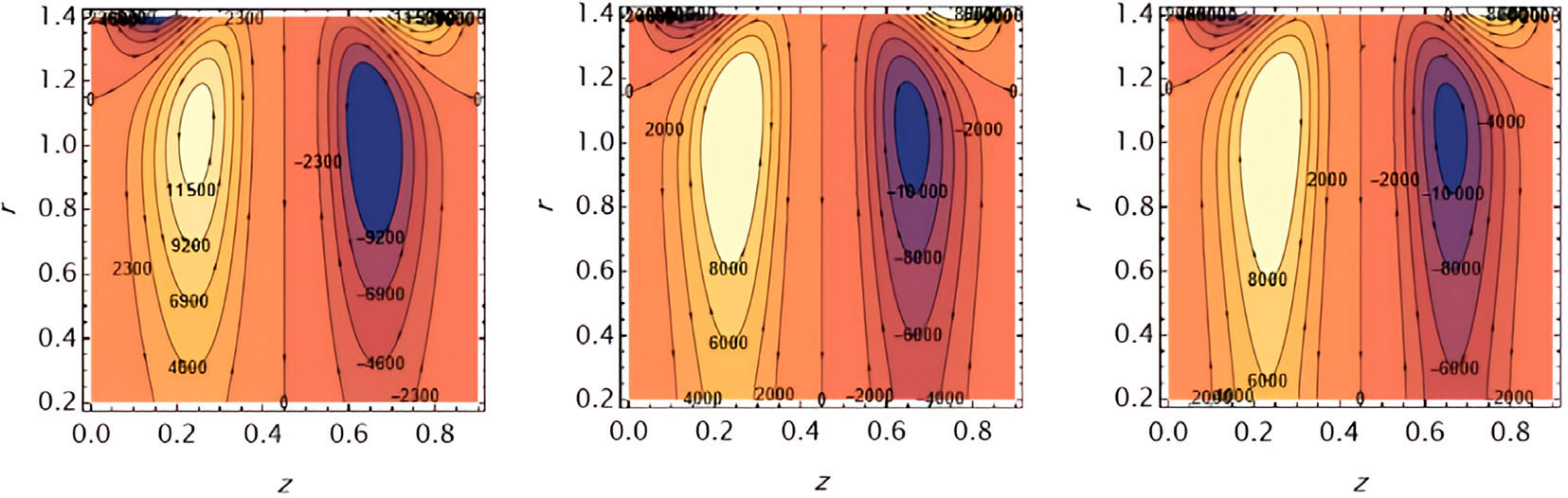
Wave frame streamlines for different values of ε=0.125,0.15,0.2 .

**Figure 21.**
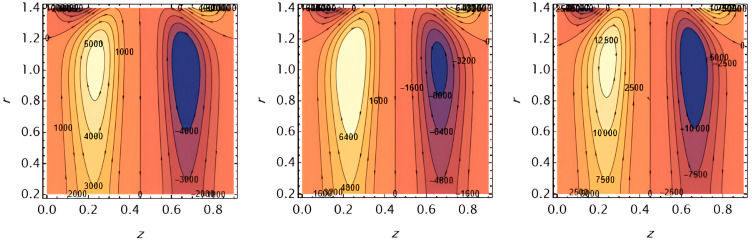
Wave frame streamlines for different values of φ=0.12,0.14,0.16 .

**Figure 22.**
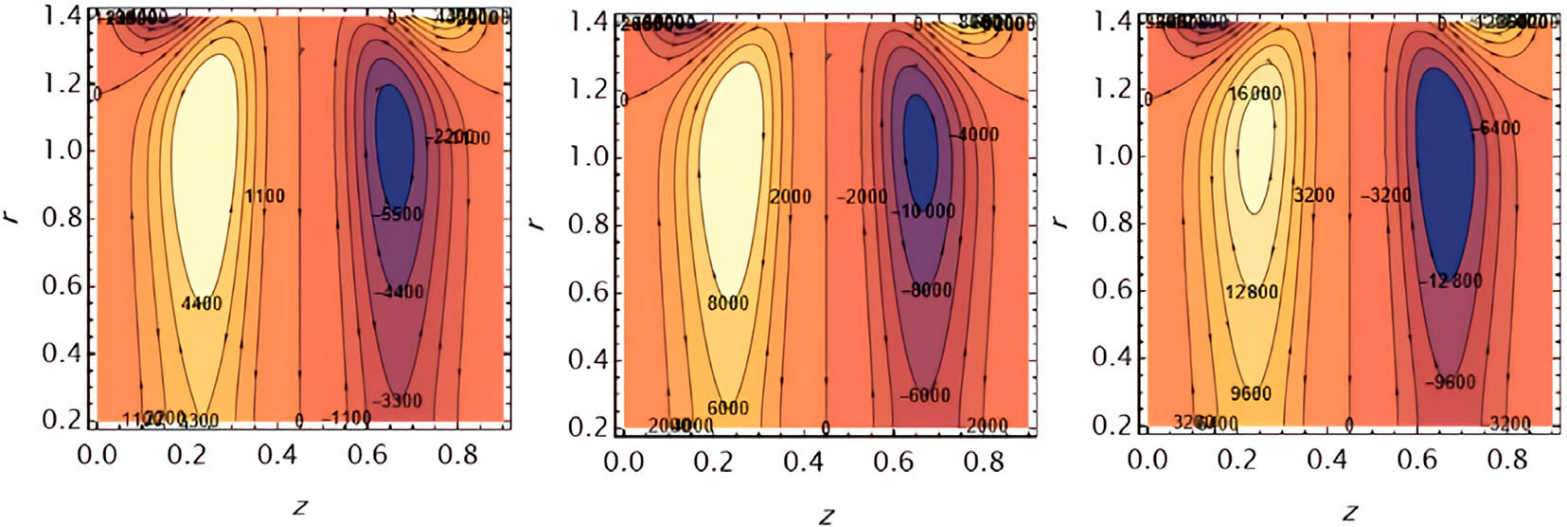
Wave frame streamlines for different values of $W_{e}=0.15,0.2,0.25$ .

**Figure 23.**
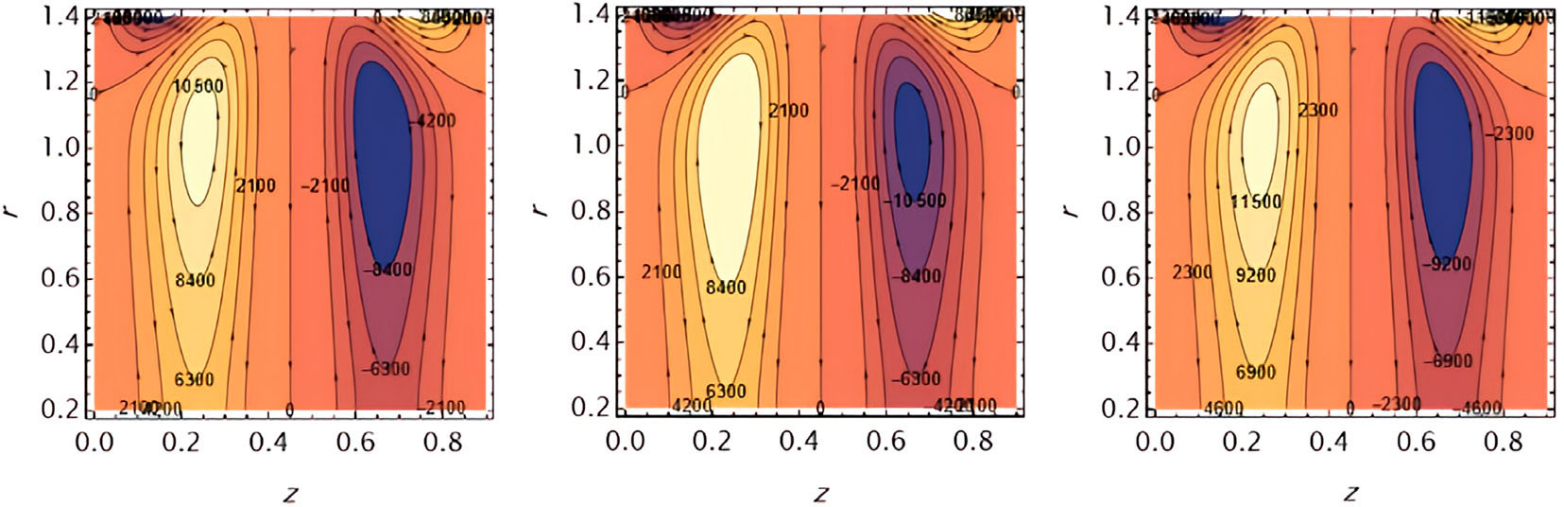
Wave frame streamlines for different values of α=0.125,0.15,0.2 .

Through the Figures 16- 23, we discuss the trapped boluses that arise as a result of the movement of the fluid through the flow channel and thus take the form of bracelets that move in the direction of the fluid movement. We discussed the influence of some important parameters on the boluses and neglected the parameters that did not have a clear effect on them. We observed an increase in the bolus size by increasing the value of $e_{1}$ and $e_{2}$ , Figure 16 and Figure 17, respectively. We observed the opposite effect of the parameter $e_{3}$ on the bolus size as they decreased in size, see Figure 18. While the size of the bolus size expanded with increasing parameters $G_{2}$ , ε , φ , $W_{e}$ , and α , see Figure 19 - Figure 23, respectively.

## Conclusions and Summary

Here we will go over the main points that affect the flow of an incompressible Carreau fluid via a flexible endoscopic hollow tube. Utilizing the perturbation method in conjunction with the MATHEMATICA-14 program, we ascertained the velocity function. We visually examined all the results that came from changing different relevant settings. The key points may be summarized as follows:

1- There is a positive correlation between the growth of $e_{1},e_{2},W_{e},\varphi ,e_{5},\alpha$ , $G_{1},G_{2},S_{1},\;\text{and}\;S_{2}$ while decreases the velocity is due to the increase in parameter $e_{3},e_{4}\;$ , $\;\varepsilon ,P_{r},R_{n}$ and Ω .
2- The trapped bolus expands with an increase $\;e_{1},e_{2},W_{e},\varphi ,\;\text{and}\;\varepsilon ,$ the trapped bolus shrinks increasing the values of $\alpha ,e_{3}.$

3- The following parameters $\;S_{1}$ , $S_{1}$ , $P_{r}$ , $R_{n}$ , Ω , $\;G_{1}$ , $\;e_{5}$ $,\;\text{and}\;e_{4}$ , have no effect on the stream function

## Data Availability

All data underlying the results presented in this study are contained within the article. The figures were generated directly from analytical mathematical expressions, and no external datasets or numerical data were produced or used. Therefore, no datasets requiring deposition in a public repository are associated with this work.
