# Analysis of the peristaltic flow of a variable viscosity Carreau fluid affected by temperature and concentration through an endoscope hollow flexible channel

**DOI:** 10.12688/f1000research.172584.2

**Published:** 2026-03-02

**Authors:** Salwa k. kazem Al-Tamimi, Dheia G. Salih Al-Khafajy

**Affiliations:** 1 Mathematics Department, University of Al-Qadisiya, AL-Qadisiya, AL-Qadisiya, 58001, Iraq; 2 Mathematics Department, University of Al-Qadisiya, Al-Qadisiya, Al-Qadisiya, 58001, Iraq

**Keywords:** Viscous Carreau fluid, peristaltic flow, endoscopic hollow flexible channel.

## Abstract

**Background:**

Peristaltic or undulating flow plays a significant role in various biomedical and industrial processes, where it provides an efficient mechanism for transporting fluids through flexible conduits such as catheters and endoscopic channels. Understanding such flow behavior is essential for improving medical devices and industrial applications involving non-Newtonian fluids.

**Methods:**

This study investigates the peristaltic motion of a Carreau fluid whose viscosity varies with both temperature and concentration within a flexible, axisymmetric channel composed of two overlapping cylindrical tubes. The outer wall of the channel exhibits a sinusoidal wave pattern, simulating a realistic endoscopic configuration. The governing nonlinear, nonhomogeneous partial differential equations were formulated in cylindrical coordinates under the assumption of a long wavelength and low Reynolds number. The equations were transformed into a dimensionless form and solved using the uniform perturbation method. Graphical analyses were performed using Mathematica software.

**Results:**

The results illustrate the combined effects of temperature-dependent and concentration-dependent viscosity on the velocity distribution and pressure gradient within the channel. Increasing temperature and solute concentration were found to enhance fluid velocity and reduce flow resistance.

**Conclusions:**

The study provides a comprehensive understanding of peristaltic transport in variable-viscosity Carreau fluids under realistic physiological conditions. These findings may contribute to optimizing the design and performance of endoscopic and biomedical fluid transport systems.

**Nomenclature**

$r,R,\bar{r}$

: Radial coordinate m

t

: Time s

$a_{1}$

: Inner radius m

b

: Wave amplitude m

c

: Wave propagation speed m/s

$U_{1},U_{3}$

: Radial velocity m/s

P

: Pressure Pa

T

: Temperature K

$M\left(H\right)$

: Dimensionless viscosity function

${∁}_{0}{,∁}_{1}$

: Reference concentrations kg/m ^3^

$D_{n}$

: Mass diffusivity coefficient m ^2^/s

$K_{T}$

: Thermal diffusion ratio (Soret effect) m ^2^/s

g

: Gravitational acceleration m/s ^2^

$\beta_{1}$

: Thermal expansion coefficient K ^−1^

$\beta_{2}$

: Solutal expansion coefficient (kg/m ^3^) ^−1^

B

: Flexural rigidity of the elastic wall N·m

m

: Mass per unit area kg/m ^2^

D

: Viscous damping coefficient N·s/m ^2^

$A_{L}$

: Spring stiffness parameter N/m ^2^ Longitudinal tension per unit width N/m

∁

: Solute concentration kg/m ^3^

q

: Heat source/sink parameter W/m ^3^

$Q_{r}$

: Radiative heat fluxW/m ^2^

K

: Thermal conductivity W/(m·K)

$\dot{\alpha }$

: Magnitude of strain rate tensor s ^−1^

$\bar{z}$

: Axial coordinate m

$a_{2}$

: Outer radius m

γ

: Wavelength m

$u_{1},u_{3}$

: Axial velocity m/s

ρ

: Fluid density kg/m ^3^

$\overset{´}{\sigma }$

: Cauchy stress tensor Pa

$\bar{S}$

: Extra stress tensor Pa

$\mu \left(T\right)$

: Temperature-dependent viscosity Pa·s

$\mu_{v}$

: Reference dynamic viscosity Pa·s

Γ

: Time constant (Carreau fluid) s

$T_{0},T_{1}$

: Reference temperaturesK

$T_{p}$

: Specific heat at constant pressure J/(kg·K)

$T_{n}$

: Mean fluid temperature

ℂ

: Longitudinal tension per unit width N/m

**Dimensionless parameters**

ξ

: Dimensionless concentration

$S_{1}$

: Soret number

$S_{2}$

: Schmidt number

$G_{1}$

: thermal Grashof number

$G_{2}$

: Solutal Grashof number

H

: Dimensionless temperature

Ω

: Heat source/sink parameter

$P_{r}$

: Prandtl number

$R_{n}$

: Thermal radiation parameter

$W_{e}$

: Weissenberg number

n

: Power-law index

Re

: Reynolds number

$r_{1},r_{2}$

: Dimensionless inner and outer radii

δ

: Dimensionless wave number

ε

: Radius ratio

φ

: Amplitude ratio

## 1. Introduction

Peristaltic transport is a fundamental mechanism of fluid motion generated by progressive wave-like contractions of flexible channel walls. This mechanism plays a crucial role in many physiological processes, including blood circulation, gastrointestinal motility, urine transport, and the operation of catheter- and endoscope-based medical devices. In addition to biomedical applications, peristaltic transport is widely utilized in industrial processes involving complex fluids, particularly in situations where direct mechanical pumping is undesirable or impractical.

Most biological and industrial fluids exhibit non-Newtonian behavior, making classical Newtonian fluid models inadequate for realistic flow prediction. Among the various rheological models, the Carreau fluid model has received considerable attention due to its ability to accurately represent shear-thinning behavior and viscosity variation with shear rate. This feature makes it especially suitable for modeling physiological fluids such as blood. Several studies have investigated peristaltic transport under the Carreau fluid assumption. Ali and Hayat ^7^ analyzed pumping characteristics, axial pressure gradients, and trapping phenomena, providing a comparative study between Newtonian and Carreau fluids. Nadeem et al. ^11^ examined peristaltic wave propagation of Carreau fluid in a rectangular duct under the assumptions of long wavelength and low Reynolds number. Ullah et al. ^16^ studied Carreau fluid flow in an elastic tube, emphasizing the significant role of wall flexibility. In addition, peristaltic transport of other non-Newtonian fluids, such as Jeffrey fluid, in flexible channels has been reported by Al-Khalidi and Al-Khafajy, ^6^ highlighting the importance of rheological complexity in peristaltic flow modeling.

Viscosity is one of the most influential physical properties governing fluid motion, particularly in biomedical and food-processing applications. In realistic physiological environments, viscosity is not constant but depends strongly on temperature and concentration variations, which significantly affect flow behavior, heat transfer, and mass diffusion. Several researchers have reported that although fluid velocity exhibits relatively small variations with concentration and spatial location, it increases noticeably with increasing temperature. ^1, 5, 10, 12^ Nadeem et al. ^9^ investigated peristaltic flow of a reactive viscous fluid with temperature-dependent viscosity. Akram and Akbar ^2^ analyzed biological flow of a Carreau nanofluid in an endoscopic system incorporating variable viscosity effects. The influence of temperature and concentration on oscillatory flow in an inclined porous channel was studied by Al-Khafajy and Labban, ^4^ while Al-Delfi and Al-Khafajy ^3^ examined peristaltic transport of Williamson fluid through a hollow flexible channel, accounting for coupled thermal and concentration effects.

Recent developments in peristaltic flow research have emphasized the necessity of incorporating multiple physical mechanisms to achieve realistic modeling of physiological and industrial transport processes. These mechanisms include heat transfer, mass diffusion, nanofluid effects, magnetohydrodynamics (MHD), electroosmosis, and complex wave motions in flexible biomedical geometries. In this context, Tanveer et al. ^13^ investigated flow and heat transfer characteristics in a fallopian tube with a metachronal wave of cilia, demonstrating the crucial role of coordinated wall motion in physiological transport. Tanveer et al. ^14^ further explored dynamic interactions in MHD Jeffrey fluid flow under peristalsis combined with electroosmotic effects and homogeneous–heterogeneous chemical reactions, highlighting the strong coupling between electromagnetic forces, chemical reactions, and non-Newtonian rheology. Moreover, Tanveer et al. ^15^ analyzed the enhancement of heat generation using a ternary hybrid nanofluid in a periodic channel, reflecting the growing interest in advanced nanofluid models for thermal management applications.

Furthermore, Vijayan and Sucharitha ^17^ examined electroosmotic effects on peristaltic transport of a Ree–Eyring nanofluid with double diffusive convection in a symmetric microchannel, demonstrating the strong interaction between electric fields, thermal gradients, and concentration gradients on flow behavior. Jagadesh et al. ^8^ studied convective peristaltic pumping of an MHD Ree–Eyring nanofluid in a chemically reacting flexible divergent channel, incorporating the effects of activation energy and thermal radiation. Their findings emphasize the importance of coupling electromagnetic, chemical, and thermal effects with non-Newtonian rheology in advanced peristaltic flow models.

These studies collectively indicate that realistic modeling of physiological and industrial transport processes requires the simultaneous consideration of thermal effects, concentration gradients, non-Newtonian fluid behavior, and geometrical features such as wall flexibility and internal devices. Despite these extensive efforts, the combined influence of temperature- and concentration-dependent viscosity on the peristaltic flow of a Carreau fluid through a flexible axisymmetric wavy channel containing an internal catheter has not yet been adequately investigated. In particular, the interaction between variable viscosity, channel flexibility, and internal catheter geometry remains insufficiently explored, despite its direct relevance to catheter-based and endoscopic medical applications.

Motivated by this research gap, the present study develops a mathematical model for the peristaltic transport of an incompressible, non-Newtonian Carreau fluid with variable viscosity flowing through a flexible axisymmetric wavy channel containing an internal catheter placed along the centerline. The effects of temperature and concentration variations at the channel walls are incorporated into the viscosity formulation. Under the assumptions of long wavelength and low Reynolds number, the governing equations are simplified and solved analytically. Unlike earlier investigations that considered either constant viscosity or rigid geometries, the present model simultaneously accounts for variable viscosity, wall flexibility, and internal catheter effects, thereby providing a more comprehensive and physiologically realistic representation of peristaltic transport phenomena.

## 2. Mathematical formulation

We study the peristaltic flow of an incompressible Carreau fluid between two cylinders that are in a central location, with an endoscope in the middle of the main channel that has a flexible wall structured like a sine wave. A cylinder’s coordinates are specified by the radius of the channel (R) and the tube’s axis (Z).

The geometry wall of the flow channel form is

$$\begin{array}{l} \bar{r}=\bar{r_{1}}\left(\bar{z},\bar{t}\right)=a_{1},\\ \bar{r}=\bar{r_{2}}\left(\bar{z},\bar{t}\right)=a_{2}+b\text{sin}\left(\frac{2\pi }{\gamma }\left(\bar{z}-c\bar{t}\right)\right)\; \end{array}$$

Here “the unobstructed radius of the pipe” is represented by $\;a_{1}\;$ , the radius of the disturbed tube is represented by $\;a_{2}$ , *b* is “amplitude of the peristaltic wave”, γ is “a wave length”, c is “a wave propagation speed”, and $\bar{t}\;$ is “a time”.

The basic governing equations of the problem system

$$\nabla .\bar{U}=0\;\left(\text{continuity equation}\right)$$ (1)

$$\rho \left(\bar{U.}\nabla \right)\bar{U}=\nabla \overset{´}{\sigma }+\mathrm{\rho g}\beta_{1}\left(T-T_{0}\right)+\mathrm{\rho g}\beta_{2}\left(∁-{∁}_{0}\right)\;\left(\text{momentum equation}\right)$$ (2)

$$T_{p}.\rho \left(\bar{U.}\nabla \right)T=K.\nabla^{2}T-\nabla .Q_{r}-Q\left(T-T_{0}\right)\;\left(\text{temperature equation}\right)$$ (3)

$$\left(\bar{U.}\bar{\nabla }\right)∁=D_{n}\nabla^{2}∁+\frac{D_{n}K_{T}}{T_{n}}\nabla^{2}T\;\left(\text{concentration equation}\right)$$ (4)

Where $\nabla^{2}=\frac{1}{r}\frac{\partial }{\partial r}\left(r\frac{\partial }{\partial r}\right)$ “Laplace operator”, $\bar{U}=\left({\bar{U}}_{1},0,{\bar{U}}_{3}\right)$ is “the velocity field”, ρ is a “density”, $\overset{´}{\;\sigma }$ is “the Cauchy stress tensor”, *T* is “the temperature”, ∁ is a concentration of the fluid, $T_{p}$ is “the specific heat capacity at constant pressure”, $Q_{r}$ is “the radiation heat flux”, $D_{n}$ is “the coefficient of mass diffusivity”, $T_{n}$ is “the mean fluid temperature”, $K_{T}$ is “the thermal diffusion ratio”.

The equation of incompressible Carreau fluid with variable viscosity as the distance travelled is given by ^7^

$$\overset{´}{\sigma }=-\bar{P}\;\bar{I}+\bar{S},$$ (5)

$$\bar{S}=-\mu \left(T\right)\;\left[1+\left(\frac{n-1}{2}\right)\Gamma^{2}{\bar{\dot{\alpha }}}^{2}\right]\bar{\dot{\alpha }}$$ (6)

Where $\bar{S}$ is “extra stress tensor”, $\bar{P}\;$ "pressure", $\bar{I}$ “identity tensor”, μ “dynamic viscosity”, Γ “time constant”, n “dimensionless power law index” and $\dot{\alpha }\;$ is defined as;

$$\dot{\alpha }=\sqrt{\frac{1}{2}\sum_{i=1}^{3}\sum_{j=1}^{3}{\dot{\alpha }}_{\mathrm{ij}}{\dot{\alpha }}_{\mathrm{ji}}}$$ (7)

The model can be reduced to a Newtonian model n=1orΓ=0 , so we investigate the case for Γ≠0 . To understand how an elastic wall behaves, the equation $L^{**}=\bar{P}-{\bar{p}}_{0}$ , where $L^{**}$ is “an operator”, which is used to represent the motion of stretched membrane with viscosity damping forces such that, see ^3^

$$L^{**}=B\frac{\partial^{4}}{\partial {\bar{Z}}^{4}}-\mathbb{C}\frac{\partial^{2}}{\partial {\bar{Z}}^{2}}+m\frac{\partial^{2}}{\partial {\bar{t}}^{2}}+D\frac{\partial }{\partial \bar{t}}+A_{L}$$

Wall flexural rigidity is denoted by B, longitudinal tension per unit width by ℂ , mass per unit area by m, coefficient of viscous damping by D, and spring stiffness by $A_{L}$ .

This is the equation that controls the properties of a flexible wall canal at $\bar{r}=\bar{r_{2}}$ , is obtained as;

$$\frac{\partial \bar{P}}{\partial \bar{Z}}=\frac{\partial }{\partial \bar{Z}}\left(B\frac{\partial^{4}}{\partial {\bar{Z}}^{4}}-\mathbb{C}\frac{\partial^{2}}{\partial {\bar{Z}}^{2}}+m\frac{\partial^{2}}{\partial {\bar{t}}^{2}}+D\frac{\partial }{\partial \bar{t}}+A_{L}\right)\left(\bar{r_{2}}\right)$$ (8)

## 3. Simplified Governing Equations

For the sake of accuracy in writing the continuity equation and the momentum equations, in addition to the temperature and concentration equations, we use the velocity components $\bar{U_{1}}\left(\bar{R},\bar{Z},\bar{t}\right)$ and $\bar{U_{3}}\left(\bar{R},\bar{Z},\bar{t}\right)$ , which represent the radial and axial velocity components, respectively, in an unsteady two-dimensional flow. The fluid temperature and concentration functions are expressed in terms of $T=T\left(\bar{R},\bar{Z},\bar{t}\right)$ and $∁=∁\left(\bar{R},\bar{Z},\bar{t}\right)$ , respectively. Now, by substituting the governing equations for the problem (1) - (4), we obtain the following system of nonlinear, nonhomogeneous partial differential equations;

$$\frac{\partial \bar{U_{1}}}{\partial \bar{R}}+\frac{\bar{U_{1}}}{\bar{R}}+\frac{\partial \bar{U_{3}}}{\partial \bar{Z}}=0$$ (9)

$$\rho \left(\frac{\partial \bar{U_{1}}}{\partial \bar{t}}+\bar{U_{1}}\frac{\partial \bar{U_{1}}}{\partial \bar{R}}+\bar{U_{3}}\frac{\partial \bar{U_{1}}}{\partial \bar{Z}}\right)=-\frac{\partial \bar{p}}{\partial \bar{R}}+\frac{1}{\bar{R}}\frac{\partial }{\partial \bar{R}}\left(\bar{R}{\bar{S}}_{\bar{R}\bar{R}}\right)+\frac{\partial {\bar{S}}_{\bar{R}\bar{Z}}}{\partial \bar{Z}}$$ (10)

$$\rho \left(\frac{\partial \bar{U_{3}}}{\partial \bar{t}}+\bar{U_{1}}\;\frac{\partial \bar{U_{3}}}{\partial \bar{R}}+\bar{U_{3}}\frac{\partial \bar{U_{3}}}{\partial \bar{Z}}\right)=-\frac{\partial \bar{p}}{\partial \bar{Z}}+\frac{1}{\bar{R}}\frac{\partial }{\partial \bar{R}}\left(\bar{R}{\bar{S}}_{\bar{Z}\bar{R}}\;\right)+\frac{\partial {\bar{S}}_{\bar{Z}\bar{Z}}}{\partial \bar{Z}}+\mathrm{\rho g}\beta_{1}\left(T-T_{0}\right)+\mathrm{\rho g}\beta_{2}\left(∁-{∁}_{0}\right)$$ (11)

$$\frac{\partial T}{\partial \bar{t}}+\bar{U_{1}}\frac{\partial T}{\partial \bar{R}}+\bar{U_{3}}\frac{\partial T}{\partial \bar{Z}}=\frac{T_{n}}{T_{p}\rho }\left(\frac{1}{\bar{R}}\frac{\partial T}{\partial \bar{R}}+\frac{\partial^{2}T}{\partial {\bar{R}}^{2}}+\frac{\partial^{2}T}{\partial {\bar{Z}}^{2}}\right)+\frac{16\sigma_{0}T_{2}^{E}}{3k_{0}T_{p}\;\rho }\left(\frac{1}{\bar{R}}\frac{\partial T}{\partial \bar{R}}+\frac{\partial^{2}T}{\partial {\bar{R}}^{2}}\right)-\frac{q}{T_{p}\;\rho }\left(T-T_{0}\right)$$ (12)

$$\frac{\partial ∁}{\partial \bar{t}}+\bar{U_{1}}\frac{\partial ∁}{\partial \bar{R}}+\bar{U_{3}}\frac{\partial ∁}{\partial \bar{Z}}=D_{n}\left(\frac{1}{\bar{R}}\frac{\partial ∁}{\partial \bar{R}}+\frac{\partial^{2}∁}{\partial {\bar{R}}^{2}}+\frac{\partial^{2}∁}{\partial {\bar{Z}}^{2}}\right)+\frac{D_{n}K_{T}}{T_{n}}\left(\frac{1}{\bar{R}}\frac{\partial T}{\partial \bar{R}}+\frac{\partial^{2}T}{\partial {\bar{R}}^{2}}+\frac{\partial^{2}T}{\partial {\bar{Z}}^{2}}\right)$$ (13)

The component ${\bar{S}}_{\bar{R}\bar{Z}}$ of the shear stress is

$${\bar{S}}_{\bar{R}\bar{Z}}=\mu \left(T\right)\left\{1+\left(\frac{n-1}{2}\right)\Gamma^{2}\left(2\left[{\left(\frac{\partial \bar{U_{1}}}{\partial \bar{R}}\right)}^{2}+{\left(\frac{\bar{U_{1}}}{\bar{R}}\right)}^{2}+{\left(\frac{\partial \bar{U_{3}}}{\partial \bar{Z}}\right)}^{2}\right]+\left[{\left(\frac{\partial \bar{U_{1}}}{\partial \bar{Z}}+\frac{\partial \bar{U_{3}}}{\partial \bar{R}}\right)}^{2}\right]\right)\right\}\left(\frac{\partial \bar{U_{1}}}{\partial \bar{Z}}+\frac{\partial \bar{U_{3}}}{\partial \bar{R}}\right)$$

We use generic and specific frame coordinate transformations as shown below. $\bar{U_{1}}=\bar{u_{1}}$ , $\bar{U_{3}}=\bar{u_{3}}+c$ , $\bar{R}=\bar{r}$ , and $\bar{Z}=\bar{z}$ *.* Substituting these transformations into a system (9) - (13), we get:

$$\frac{\partial \bar{u_{1}}}{\partial \bar{r}}+\frac{\bar{u_{1}}}{\bar{r}}+\frac{\partial \left(\bar{u_{3}}+c\right)}{\partial \bar{z}}=0$$ (14)

$$\rho \left(\frac{\partial \bar{u_{1}}}{\partial \bar{t}}+\bar{u_{1}}\frac{\partial \bar{u_{1}}}{\partial \bar{r}}+\left(\bar{u_{3}}+c\right)\frac{\partial \bar{u_{1}}}{\partial \bar{z}}\right)=-\frac{\partial \bar{p}}{\partial \bar{r}}+\frac{1}{\bar{r}}\frac{\partial }{\partial \bar{r}}\left(\bar{r}{\bar{S}}_{\bar{r}\bar{r}}\right)+\frac{\partial {\bar{S}}_{\bar{r}\bar{z}}}{\partial \bar{z}}$$ (15)

$$\rho \left(\frac{\partial \left(\bar{u_{3}}+c\right)}{\partial \bar{t}}+\bar{u_{1}}\;\frac{\partial \left(\bar{u_{3}}+c\right)}{\partial \bar{r}}+\left(\bar{u_{3}}+c\right)\frac{\partial \left(\bar{u_{3}}+c\right)}{\partial \bar{z}}\right)=-\frac{\partial \bar{p}}{\partial \bar{z}}+\frac{1}{\bar{r}}\frac{\partial }{\partial \bar{r}}\left(\bar{r}{\bar{S}}_{\bar{r}\bar{z}}\;\right)+\frac{\partial {\bar{S}}_{\bar{z}\bar{z}}}{\partial \bar{z}}+\mathrm{\rho g}\beta_{1}\left(T-T_{0}\right)+\mathrm{\rho g}\beta_{2}\left(∁-{∁}_{0}\right)$$ (16)

$$\frac{\partial T}{\partial \bar{t}}+\frac{\bar{u_{1}}\partial T}{\partial \bar{r}}+\left(\bar{u_{3}}+c\right)\frac{\partial T}{\partial \bar{z}}=\frac{T_{n}}{T_{p}\rho }\left(\frac{1}{\bar{r}}\frac{\partial T}{\partial \bar{r}}+\frac{\partial^{2}T}{\partial {\bar{r}}^{2}}+\frac{\partial^{2}T}{\partial {\bar{z}}^{2}}\right)+\frac{16\sigma_{0}T_{2}^{E}}{3k_{0}T_{p}\;\rho }\left(\frac{1}{\bar{r}}\frac{\partial T}{\partial \bar{r}}+\frac{\partial^{2}T}{\partial {\bar{r}}^{2}}\right)-\frac{q}{T_{p}\;\rho }\left(T-T_{0}\right)$$ (17)

$$\frac{\partial ∁}{\partial \bar{t}}+\frac{\bar{u_{1}}\partial ∁}{\partial \bar{r}}+\left(\bar{u_{3}}+c\right)\frac{\partial ∁}{\partial \bar{z}}=D_{n}\left(\frac{1}{\bar{r}}\frac{\partial ∁}{\partial \bar{r}}+\frac{\partial^{2}∁}{\partial {\bar{r}}^{2}}+\frac{\partial^{2}∁}{\partial {\bar{z}}^{2}}\right)+\frac{D_{n}K_{T}}{T_{n}}\left(\frac{1}{\bar{r}}\frac{\partial T}{\partial \bar{r}}+\frac{\partial^{2}T}{\partial {\bar{r}}^{2}}+\frac{\partial^{2}T}{\partial {\bar{z}}^{2}}\right)$$ (18)

The corresponding boundary conditions of the problem are:

$$\begin{array}{c} \bar{u_{1}}=0,\bar{u_{3}}+c=0,T=T_{0},∁={∁}_{1}\;\mathrm{at}\;\bar{r}=\bar{r_{1}}=a_{1}\;\\ \bar{u_{1}}=0,\bar{u_{3}}+c=0,T=T_{1},∁={∁}_{0}\;\mathrm{at}\;\bar{r}=\bar{r_{2}}=a_{2}+b\text{sin}\left(\frac{2\pi }{\gamma }\left(\bar{z}-c\bar{t}\right)\right) \end{array}\}$$ (19)

Where the motion equation with condition of the elastic wall as follows:

$$\begin{array}{l} \frac{\partial }{\partial \bar{z}}\left(B\frac{\partial^{4}}{\partial {\bar{Z}}^{4}}-C\frac{\partial^{2}}{\partial {\bar{Z}}^{2}}+m\frac{\partial^{2}}{\partial {\bar{t}}^{2}}+D\frac{\partial }{\partial \bar{t}}+A_{L}\right)\left(\bar{r_{2}}\right)=\frac{\partial \bar{p}}{\partial \bar{z}}=-\rho \left(\frac{\partial \left(\bar{u_{3}}+c\right)}{\partial \bar{t}}+\bar{u_{1}}\;\frac{\partial \left(\bar{u_{3}}+c\right)}{\partial \bar{r}}+\left(\bar{u_{3}}+c\right)\frac{\partial \left(\bar{u_{3}}+c\right)}{\partial \bar{z}}\right)\\ +\frac{1}{\bar{r}}\frac{\partial }{\partial \bar{r}}\left(\bar{r}{\bar{S}}_{\bar{r}\bar{z}}\;\right)+\frac{\partial {\bar{S}}_{\bar{z}\bar{z}}}{\partial \bar{z}}+\mathrm{\rho g}\beta_{1}\left(T-T_{0}\right)+\mathrm{\rho g}\beta_{2}\left(∁-{∁}_{0}\right) \end{array}$$ (20)

To simplify the governing equations of the problem and to show the important parameters that affect the fluid flow, we introduce the following dimensionless transformations ^2, 10^:

u1=u1¯γa2c,u3=u3¯c,r=r¯a2,z=z¯γ,S=a2S¯μvc,p=a22p¯μvcγ,φ=ba2,t=ct¯γ,r2=r2¯a2,r1=r1¯a2,a1a2=ε<1,Ω=qa22μvTp,δ=a2γ,Re=ρca2μv,Pr=μvTpTn,M(H)=μ(T)μv,We=Γca2,α˙=a2α˙¯c,Rn=K0μTp4T2Eσ0,H=T−T0T1−T0,ξ=∁−∁0∁1−∁0,G1=ρgβ1a22(T0−T1)μvs,G2=ρgβ2a22(C1−C0)μvs,S1=ρDnKT(T1−T0)μvTn(C1−C0),S2=μvρDn} (21)

where φ “amplitude ratio”, Re “Reynolds number”, $P_{r}$ “Prandtl number”, $R_{n}$ “thermal radiation parameter”, $S_{2}$ “Schmidt number”, $S_{1}$ “Soret number”, $G_{1}$ “thermal Grashof number”, $G_{2}$ “Solutal Grashof number”, δ “dimensionless wave number”, Ω “heat source/sink parameter”, and $W_{e}$ is the Weissenberg number, $\mu_{v}$ “viscosity constant”.

Substituting Equations (21) into Eqs. (14) - (20), we reformulate the governing equations and accompanying boundary conditions as follows:

$$\left(\frac{c}{\gamma }\right)\left(\frac{\partial u_{1}}{\partial r}+\frac{u_{1}}{r}+\frac{\partial u_{3}}{\partial z}\right)=0$$ (22)

Reδ3(∂u1∂t+u1∂u1∂r+(u3+1)∂u1∂z)=−∂p∂r+δ1r∂∂r(rSrr)+δ2∂Srz∂z (23)

Reδ(∂u3∂t+u1∂u3∂r+(u3+1)∂u3∂z)=−∂p∂z+1r∂∂r(rSrr)+δ∂Szz∂z+G2ξ+G1H (24)

Reδ(∂H∂t+u1∂H∂r+(u3+1)∂H∂z)=1Pr(1r∂H∂r+∂2H∂r2+δ2∂2H∂z2)+43Rn(1r∂H∂r+∂2H∂r2)−ΩH (25)

Reδ(∂ξ∂t+u1∂ξ∂r+(u3+1)∂ξ∂z)=1S2(1r∂ξ∂r+∂2ξ∂r2+δ2∂2ξ∂z2)+S1(1r∂H∂r+∂2H∂r2+δ2∂2H∂z2) (26)

The component $S_{\mathrm{rz}}$ of the shear stress in dimensionless transformation form is

$$S_{\mathrm{rz}}=M\left(H\right)\left\{1+\left(\frac{n-1}{2}\right){\text{We}}^{2}\left(2\delta^{2}\left[{\left(\frac{\partial u_{1}}{\partial r}\right)}^{2}+{\left(\frac{u_{1}}{r}\right)}^{2}+{\left(\frac{\partial u_{3}}{\partial z}\right)}^{2}\right]+\left[{\left(\delta^{2}\frac{\partial u_{1}}{\partial z}+\frac{\partial u_{3}}{\partial r}\right)}^{2}\right]\right)\right\}\left(\delta^{2}a_{2}\frac{\partial u_{1}}{\partial z}+\frac{\partial u_{3}}{\partial r}\right)$$ (27)

The corresponding dimensionless boundary conditions of the problem are

$$\begin{array}{c} u_{1}=0,u_{3}=-1,H=0,\xi =1\;\mathrm{at}\;r=r_{1}=\varepsilon \;\\ u_{1}=0,u_{3}=-1,H=1,\xi =0\;\mathrm{at}\;r=r_{2}=1+\varphi \text{sin}\left(2\pi \left(z-t\right)\right) \end{array}\}$$ (28)

(e1∂5∂z5−e2∂3∂z3+e3∂3∂z∂t2+e4∂2∂z∂t+e5∂∂z)r2=1r∂∂r(rSrz)+δ∂Szz∂z−Reδ(∂u3∂t+u1∂u3∂r+(u3+1)∂u3∂z)+G2ξ+G1H (29)

where $e_{1}=\frac{Ba_{2}^{3}}{\mu_{v}c\gamma^{5}}$ is the flexural stiffness of the wall, $e_{2}=\frac{Ca_{2}^{3}}{\mu_{v}c\gamma^{3}}$ is the longitudinal tension per unit width, $e_{3}=\frac{\mathrm{mc}a_{2}^{3}}{\mu_{v}\gamma^{3}}$ is the mass per unit area, $e_{4}=\frac{Da_{2}^{3}}{\mu_{v}\gamma^{2}}$ is the coefficient of viscid damping, and $e_{4}=\frac{A_{L}a_{2}^{3}}{\mu_{v}\mathrm{c\gamma}}$ is spring stiffness, respectively.

These parameters are selected within physically realistic ranges commonly reported in the literature on peristaltic flow through elastic or compliant channels. ^3, 5^ They represent the resistance of the wall to bending and stretching, its inertia, viscous damping, and elastic support, respectively. The chosen values ensure numerical stability and allow a clear illustration of the essential physical behavior of the system. Moreover, it has been observed that moderate variations in these parameters do not significantly affect the qualitative trends of the results; therefore, a representative set of parameter values is employed throughout the analysis.

It is very difficult to solve the system of Equations (22) - (27) and (29), so we assume a very small wave number ( δ ≪ 1) concerning the width of the channel to its length. Thus, the system becomes in the following form after abbreviating its writing, taking into account the condition of the flexibility of the outer wall of the flow channel:

$$\left(e_{1}\frac{\partial^{5}}{\;\partial z^{5}}-e_{2}\frac{\partial^{3}}{\;\partial z^{3}}+e_{3}\frac{\;\partial^{3}}{\;\mathrm{\partial z}\;{\mathrm{\partial t}}^{2}}+e_{4}\frac{\partial^{2}}{\;\mathrm{\partial z\partial t}}+e_{5}\frac{\partial }{\mathrm{\partial z}}\right)r_{2}=\frac{1}{r}\frac{\partial }{\partial r}\left(rS_{\mathrm{rz}}\right)+G_{2}\xi +G_{1}H$$ (30)

$$\left(\frac{1}{\mathrm{Pr}}+\frac{4}{3\mathrm{Rn}}\right)\left(\frac{\partial^{2}H}{\partial r^{2}}+\frac{1}{r}\frac{\partial H}{\partial r}\right)-\mathrm{\Omega H}=0$$ (31)

$$\frac{1}{S_{2}}\left(\frac{\partial^{2}\xi }{\partial r^{2}}+\frac{1}{r}\frac{\partial \xi }{\partial r}\right)+S_{1}\left(\frac{\partial^{2}H}{\partial r^{2}}+\frac{1}{r}\frac{\partial H}{\partial r}\right)=0$$ (32)

with

$$S_{\mathrm{rz}}=M\left(\mu \right)\left\{1+\left(\frac{n-1}{2}\right){\text{We}}^{2}{\left(\frac{\partial u_{3}}{\partial r}\right)}^{2}\right\}\left(\frac{\partial u_{3}}{\partial r}\right)$$ (33)

## 4. Solution method

This section involves solving the heat and concentration equations, then substituting the result into the velocity equation to solve it.

### 4.1 Temperature and concentration function

The solution to the equations for heat fluid (31) and concentration fluid (32) based on the boundary condition Equation (28) are respectively:

$$\begin{array}{c} H=J\left[0,i\sqrt{A}r\right]B_{1}+Y\left[0,-i\sqrt{A}r\right]B_{2},\\ \xi =B_{4}+B_{3}\log\left[r\right]+\frac{\Sigma \left(-1+I\left[0,\sqrt{Ar^{2}}\right]B_{1}\right)}{A}+\frac{\Sigma \left(Y[0,-i\sqrt{A}r]\right)B_{2}}{A} \end{array}.$$

where $=\frac{\Omega }{\left(\frac{1}{\mathrm{Pr}}+\frac{4}{3\mathrm{Rn}}\right)}$ , $\Sigma =-S_{1}S_{2}A$ , and

$$\begin{array}{l} B_{1}=Y\left[0,-i\sqrt{A}\varepsilon \right]/\left(-J\left[0,i\sqrt{A}\varepsilon \right]Y\left[0,-i\sqrt{A}h\right]+J\left[0,i\sqrt{A}h\right]Y\left[0,-i\sqrt{A}\varepsilon \right]\right),\\ B_{2}=J\left[0,i\sqrt{A}\varepsilon \right]/\left(J\left[0,i\sqrt{A}\varepsilon \right]Y\left[0,-i\sqrt{A}h\right]-J\left[0,i\sqrt{A}h\right]Y\left[0,-i\sqrt{A}\varepsilon \right]\right).\\ B_{3}=-\frac{1}{A\left((\mathrm{Log}\left[h\right]-\mathrm{Log}\left[\varepsilon \right]\right)}\left(A+\Sigma I\left[0,\sqrt{Ah^{2}}\right]B_{1}-\Sigma I\left[0,\sqrt{A\varepsilon^{2}}\right]B_{1}+\Sigma Y\left[0,-i\sqrt{A}h\right]B_{2}-\Sigma Y\left[0,-i\sqrt{A}\varepsilon \right]B_{2}\right),\\ B_{4}=-\frac{1}{A\left(\mathrm{Log}\left[h\right]-\mathrm{Log}\left[\varepsilon \right]\right)}\left(-A\text{Log}\left[h\right]-\Sigma \text{Log}\left[h\right]B_{1}+\Sigma I\left[0,\sqrt{A\varepsilon^{2}}\right]\mathrm{Log}\left[h\right]B_{1}+\Sigma \text{Log}\left[\varepsilon \right]B_{1}-\Sigma I\left[0,\sqrt{Ah^{2}}\right]\mathrm{Log}\left[\varepsilon \right]B_{1}+\Sigma Y\left[0,-i\sqrt{A}\varepsilon \right]\mathrm{Log}\left[h\right]B_{2}-\Sigma Y\left[0,-i\sqrt{A}h\right]\mathrm{Log}\left[\varepsilon \right]B_{2}\right). \end{array}$$

### 4.3 Velocity function

The formula for the velocity equation under the influence of the elasticity of the outer wall of the flow channel, after substituting the shear stress equation in Equation (30), is

$$\left(e_{1}\frac{\partial^{5}}{\;\partial z^{5}}-e_{2}\frac{\partial^{3}}{\;\partial z^{3}}+e_{3}\frac{\;\partial^{3}}{\;\mathrm{\partial z}\;{\mathrm{\partial t}}^{2}}+e_{4}\frac{\partial^{2}}{\;\mathrm{\partial z\partial t}}+e_{5}\frac{\partial }{\mathrm{\partial z}}\right)r_{2}=G_{2}\xi +G_{1}H+\frac{1}{r}\frac{\partial }{\partial r}\left(r\;M\left(H\right)\left\{1+\left(\frac{n-1}{2}\right){W_{e}}^{2}{\left(\frac{\partial u_{3}}{\partial r}\right)}^{2}\right\}\left(\frac{\partial u_{3}}{\partial r}\right)\right)$$ (34)

For the variable viscosity M(H) , we use Reynolds’ model of viscosity $M\left(H\right)=e^{-\mathrm{\alpha H}}$ . By using the Maclaurin series, we have M(H)=1−αH when α≪1 , where α is the coefficient of variable viscosity, the viscosity is fixed at α=0 . Thus, the final form of the velocity equation will be

$$\left(e_{1}\frac{\partial^{5}}{\;\partial z^{5}}-e_{2}\frac{\partial^{3}}{\;\partial z^{3}}+e_{3}\frac{\;\partial^{3}}{\;\mathrm{\partial z}\;{\mathrm{\partial t}}^{2}}+e_{4}\frac{\partial^{2}}{\;\mathrm{\partial z\partial t}}+e_{5}\frac{\partial }{\mathrm{\partial z}}\right)r_{2}=G_{2}\xi +G_{1}H+\frac{1}{r}\frac{\partial }{\partial r}\left(r\;\left(1-\mathrm{\alpha H}\right)\left\{1+\left(\frac{n-1}{2}\right){W_{e}}^{2}{\left(\frac{\partial u_{3}}{\partial r}\right)}^{2}\right\}\left(\frac{\partial u_{3}}{\partial r}\right)\right)$$ (35)

Equation (35) is a non-linear and non-homogeneous partial differential equation, which is difficult to find an exact solution for it, so the perturbation method (twice in terms of $W_{e}$ parameter first, then in terms of the α parameter) will be used to find the solution to the problem, as follows:

let

$$u_{3}=u_{30}+{W_{e}}^{2}u_{31}+O\left({W_{e}}^{4}\right)$$ (36)

After substitution ( 36) into Equation (35), we get:

$$\frac{1}{r}\left[\frac{\partial \left(u_{30}+{W_{e}}^{2}u_{31}\right)}{\partial r}+\left(\frac{n-1}{2}\right){W_{e}}^{2}{\left(\frac{\partial \left(u_{30}+{W_{e}}^{2}u_{31}\right)}{\partial r}\right)}^{3}\right]-\frac{1}{r}\mathrm{\alpha H}\left(\frac{\partial \left(u_{30}+{W_{e}}^{2}u_{31}\right)}{\partial r}\right)-\frac{1}{r}\mathrm{\alpha H}\left(\frac{n-1}{2}\right){W_{e}}^{2}{\left(\frac{\partial \left(u_{30}+{W_{e}}^{2}u_{31}\right)}{\partial r}\right)}^{3}+\frac{\partial^{2}\left(u_{30}+{W_{e}}^{2}u_{31}\right)}{\partial r^{2}}+3\left(\frac{n-1}{2}\right){W_{e}}^{2}{\left(\frac{\partial \left(u_{30}+{W_{e}}^{2}u_{31}\right)}{\partial r}\right)}^{2}\left(\frac{\partial^{2}\left(u_{30}+{W_{e}}^{2}u_{31}\right)}{\partial r^{2}}\right)-\mathrm{\alpha H}\left[\frac{\partial^{2\left(u_{30}+{W_{e}}^{2}u_{31}\right)}}{\partial r^{2}}+3\left(\frac{n-1}{2}\right){W_{e}}^{2}{\left(\frac{\partial \left(u_{30}+{W_{e}}^{2}u_{31}\right)}{\partial r}\right)}^{2}\left(\frac{\partial^{2}\left(u_{30}+{W_{e}}^{2}u_{31}\right)}{\partial r^{2}}\right)\right]-\alpha \frac{\partial H}{\partial r}\left[\frac{\partial \left(u_{30}+{W_{e}}^{2}u_{31}\right)}{\partial r}+\left(\frac{n-1}{2}\right){W_{e}}^{2}{\left(\frac{\partial \left(u_{30}+{W_{e}}^{2}u_{31}\right)}{\partial r}\right)}^{3}\right]=\left(e_{1}\frac{\partial^{5}}{\;\partial z^{5}}-e_{2}\frac{\partial^{3}}{\;\partial z^{3}}+e_{3}\frac{\;\partial^{3}}{\;\partial z\;\partial t^{2}}+e_{4}\frac{\partial^{2}}{\;\partial z\partial t}+e_{5}\frac{\partial }{\partial z}\right)r_{2}-G_{2}\xi -G_{1}H$$ (37)

**4.3.1 Zero order system (** ${W_{e}}^{0}$ **)**

$$\left(e_{1}\frac{\partial^{5}}{\;\partial z^{5}}-e_{2}\frac{\partial^{3}}{\;\partial z^{3}}+e_{3}\frac{\;\partial^{3}}{\;\partial z\;\partial t^{2}}+e_{4}\frac{\partial^{2}}{\;\partial z\partial t}+e_{5}\frac{\partial }{\partial z}\right)r_{2}-G_{2}\xi -G_{1}H=\frac{1}{r}\frac{\partial u_{30}}{\partial r}-\frac{1}{r}\mathrm{\alpha H}\frac{\partial u_{30}}{\partial r}+\frac{\partial^{2}u_{30}}{\partial r^{2}}-\mathrm{\alpha H}\frac{\partial^{2}u_{30}}{\partial r^{2}}-\alpha \frac{\partial H}{\partial r}\left(\frac{\partial u_{30}}{\partial r}\right)$$ (38)

Substation with an equation perturbation

$$u_{30}=u_{300}+\alpha u_{301}+O\left(\alpha^{2}\right)$$ (39)

After substitution ( 39) into Equation (38), we get:

$$\left(e_{1}\frac{\partial^{5}}{\;\partial z^{5}}-e_{2}\frac{\partial^{3}}{\;\partial z^{3}}+e_{3}\frac{\;\partial^{3}}{\;\partial z\;\partial t^{2}}+e_{4}\frac{\partial^{2}}{\;\partial z\partial t}+e_{5}\frac{\partial }{\partial z}\right)r_{2}-G_{2}\xi -G_{1}H=\frac{1}{r}\frac{\partial \left(u_{300}+\alpha u_{301}\right)}{\partial r}-\frac{1}{r}\mathrm{\alpha H}\frac{\partial \left(u_{300}+\alpha u_{301}\right)}{\partial r}+\frac{\partial^{2}\left(u_{300}+\alpha u_{301}\right)}{\partial r^{2}}-\mathrm{\alpha H}\frac{\partial^{2}\left(u_{300}+\alpha u_{301}\right)}{\partial r^{2}}-\alpha \frac{\partial H}{\partial r}\left(\frac{\partial \left(u_{300}+\alpha u_{301}\right)}{\partial r}\right)$$ (40)

(i) **Zero order system (** $\alpha^{0})$

$$\left(e_{1}\frac{\partial^{5}}{\;\partial z^{5}}-e_{2}\frac{\partial^{3}}{\;\partial z^{3}}+e_{3}\frac{\;\partial^{3}}{\;\partial z\;\partial t^{2}}+e_{4}\frac{\partial^{2}}{\;\partial z\partial t}+e_{5}\frac{\partial }{\partial z}\right)r_{2}-G_{2}\xi -G_{1}H=\frac{1}{r}\frac{\partial u_{300}}{\partial r}+\frac{\partial^{2}u_{300}}{\partial r^{2}}$$

The associated boundary conditions $u_{300}\left(r_{1}\right)=u_{300}\left(r_{2}\right)=-1$ .

(ii) **First order system (** α **)**

$$\frac{\partial^{2}u_{301}}{\partial r^{2}}+\frac{1}{r}\frac{\partial u_{301}}{\partial r}=H\left(\frac{1}{r}\frac{\partial u_{300}}{\partial r}+\frac{\partial^{2}u_{300}}{\partial r^{2}}\right)-\frac{\partial H}{\partial r}\left(\frac{\partial u_{300}}{\partial r}\right)$$

The associated boundary conditions $u_{301}\left(r_{1}\right)=u_{301}\left(r_{2}\right)=0$ .

**4.3.2 Second order system (** ${W_{e}}^{2}$ **)**

$$\begin{array}{c} Ο=\frac{1}{r}\frac{\partial u_{31}}{\partial r}+\frac{1}{r}\left(\frac{n-1}{2}\right){\left(\frac{\partial u_{30}}{\partial r}\right)}^{3}-\frac{1}{r}\mathrm{\alpha H}\left(\frac{n-1}{2}\right){\left(\frac{\partial u_{30}}{\partial r}\right)}^{3}-\frac{1}{r}\mathrm{\alpha H}\frac{\partial u_{31}}{\partial r}+\frac{\partial^{2}u_{31}}{\partial r^{2}}+3\left(\frac{n-1}{2}\right)\;\\ {\left(\frac{\partial u_{30}}{\partial r}\right)}^{2}\left(\frac{\partial^{2}u_{30}}{\partial r^{2}}\right)-\mathrm{\alpha H}\frac{\partial^{2}u_{31}}{\partial r^{2}}-3\mathrm{\alpha H}\left(\frac{n-1}{2}\right){\left(\frac{\partial u_{30}}{\partial r}\right)}^{2}\;\left(\frac{\partial^{2}u_{30}}{\partial r^{2}}\right)-\alpha \frac{\partial H}{\partial r}\left(\frac{\partial u_{31}}{\partial r}\right)-\alpha \frac{\partial H}{\partial r}\left(\frac{n-1}{2}\right){\left(\frac{\partial u_{30}}{\partial r}\right)}^{3} \end{array}$$ (41)

Substation with an equation perturbation

$$u_{31}=u_{310}+\alpha u_{311}+O\left(\alpha^{2}\right)$$ (42)

After substitution ( 42) into Equation (41), we get:

$$Ο=\frac{1}{r}\frac{\partial \left(u_{310}+\alpha u_{311}\right)}{\partial r}+\frac{1}{r}\left(\frac{n-1}{2}\right)\left[{\left(\frac{\partial u_{300}}{\partial r}\right)}^{3}+3\alpha {\left(\frac{\partial u_{300}}{\partial r}\right)}^{2}\left(\frac{\partial u_{301}}{\partial r}\right)\right]-\frac{1}{r}\mathrm{\alpha H}\left(\frac{n-1}{2}\right)\left[{\left(\frac{\partial u_{300}}{\partial r}\right)}^{3}+3\alpha {\left(\frac{\partial u_{300}}{\partial r}\right)}^{2}\left(\frac{\partial u_{301}}{\partial r}\right)\right]-\frac{1}{r}\mathrm{\alpha H}\frac{\partial \left(u_{310}+\alpha u_{311}\right)}{\partial r}+\frac{\partial^{2}\left(u_{310}+\alpha u_{311}\right)}{\partial r^{2}}+3\left(\frac{n-1}{2}\right)\left[{\left(\frac{\partial u_{300}}{\partial r}\right)}^{2}\left(\frac{\partial^{2}u_{300}}{\partial r^{2}}\right)+2\alpha \left(\frac{\partial u_{300}}{\partial r}\right)\left(\frac{\partial u_{301}}{\partial r}\right)\left(\frac{\partial^{2}u_{300}}{\partial r^{2}}\right)+\alpha {\left(\frac{\partial u_{300}}{\partial r}\right)}^{2}\left(\frac{\partial^{2}u_{301}}{\partial r^{2}}\right)\right]-\mathrm{\alpha H}\frac{\partial^{2}\left(u_{310}+\alpha u_{311}\right)}{\partial r^{2}}-3\mathrm{\alpha H}\left(\frac{n-1}{2}\right)$$

$$\left[{\left(\frac{\partial u_{300}}{\partial r}\right)}^{2}\left(\frac{\partial^{2}u_{300}}{\partial r^{2}}\right)+2\alpha \left(\frac{\partial u_{300}}{\partial r}\right)\left(\frac{\partial u_{301}}{\partial r}\right)\left(\frac{\partial^{2}u_{300}}{\partial r^{2}}\right)+\alpha {\left(\frac{\partial u_{300}}{\partial r}\right)}^{2}\left(\frac{\partial^{2}u_{301}}{\partial r^{2}}\right)\right]-\alpha \frac{\partial H}{\partial r}\left(\frac{\partial \left(u_{310}+\alpha u_{311}\right)}{\partial r}\right)-\alpha \frac{\partial H}{\partial r}\left(\frac{n-1}{2}\right)\left[{\left(\frac{\partial u_{300}}{\partial r}\right)}^{3}+3\alpha {\left(\frac{\partial u_{300}}{\partial r}\right)}^{2}\left(\frac{\partial u_{301}}{\partial r}\right)\right]$$ (43)

(i) **Zero order system (** $\alpha^{0})$

$$\frac{\partial^{2}u_{310}}{\partial r^{2}}+\frac{1}{r}\frac{\partial u_{310}}{\partial r}=-\frac{1}{r}\left(\frac{n-1}{2}\right){\left(\frac{\partial u_{300}}{\partial r}\right)}^{3}-3\left(\frac{n-1}{2}\right){\left(\frac{\partial u_{300}}{\partial r}\right)}^{2}\left(\frac{\partial^{2}u_{300}}{\partial r^{2}}\right)$$

The associated boundary conditions $u_{320}\left(r_{1}\right)=u_{320}\left(r_{2}\right)=0$ .

(ii) **First order system (** α **)**

$$\begin{array}{l} \frac{\partial^{2}u_{311}}{\partial r^{2}}+\frac{1}{r}\frac{\partial u_{311}}{\partial r}=H\left(\frac{\partial^{2}u_{310}}{\partial r^{2}}+\frac{1}{r}\frac{\partial u_{310}}{\partial r}\right)+3\left(\frac{1-n}{2}\right)\left(\frac{1}{r}\left(\frac{\partial u_{301}}{\partial r}\right)+\left(\frac{\partial^{2}u_{301}}{\partial r^{2}}\right)\right){\left(\frac{\partial u_{300}}{\partial r}\right)}^{2}-3\left(n-1\right)\\ \left(\frac{\partial u_{300}}{\partial r}\right)\left(\frac{\partial u_{301}}{\partial r}\right)\left(\frac{\partial^{2}u_{300}}{\partial r^{2}}\right)+\left(\frac{n-1}{2}\right)\;H\left(3\left(\frac{\partial^{2}u_{300}}{\partial r^{2}}\right){\left(\frac{\partial u_{300}}{\partial r}\right)}^{2}+\frac{1}{r}{\left(\frac{\partial u_{300}}{\partial r}\right)}^{3}\right)-\frac{\partial H}{\partial r}\left[(\frac{n-1}{2}\right){\left(\frac{\partial u_{300}}{\partial r}\right)}^{3}\\ +\left(\frac{\partial u_{301}}{\partial r}\right)] \end{array}$$

The associated boundary conditions $u_{321}\left(r_{1}\right)=u_{321}\left(r_{2}\right)=0$ .

We obtain very long solutions for the velocity and stream function, known as $u_{3}=\frac{1}{r}\frac{\partial \psi }{\partial r}$ , that mean $\psi =\int r\left(\left(u_{300}+\alpha u_{301}\right)+{W_{e}}^{2}\left(u_{320}+\alpha u_{321}\right)\right)\mathrm{dr}$ . The associated constants can be determined using the associated boundary conditions. Therefore, we will discuss these solutions graphically in the next section.

## 5. Solution analysis

Through the graphs of the fluid velocity function, we discussed and analysed the effect of changing temperature on the viscosity of a Carreau fluid and thus on its velocity through a hollow flexible channel. The program “MATHEMATICA 14” was used in this analysis. The following values were adopted to plot the fluid velocity function: $e_{1}=0.3$ , $e_{2}=0.7$ , $e_{3}=0.5$ , $e_{4}=0.5$ , $e_{5}=0.2$ , Ω=0.5 , $G_{1}=2$ , $G_{2}=1$ , $S_{1}=0.7$ , $S_{2}=0.3$ , $R_{n}=0.5$ , $P_{r}=1.7$ , ε=0.2 , φ=0.15 , $W_{e}=0.2$ , α=0.1 , n=0.3 .

The general shape of the fluid velocity function is a downward-concave curve where the maximum value of the curve is close to the catheter tube around the value of r=0.3 , also the ends of the curve are close to zero at the walls of the channel (the rigid inner and the flexible outer), which matches the boundary condition of the problem.

**Figure 1.**
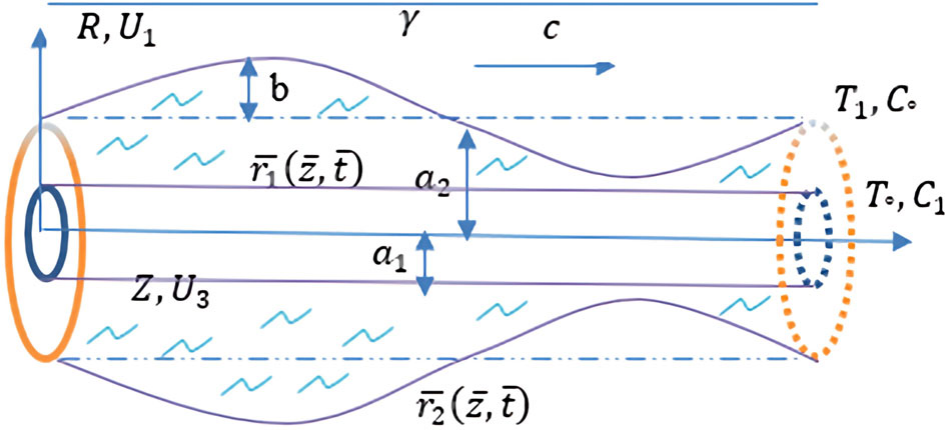
The problem ometry.

**Figure 2.**
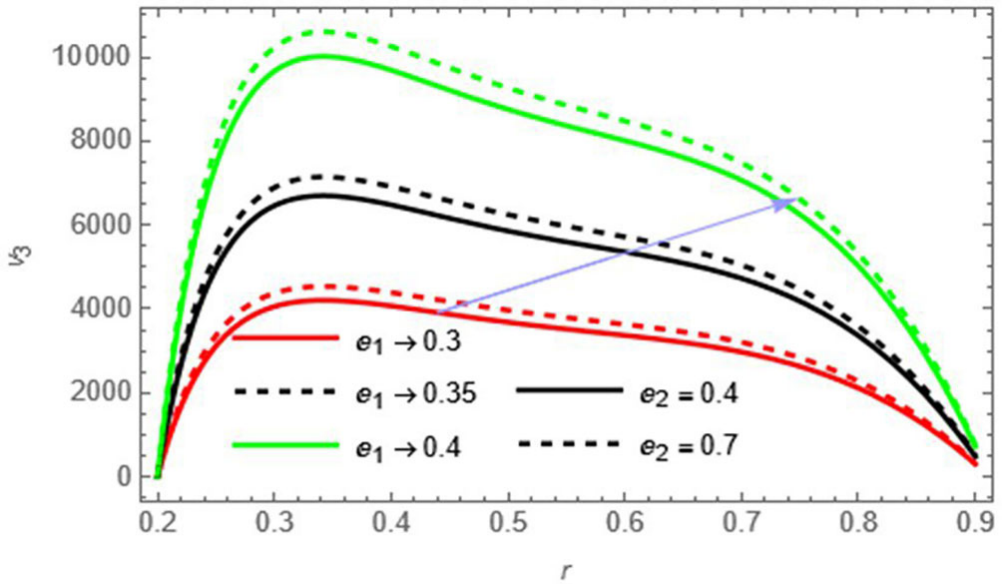
$\;e_{1}$ , $e_{2}$ Increasing these parameters enhances wall elasticity, which facilitates the fluid motion and increases the velocity.

**Figure 3.**
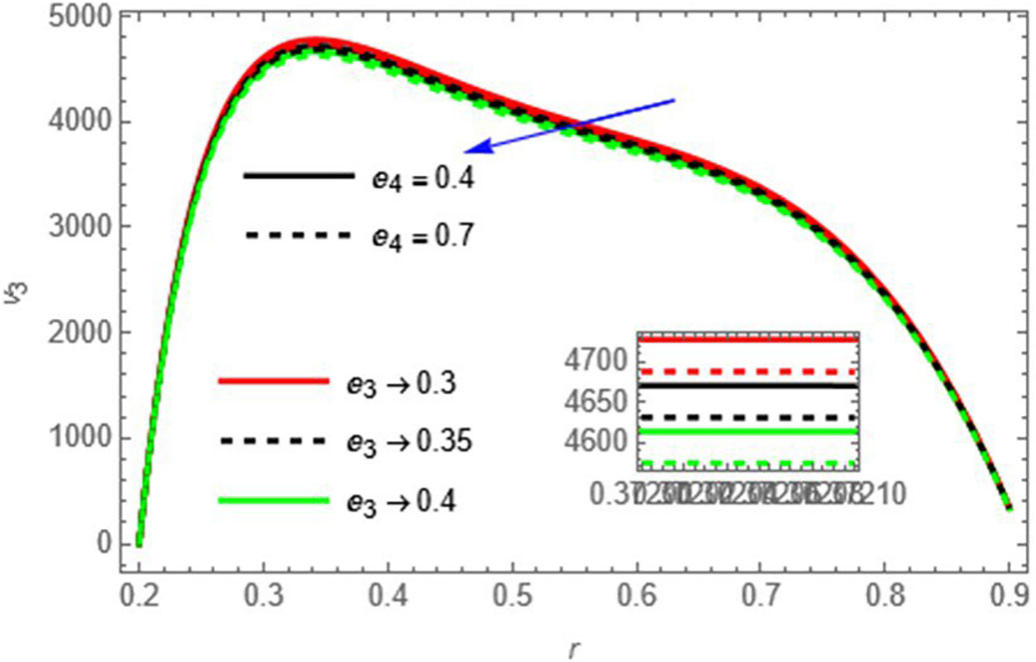
$\;e_{3}$ and $e_{4}$ These elasticity parameters resist fluid motion, reducing the velocity.

**Figure 4.**
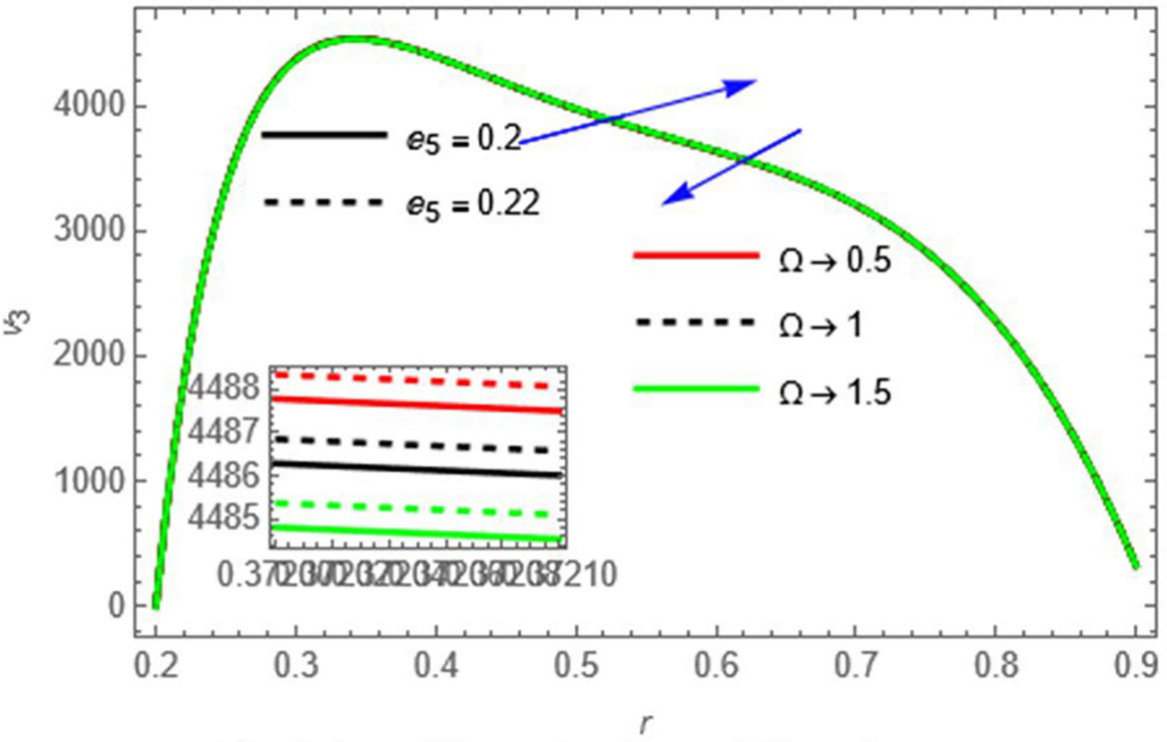
$e_{5}$ This parameter positively affects wall motion, resulting in higher fluid velocity, Ω Heat absorption or sink reduces thermal energy in the fluid, decreasing velocity.

**Figure 5.**
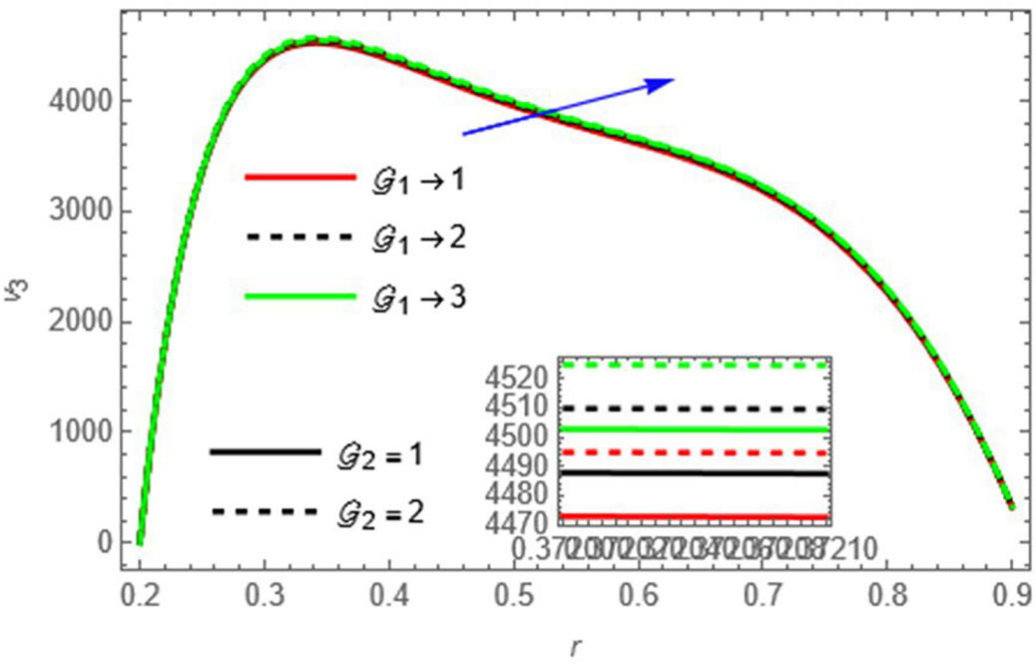
$\;G_{1}$ , $G_{2}$ Higher thermal and solutal Grashof numbers enhance buoyancy-driven flow, increasing the velocity.

**Figure 6.**
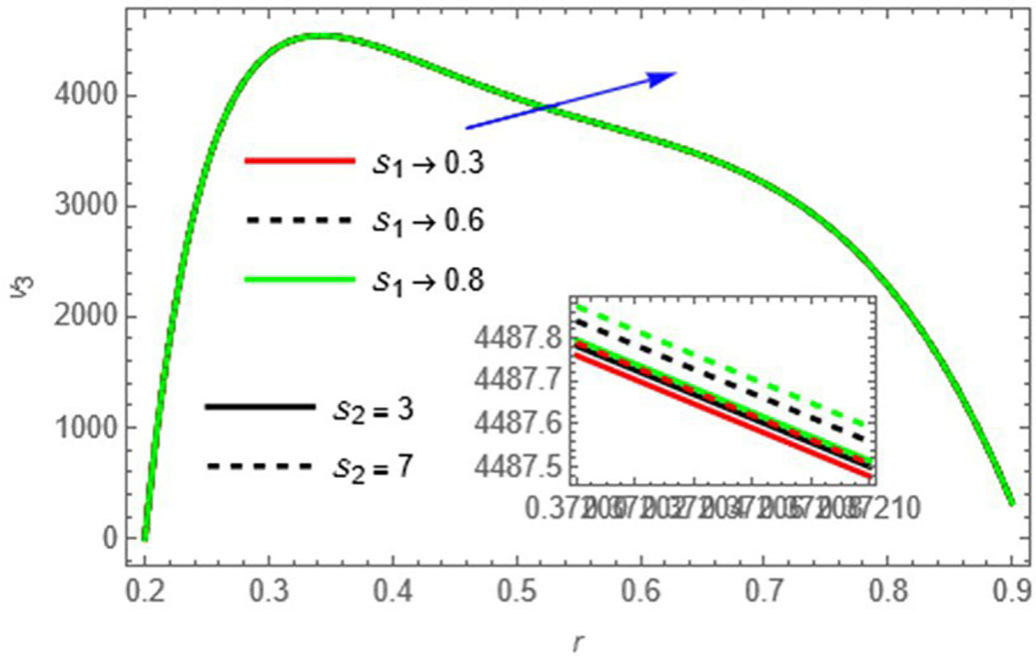
$S_{1}$ , and $S_{2}$ Increased Soret and Schmidt effects strengthen mass transfer-driven flow, boosting velocity.

**Figure 7.**
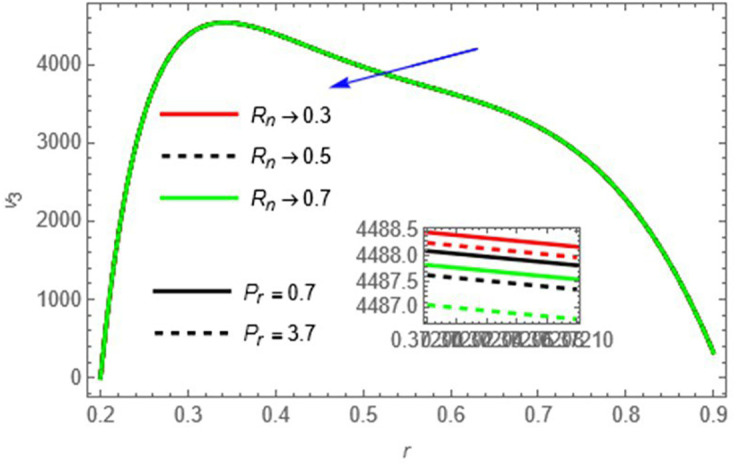
$\;R_{n}$ and $P_{r}$ Higher thermal radiation and Prandtl number reduce thermal diffusion and increase viscous effects, slowing down the fluid.

**Figure 8.**
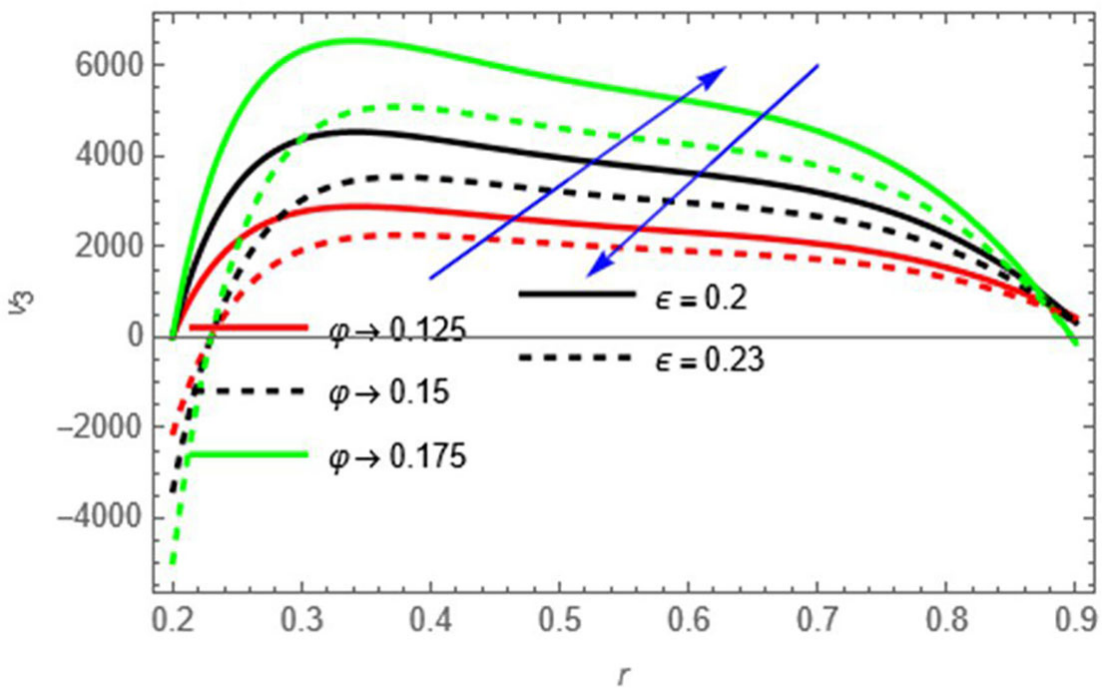
φ Larger wall wave amplitude enhances peristaltic pumping, increasing fluid velocity, ε increasing tube radius increases flow resistance near the walls, reducing velocity.

**Figure 9.**
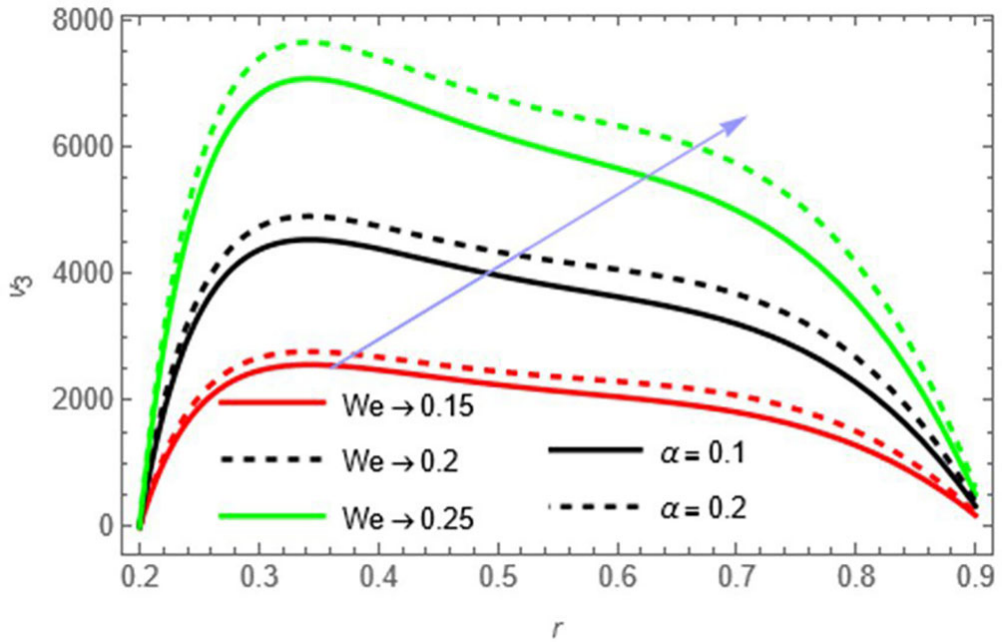
$W_{e}$ and α Higher Weissenberg number and perturbation parameter strengthen the fluid’s elastic and oscillatory response, increasing velocity.

**Figure 10.**
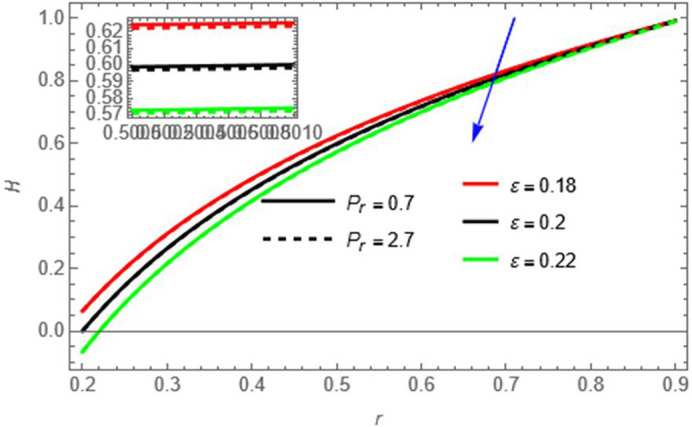
We notice that the fluid temperature decreases with increasing Prandtl number $P_{r}$ ​ and tube radius ratio ε . Higher $P_{r}$ ​ reduces thermal diffusivity, limiting heat penetration into the fluid, while larger ε, increases wall surface effects, enhancing heat transfer toward the walls and lowering the core temperature.

**Figure 11.**
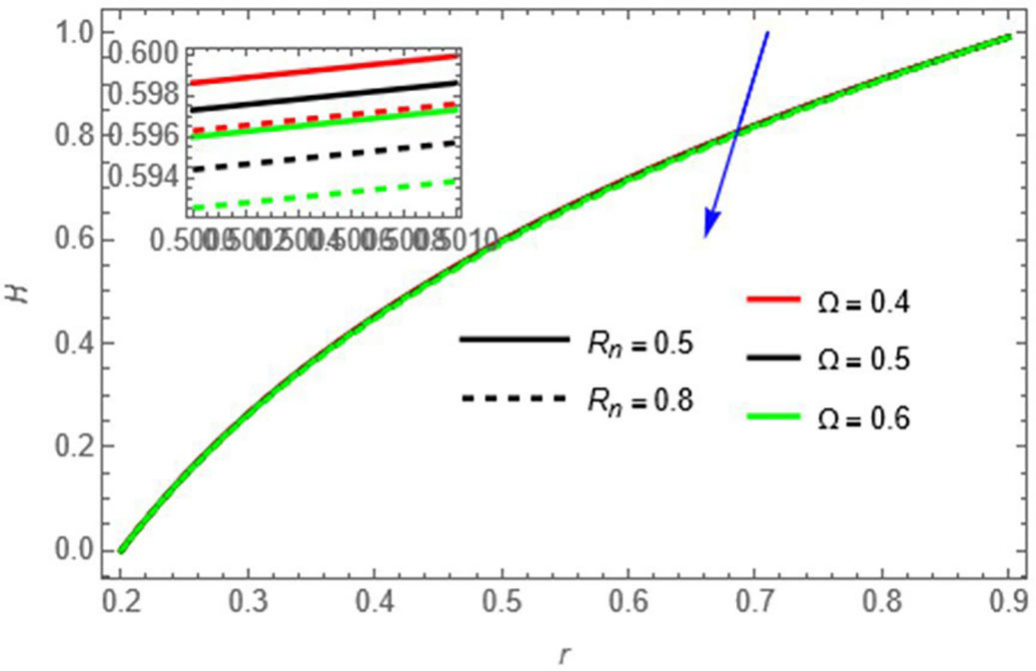
The fluid temperature decreases with increasing Ω and $R_{n}$ , as higher Ω, absorbs heat and larger $R_{n}$ ​ enhances radiative heat loss.

**Figure 12.**
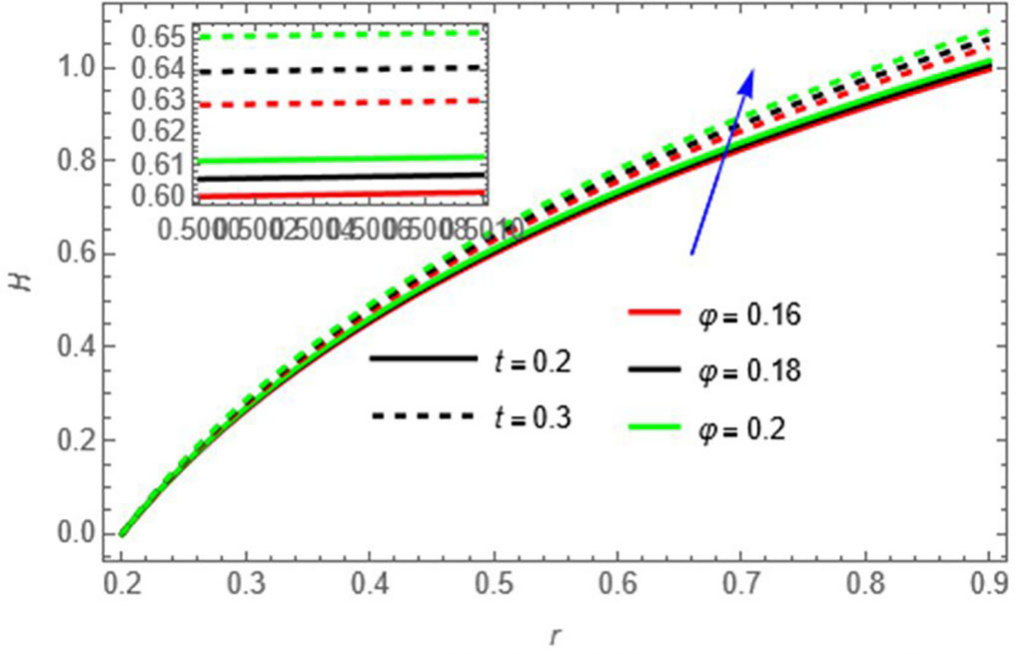
The fluid temperature increases with amplitude ratio φ and time t, as higher *φ* enhances wall-induced mixing and higher t, allows heat to diffuse and accumulate within the fluid.

**Figure 13.**
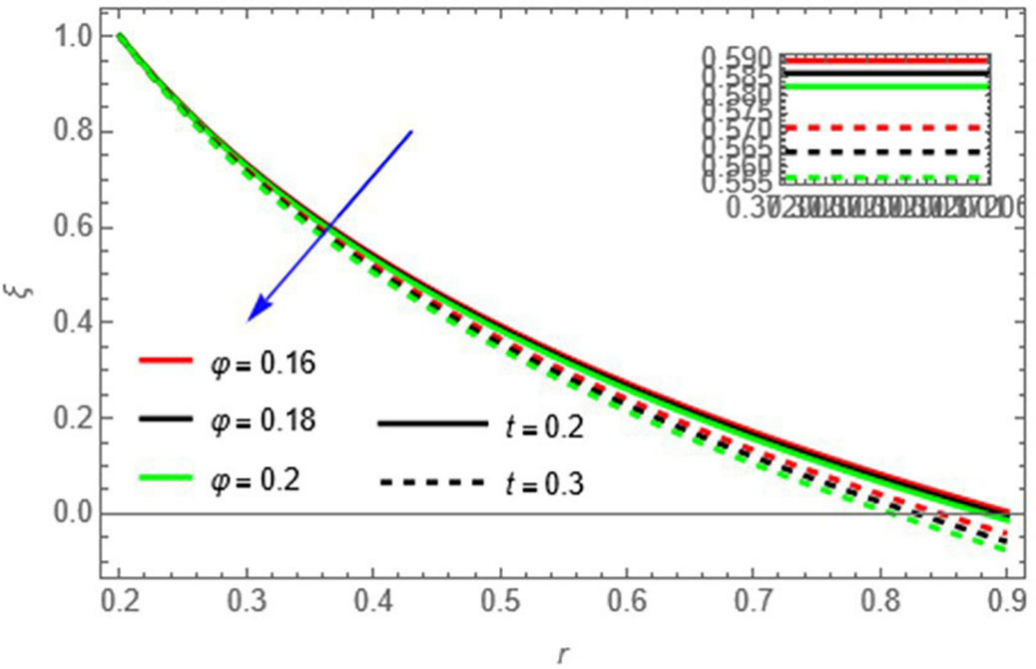
The fluid concentration decreases with increasing φ and t, as larger wall amplitude enhances mixing and longer time allows diffusion to reduce concentration locally.

**Figure 14.**
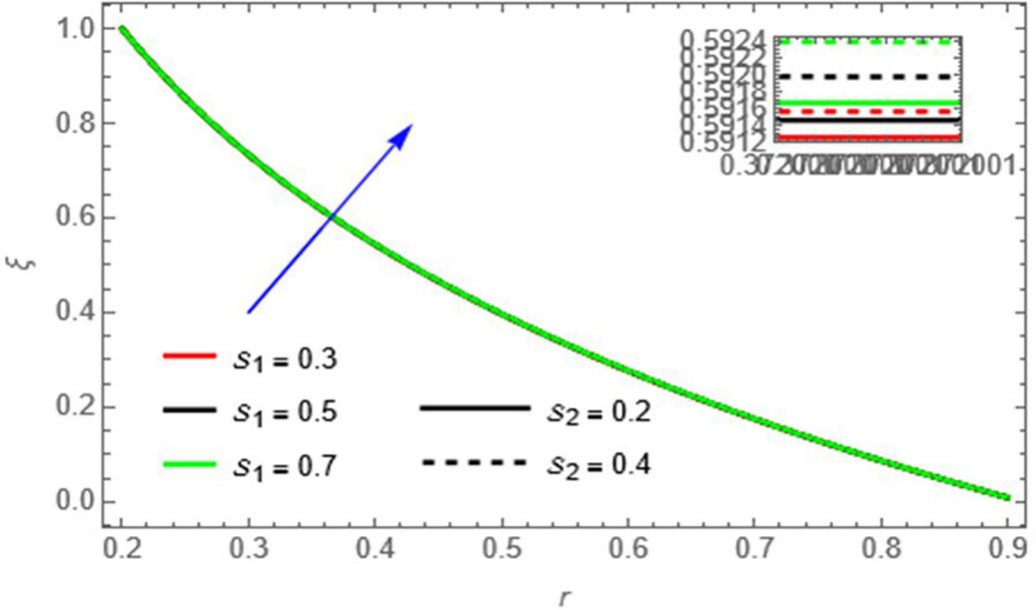
The fluid concentration increases with increasing $S_{1},\text{and}\;S_{2}$ ​, as higher Soret and Schmidt numbers strengthen mass transfer effects, promoting solute accumulation.

**Figure 15.**
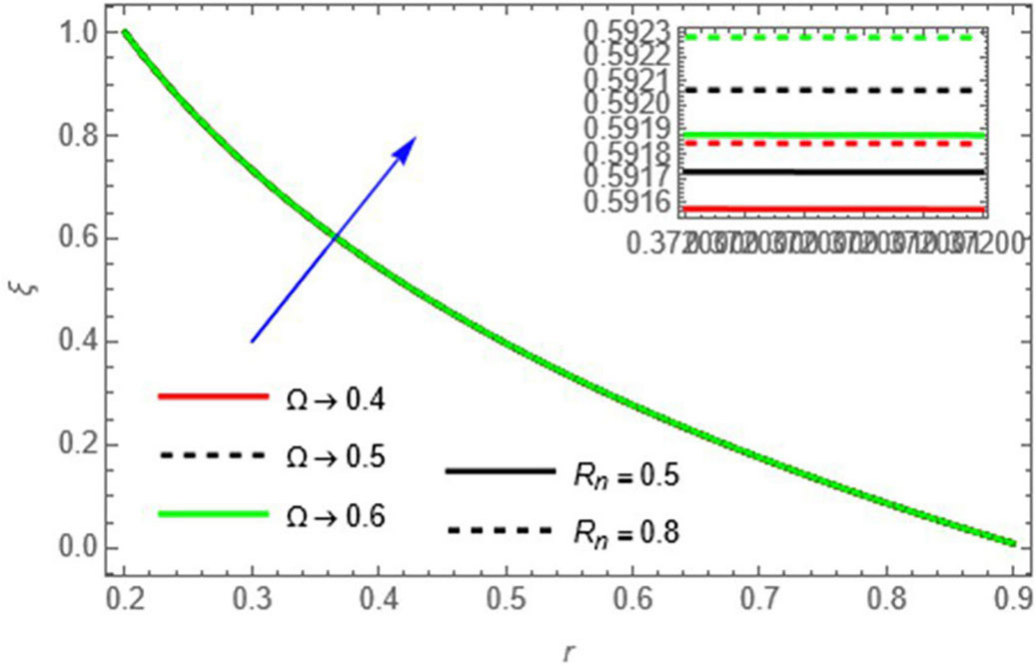
The fluid concentration increases with increasing $R_{n}$ and Ω, as higher thermal radiation and heat source parameters enhance energy transfer, supporting solute accumulation.

**Figure 16.**
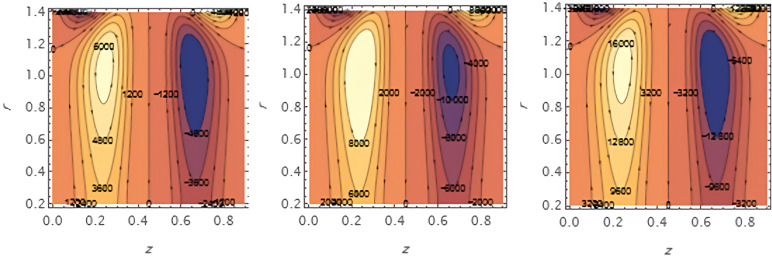
Increasing $e_{1}$ ​ enhances wall deformation, allowing larger boluses to form.

**Figure 17.**
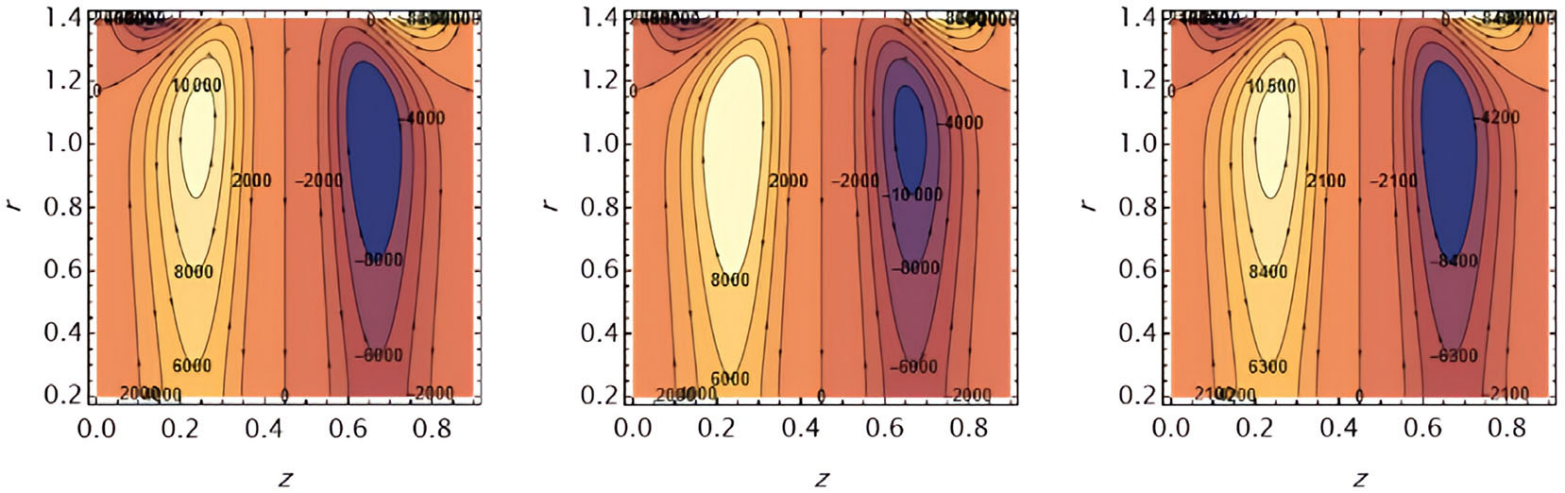
Increasing $e_{2}$ similarly increases wall motion, leading to larger trapped boluses.

**Figure 18.**
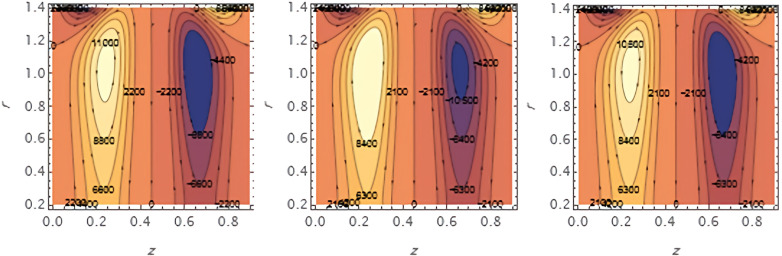
Increasing ​ $e_{3}$ resists wall motion, reducing the size of the trapped boluses.

**Figure 19.**
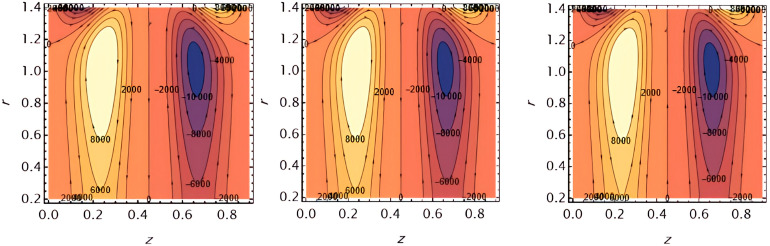
Higher solutal Grashof number $G_{2}$ ​ increases buoyancy effects, expanding the bolus size.

**Figure 20.**
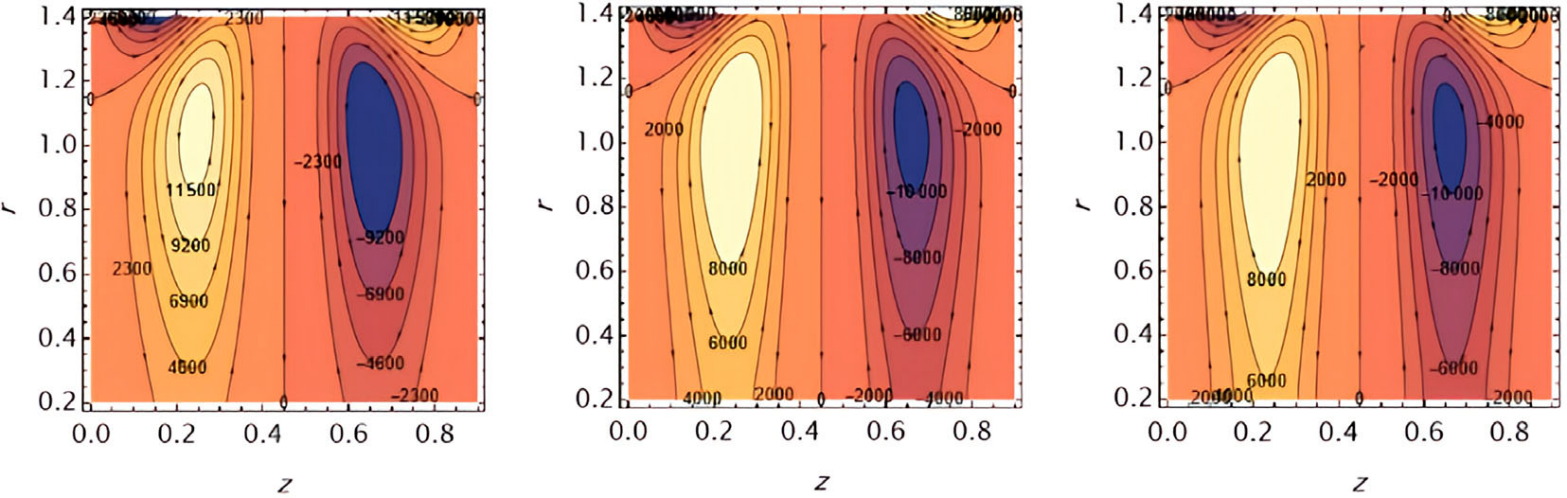
Increasing the catheter tube radius ε reduces flow resistance, allowing the boluses to expand.

**Figure 21.**
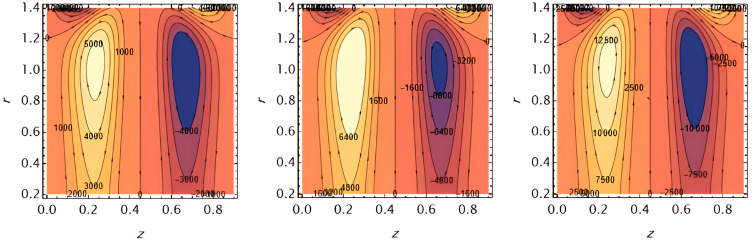
Larger wall wave φ amplitude enhances peristaltic pumping, increasing the trapped bolus size.

**Figure 22.**
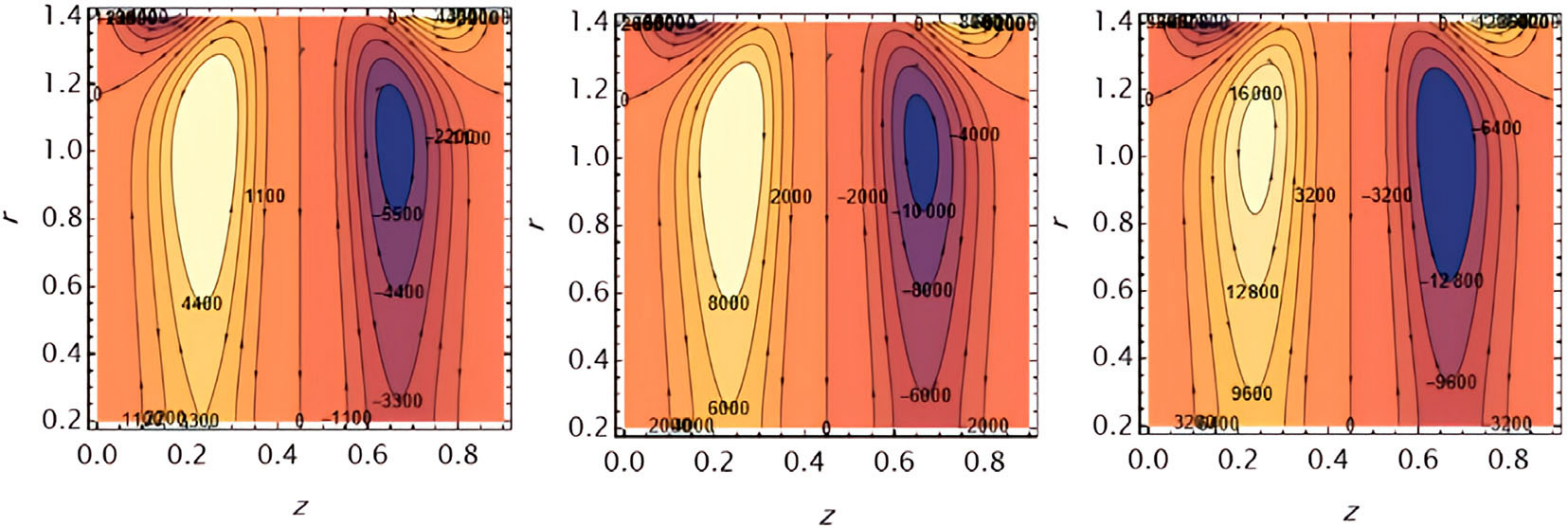
Higher Weissenberg number $\;W_{e}$ strengthens elastic effects, enlarging the boluses.

**Figure 23.**
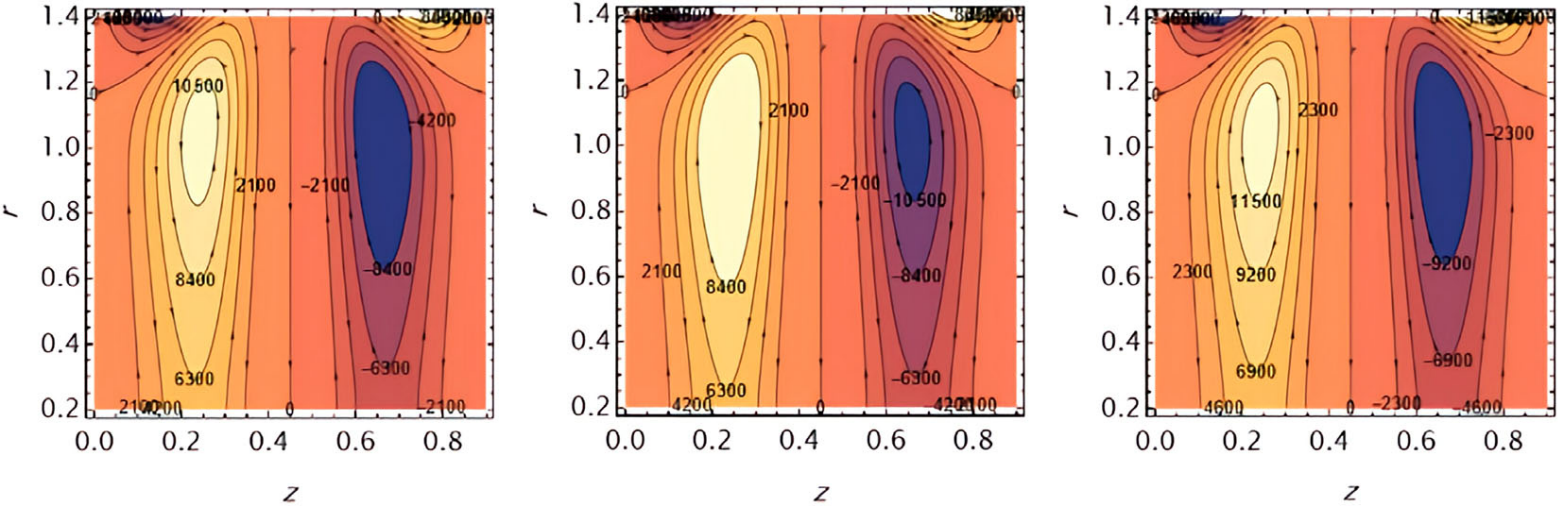
Increasing the perturbation parameter α enhances oscillatory motion, increasing bolus size.

## 6. Conclusions and Summary

Here we will go over the main points that affect the flow of an incompressible Carreau fluid via a flexible endoscopic hollow tube. Utilizing the perturbation method in conjunction with the MATHEMATICA-14 program, we ascertained the velocity function. We visually examined all the results that came from changing different relevant settings. The key points may be summarized as follows:

1- There is a positive correlation between the growth of $e_{1},e_{2},W_{e},\varphi ,e_{5},\alpha$ , $G_{1},G_{2},S_{1},\;\text{and}\;S_{2}$ while decreases the velocity is due to the increase in parameter $e_{3},e_{4}\;$ , $\;\varepsilon ,P_{r},R_{n}$ and Ω .
2- The trapped bolus expands with an increase $\;e_{1},e_{2},W_{e},\varphi ,\;\text{and}\;\varepsilon ,$ the trapped bolus shrinks increasing the values of $\alpha ,e_{3}.$

3- The following parameters $\;S_{1}$ , $S_{1}$ , $P_{r}$ , $R_{n}$ , Ω , $\;G_{1}$ , $\;e_{5}$ $,\;\text{and}\;e_{4}$ , have no effect on the stream function.

## Data Availability

All data underlying the results presented in this study are contained within the article. The figures were generated directly from analytical mathematical expressions, and no external datasets or numerical data were produced or used. Therefore, no datasets requiring deposition in a public repository are associated with this work.
