# Supplementary material for: Analysis of the peristaltic flow of a variable viscosity Carreau fluid affected by temperature and concentration through an endoscope hollow flexible channel
Source: F1000Res. 2026 Mar 2;15:18. Originally published 2026 Jan 7. [Version 2] doi: 10.12688/f1000research.172584.2 (PMC12976611; doi:10.12688/f1000research.172584.2)
Supplement: Peer review history

# Reviewer report: Reviewer response for version 2

**DOI:** 10.5256/f1000research.196535.r463588

**Published:** 2026-03-10

**Authors:** Shekar Marudappa

**Affiliations:** 1 B. M. S. College of Engineering, Bengaluru, Karnataka, India

No further comments.

Is the work clearly and accurately presented and does it cite the current literature?

Partly

If applicable, is the statistical analysis and its interpretation appropriate?

Partly

Are all the source data underlying the results available to ensure full reproducibility?

Partly

Is the study design appropriate and is the work technically sound?

Partly

Are the conclusions drawn adequately supported by the results?

Partly

Are sufficient details of methods and analysis provided to allow replication by others?

Partly

Reviewer Expertise:

Convective flow through porous medium. Biological fluid flow.

I confirm that I have read this submission and believe that I have an appropriate level of expertise to confirm that it is of an acceptable scientific standard.

# Reviewer report: Reviewer response for version 2

**DOI:** 10.5256/f1000research.196535.r463590

**Published:** 2026-03-04

**Authors:** Ahmed Gamal

**Affiliations:** 1 Menoufia University, Shebin El-Kom, Egypt

Dear Authors,

 Thank you for submitting the revised version of your manuscript and for providing detailed responses to the previous review comments. The manuscript shows improvements in structure, expanded literature coverage, and clearer figure presentation.

 However, after careful re-evaluation, it is evident that the core scientific concerns raised in the initial review have not been substantively resolved. While textual modifications were made, the fundamental issues related to validation, novelty, methodological transparency, and physical interpretation remain insufficiently addressed.

 At this stage, the manuscript does not yet meet the scientific soundness standards required for publication. The following major issues must be comprehensively resolved:

 1. Absence of Explicit and Verifiable Validation (Critical)

**Problem:**

 Although you stated that a validation section was added, the manuscript does not provide a clear, checkable validation of the model. No quantitative comparison with a known analytical solution, limiting case, or previously published benchmark is shown.

**Required Action:**

 You must include a dedicated “Validation” section demonstrating that your formulation reduces correctly to established results. For example:

- Newtonian limit: We=0We = 0We=0, n=1n = 1n=1
- Constant viscosity: α=0\alpha = 0α=0
- No buoyancy: G1=G2=0G_1 = G_2 = 0G1​=G2​=0
- Rigid wall limit

 

- Explicit reference to the benchmark study,
- Graphical overlap or tabulated comparison,
- Quantitative error (if applicable).

Without this, the correctness of your analytical development cannot be verified.

 2. Novelty Is Not Scientifically Demonstrated (Critical)

**Problem:**

 The manuscript claims novelty based on combining Carreau fluid, variable viscosity, wall flexibility, and catheter geometry. However, similar configurations already exist in the literature (e.g., Akram & Akbar, 2023). The manuscript does not clearly articulate what new physical insight arises specifically from adding wall flexibility to the existing framework.

**Required Action:**

  

- Add a structured comparison table in the Introduction (columns: geometry, wall flexibility, viscosity model, temperature effect, concentration effect, catheter inclusion, solution method).
- Explicitly state what is new and what physical mechanism is uncovered.
- In the Results section, quantitatively demonstrate how wall flexibility modifies the behavior compared to the rigid-wall case.

Novelty must be demonstrated analytically or physically—not simply stated.

 3. Physical Interpretation Remains Superficial

**Problem:**

 The Results section primarily lists parameter trends (e.g., “increasing G1 increases velocity”) without explaining the governing physical mechanism.

**Required Action:**

 Rewrite the Results section to explain *why* each parameter produces the observed effect. For example:

- Explain buoyancy-viscosity competition for G1G_1G1​.
- Explain shear-thinning–elastic interaction for nnn and WeWeWe.
- Explain how flexibility alters pressure-gradient coupling.

Each major parameter group should include a mechanistic explanation, not only a directional trend.

 4. Insufficient Mathematical Transparency and Reproducibility

**Problem:**

 The derivations omit key intermediate steps. The constants B1,B2,B3,B4B_1, B_2, B_3, B_4B1​,B2​,B3​,B4​ are presented without showing how boundary conditions determine them. The final explicit expressions for u300,u301,u310,u311u_{300}, u_{301}, u_{310}, u_{311}u300​,u301​,u310​,u311​ and the stream function ψ\psiψ are not provided.

 The solution process currently functions as a black box.

**Required Action:**

  

- Either a detailed Appendix containing the final closed-form expressions,
- Or complete supplementary material (e.g., Mathematica notebook) generating all figures,
- A clear explanation of how integration constants are obtained.

Reproducibility is mandatory for analytical research.

 5. Parameter Justification Is Inadequate

**Problem:**

 The selected parameter values (especially wall elasticity parameters ℛ1–ℛ5 and buoyancy parameters G1,G2G_1, G_2G1​,G2​) are described as “physically realistic” without traceable citations.

**Required Action:**

 For each major parameter:

- Provide a reference supporting the numerical range,
- Clarify whether values correspond to physiological systems or are dimensionless theoretical choices,
- Include a short physical explanation.

6. Validity of Approximations Is Not Explicitly Quantified

**Problem:**

 The assumptions δ≪1\delta \ll 1δ≪1, low Reynolds number, and double perturbation in WeWeWe and α\alphaα are stated but not quantitatively justified.

**Required Action:**

  

- State explicit bounds (e.g., We<0.3We < 0.3We<0.3, α<0.2\alpha < 0.2α<0.2, δ<0.1\delta < 0.1δ<0.1),
- Confirm that plotted parameter values satisfy these bounds,
- Briefly discuss the expected error order of truncation.

7. Governing Equation Referencing Must Be Complete

 Each physical model component must have a direct citation:

- Carreau constitutive equation form used,
- Reynolds viscosity approximation,
- Radiation model formulation,
- Soret and diffusion terms,
- Elastic wall operator.

No equation should appear without traceable sourcing.

 8. Notational Consistency and Technical Accuracy

 Several symbols appear duplicated or inconsistently defined. Please:

- Ensure complete consistency between Nomenclature and equations,
- Verify all dimensionless group definitions,
- Eliminate typographical inconsistencies.

Final Assessment

 The manuscript requires substantial scientific strengthening rather than additional editorial refinement. Validation, reproducibility, quantified approximation limits, and demonstrated novelty are mandatory.

 Without these major revisions, the manuscript cannot be considered scientifically sound.

Is the work clearly and accurately presented and does it cite the current literature?

No

If applicable, is the statistical analysis and its interpretation appropriate?

Not applicable

Are all the source data underlying the results available to ensure full reproducibility?

No

Is the study design appropriate and is the work technically sound?

Partly

Are the conclusions drawn adequately supported by the results?

No

Are sufficient details of methods and analysis provided to allow replication by others?

Partly

Reviewer Expertise:

Mechanical Engineering and Mathematical Engineering– with specific focus on :Peristaltic flow of complex/non-Newtonian fluids (e.g., Carreau and couple-stress fluids, )Heat and mass transfer in multi-physics systemsMagnetohydrodynamics (MHD) and porous media flowsNanofluids and hybrid nanofluidsAnalytical and numerical modeling techniques (e.g., FEM, perturbation methods)

I confirm that I have read this submission and believe that I have an appropriate level of expertise to state that I do not consider it to be of an acceptable scientific standard, for reasons outlined above.

# Reviewer report: Reviewer response for version 1

**DOI:** 10.5256/f1000research.190322.r448707

**Published:** 2026-01-23

**Authors:** Shekar Marudappa

**Affiliations:** 1 B. M. S. College of Engineering, Bengaluru, Karnataka, India

The research article titled **"Analysis of the peristaltic flow of a variable viscosity Carreau fluid affected by temperature and concentration through an endoscope hollow flexible channel”.** In its current form the paper is **"not recommended for indexing"**.

 The following review details:

**Major Concerns**

 • **Lack of Novelty and Originality:** It is noticed that the study does not demonstrate sufficient originality relative to existing literature. The introduction is described as brief and descriptive, failing to identify genuine research gaps or critically analyze prior studies.

 • **Methodological Rigor and Transparency:** Key mathematical steps, particularly in the velocity function section (Section 4.3), are either omitted or inadequately explained. Furthermore, multiple mathematical approximations are used without defining their **ranges of validity or error bounds**, which compromises the reliability of the analysis.

 • **Absence of Validation:** The manuscript lacks any form of benchmarking. The results are not compared against known analytical solutions, limiting cases, or previously published studies to confirm their accuracy.

 • **Insufficient Physical Interpretation:** The reviewer points out that the results are presented merely as parameter trends (e.g., velocity increasing or decreasing) without providing a **clear physical explanation** for these behaviors. Consequently, the conclusions are not seen as providing meaningful scientific insight.

 • **Reproducibility Issues:** The numerical values used for the graphical analysis are not justified or referenced. While the authors claim all data is contained within the article, the reviewer argues that no explicit analytical expressions or datasets are provided to allow for the independent reproduction of the figures.

**Minor Concerns**

 • **Clarity and Language Quality:** The reviewer suggests that the manuscript requires improvements in **English language quality**, structure, and overall clarity.

 • **Literature Coverage:** The article fails to adequately engage with or cite recent developments in the field of peristaltic transport of non-Newtonian fluids.

 • **Parameter Justification:** While the authors list values for parameters like flexural stiffness ( *χ*1​) and longitudinal tension ( *χ*2​) in the solution analysis, there is no explanation provided in the text for why these specific values were chosen.

Is the work clearly and accurately presented and does it cite the current literature?

Partly

If applicable, is the statistical analysis and its interpretation appropriate?

Partly

Are all the source data underlying the results available to ensure full reproducibility?

Partly

Is the study design appropriate and is the work technically sound?

Partly

Are the conclusions drawn adequately supported by the results?

Partly

Are sufficient details of methods and analysis provided to allow replication by others?

Partly

Reviewer Expertise:

Convective flow through porous medium. Biological fluid flow.

I confirm that I have read this submission and believe that I have an appropriate level of expertise to state that I do not consider it to be of an acceptable scientific standard, for reasons outlined above.

# Response

**Published:** 2026-02-04

**Authors:** Salwa Al-Tamimi

**Affiliations:** Not Applicable, Al Quadisiya, Iraq

We sincerely thank the reviewer for the detailed and constructive comments. We greatly appreciate the time and effort taken to provide feedback, which has significantly helped improve the clarity, scientific rigor, and overall quality of the manuscript. Below, we provide point-by-point responses to all major and minor concerns:

**Major Concerns**

**Comment 1: Lack of Novelty and Originality**

*Concern:* The study does not demonstrate sufficient originality relative to existing literature. The introduction is brief and descriptive, failing to identify genuine research gaps or critically analyze prior studies.

**Response:**

 We have substantially revised the Introduction to clearly highlight the novelty of the present study. The revised section now identifies specific gaps in the current literature on peristaltic flow of non-Newtonian fluids with variable viscosity in flexible endoscope channels. We have also critically analyzed prior studies and explicitly stated the unique contributions of this work, including the combined effects of temperature, concentration, and variable viscosity on peristaltic transport

**Comment 2: Methodological Rigor and Transparency**

*Concern:* Key mathematical steps, especially in Section 4.3 (velocity function), are omitted or inadequately explained. Multiple approximations are used without specifying their validity ranges or error bounds.

**Response:**

 All mathematical derivations in Section 4.3 have been expanded to include detailed intermediate steps. Each approximation used in the analysis is now clearly justified, and its range of validity and expected error bounds are explicitly stated. This ensures transparency and enhances the reliability of the results.

**Comment 3: Absence of Validation**

*Concern:* The manuscript lacks benchmarking against analytical solutions, limiting cases, or previously published studies.

**Response:**

 A new validation section has been added. The results have been compared with known limiting cases and available published studies on peristaltic flow of non-Newtonian fluids. The comparisons show good agreement, confirming the accuracy and reliability of the proposed model

**Comment 4: Insufficient Physical Interpretation**

*Concern:* Results are presented only as trends without clear physical explanations.

**Response:**

 The Results and Discussion section has been revised to include detailed physical interpretations of all observed trends. For each figure, we explain the underlying mechanisms influencing velocity, pressure, and concentration profiles, providing meaningful scientific insights.

**Comment 5: Reproducibility Issues**

*Concern:* Numerical values are not justified or referenced, and analytical expressions or datasets are not provided for independent reproduction.

**Response:**

 All parameter values used in the graphical analysis are now justified with references from previous studies. Additionally, the full analytical expressions and required datasets have been provided in the manuscript and supplementary material, ensuring that all results can be independently reproduced.

**Minor Concerns**

**Comment 6: Clarity and Language Quality**

**Response:**

 The manuscript has undergone careful proofreading and editing to improve English language quality, clarity, and overall structure.

**Comment 7: Literature Coverage**

**Response:**

 The Introduction and literature review have been expanded to incorporate recent and relevant studies on peristaltic transport of non-Newtonian fluids. This provides a more comprehensive background and situates the current work within the existing research context.

**Comment 8: Parameter Justification**

**Response:**

 All parameters used, including flexural stiffness (χ1) and longitudinal tension (χ2), are now justified in the text with references to relevant literature, and their physical significance is discussed

 We thank the reviewer again for the constructive feedback and hope that the revised manuscript now meets the standards for indexing and publication.

# Reviewer report: Reviewer response for version 1

**DOI:** 10.5256/f1000research.190322.r448710

**Published:** 2026-01-23

**Authors:** P. Lakshminarayana Lakshminarayana

**Affiliations:** 1 Vellore Institute of Technology, Vellore, Tamil Nadu, India

This study investigates the peristaltic flow of a variable-viscosity Carreau fluid through a hollow, flexible endoscopic channel under the combined effects of temperature and concentration variations. The analysis highlights the influence of non-Newtonian behaviour on the channel's transport characteristics.

 I have outlined some constructive feedback and recommendations to improve the manuscript below.

1.
  1. Use directional arrows in the figures to clearly illustrate the increasing or decreasing trends of the plotted parameters.
  2. Include a detailed analysis of heat transfer characteristics, particularly the Nusselt number, to enhance the thermal performance discussion.
  3. Some equations are missing equation numbers. Please ensure that all equations are properly numbered and referenced in the text.
  4. Provide appropriate references for the governing equations and the applied boundary conditions.
  5. Include a validation of the present model, preferably through comparison with available analytical, numerical, or previously published results.
  6. Add a comprehensive Nomenclature section, clearly defining all symbols along with their corresponding SI units.
  7. Include a subsection discussing the limitations of the present study and outlining possible future research directions.
  8. Carefully recheck all governing equations for mathematical accuracy and consistency.
  9. The author (s) should give a proper discussion for every figure presented in the results and discussion section, along with the physical mechanism. Please see the refs:  Thermal performance and MHD peristaltic flow of hybrid nanofluid ( *Au-Ta*/Blood) in an asymmetric conduit with electro-osmosis and shape factor effects; Unveiling the thermally dissipative peristaltic pumping characteristics of hydromagnetic nanofluid in an oblique micro-wavy conduit via artificial neural network-based optimization; Investigation of convective peristaltic flow of non-Newtonian fluids through a non-uniform tapered porous conduit with Ohmic heating and viscous dissipation.
  10. In recent years, similar types of work have been investigated by different authors. Therefore, the original purpose of the present efforts and the new findings in this study are not fully described. Please justify.
  11. Perform a thorough review to correct grammatical, spelling, and typographical errors throughout the manuscript.
  12. Clearly highlight the novelty of the study by explicitly stating what distinguishes this work from existing literature at the end of the Introduction.
  13. The literature survey presented in the Introduction is limited. It is recommended to strengthen it by incorporating relevant and recent studies related to magnetohydrodynamics (MHD), peristaltic transport, and perturbation techniques, using the suggested references: Electroosmotic Effects on Peristaltic Transport of Ree-Eyring Nanofluid with Double Diffusive Convection in Symmetric Microchannel; A study on bioconvective peristaltic flow of a Casson nanofluid in a porous uniform/non‐uniform flexible conduit; Investigation of a conducting Casson fluid flow through a porous flexible microfluidic channel with catalytic effects: application in pharmaceutical fluid processing; Convective peristaltic pumping of MHD Ree-Eyring nanofluid in a chemically reacting flexible divergent channel with activation energy and radiation.
  14. Check the correctness of all mathematical equations present in the manuscript. Especially, the non-dimensional parameters are displayed in this manuscript. Is this all dimensionally true?
  15. The author(s) didn’t provide the validation of the present results with existing studies. Include it in the revised version of the manuscript.

Is the work clearly and accurately presented and does it cite the current literature?

Partly

If applicable, is the statistical analysis and its interpretation appropriate?

Not applicable

Are all the source data underlying the results available to ensure full reproducibility?

Yes

Is the study design appropriate and is the work technically sound?

Yes

Are the conclusions drawn adequately supported by the results?

Yes

Are sufficient details of methods and analysis provided to allow replication by others?

Partly

Reviewer Expertise:

Heat transfer, Fluid mechanics, Peristaltic Transport

I confirm that I have read this submission and believe that I have an appropriate level of expertise to confirm that it is of an acceptable scientific standard, however I have significant reservations, as outlined above.

# Response

**Published:** 2026-02-04

**Authors:** Salwa Al-Tamimi

**Affiliations:** Not Applicable, Al Quadisiya, Iraq

We sincerely thank the reviewer for the detailed and constructive comments, which have significantly helped improve the scientific quality, clarity, and presentation of the manuscript. All points raised have been carefully addressed in the revised version. Detailed responses are provided below:

**Comment 1:**

 Use directional arrows in the figures to clearly illustrate the increasing or decreasing trends of the plotted parameters.

**Response:**

 Directional arrows have been added to all figures where appropriate to clearly indicate increasing or decreasing trends of the plotted parameters, enhancing the clarity and readability of the results.

**Comment 2:**

 Include a detailed analysis of heat transfer characteristics, particularly the Nusselt number, to enhance the thermal performance discussion.

**Response:**

 A detailed analysis of heat transfer characteristics.

**Comment 3:**

 Some equations are missing equation numbers. Please ensure that all equations are properly numbered and referenced in the text.

**Response:**

 All equations in the manuscript have now been carefully numbered and appropriately referenced in the text to ensure consistency and ease of cross-referencing.

**Comment 4:**

 Provide appropriate references for the governing equations and the applied boundary conditions.

**Response:**

 All governing equations and boundary conditions have been reviewed and supported with proper references to the relevant literature, ensuring full scientific documentation and credibility.

**Comment 5:**

 Include a validation of the present model, preferably through comparison with available analytical, numerical, or previously published results.

**Response:**

 A validation section has been added, where selected cases are compared with published results from similar studies. The comparisons demonstrate good agreement, confirming the accuracy and reliability of the proposed model.

**Comment 6:**

 Add a comprehensive Nomenclature section, clearly defining all symbols along with their corresponding SI units.

**Response:**

 A comprehensive Nomenclature section has been included, listing all symbols used in the manuscript along with their definitions and corresponding SI units for clarity and transparency.

**Comment 7:**

 Include a subsection discussing the limitations of the present study and outlining possible future research directions.

**Response:**

 A subsection on the limitations of the current study and potential directions for future research has been added at the end of the discussion section to acknowledge constraints and suggest further investigation.

**Comment 8:**

 Carefully recheck all governing equations for mathematical accuracy and consistency.

**Response:**

 All governing equations have been thoroughly rechecked for mathematical accuracy and consistency. Any discrepancies have been corrected to ensure full reliability of the results.

**Comment 9:**

 The authors should provide a proper discussion for every figure presented in the results and discussion section, along with the physical mechanism.

**Response:**

 Detailed discussions have been added for each figure in the Results and Discussion section, including physical interpretations and underlying mechanisms, following relevant references to similar studies for comparison.

**Comment 10:**

 The originality and new findings of the study are not fully described. Please justify.

**Response:**

 The Introduction and Results sections have been revised to clearly highlight the novelty of the study and explicitly state what distinguishes this work from existing literature, including the unique modeling approach and new physical insights.

**Comment 11:**

 Perform a thorough review to correct grammatical, spelling, and typographical errors throughout the manuscript.

**Response:**

 The manuscript has undergone a thorough review and all grammatical, spelling, and typographical errors have been corrected to improve clarity and readability.

**Comment 12:**

 The literature survey presented in the Introduction is limited. Strengthen it by incorporating relevant and recent studies.

**Response:**

 The literature review in the Introduction has been expanded to include recent studies on magnetohydrodynamics (MHD), peristaltic transport, and perturbation techniques, including the suggested references. This provides a more comprehensive background and places the current study in context with existing research.

**Comment 13:**

 Check the correctness of all mathematical equations, especially non-dimensional parameters. Are they dimensionally consistent?

**Response:**

 All mathematical equations, including the non-dimensional parameters, have been carefully checked for dimensional consistency and corrected where necessary.

**Comment 14:**

 The authors did not provide validation of the present results with existing studies. Include it in the revised manuscript.

**Response:**

 As mentioned earlier, a validation section has been added, including comparisons with previously published analytical and numerical results, which confirms the accuracy and robustness of the present findings

# Reviewer report: Reviewer response for version 1

**DOI:** 10.5256/f1000research.190322.r448711

**Published:** 2026-01-22

**Authors:** Anum Tanveer

**Affiliations:** 1 Mirpur University of Science and Technology (MUST), Mirpur, Pakistan

**Review Report**

 The abstract presents a well-structured and coherent study on the peristaltic flow of a variable-viscosity Carreau fluid in a flexible, axisymmetric channel with biomedical relevance. The background clearly establishes the importance of peristaltic transport in medical and industrial applications, particularly in endoscopic systems. The methodology is appropriately described, highlighting the use of realistic assumptions such as long wavelength and low Reynolds number, along with a suitable analytical approach using the uniform perturbation method. The results are concisely summarized and indicate meaningful physical insights into the effects of temperature and concentration on fluid velocity and pressure gradient. The conclusions align well with the results and emphasize the practical implications of the study. Overall, the manuscript is clear, technically sound, and contributes positively to the understanding of non-Newtonian peristaltic flow in biomedical contexts. However I have few minor points to address before formal acceptance

1. Geometry of figure is wrongly listed as “problem ometry”.
2. Commas and fullstops are missing after each equation
3. Section 3 and 4 seems similar in heading. Section 3 should be revised as simplified equations instead of solution method. Section 4 can be listed as Solution Method.
4. Introduction is very limited and so are the references. The number of effects added should be addressed in Introduction and so the relevant references can also be added.
5. Reference list is too short, some recent selections on the topic can be included

1. Peristaltic rotating motion of couple stress nanofluid affected by Soret and Dufour effects: An application to nanotechnology, ZAMM - Zeitschrift fuer Angewandte Mathematik und Mechanik, 105(5), e70047, (2025)
2. Flow and heat transfer characteristics in fallopian tube with metachronal wave of cilia, Journal of Mechanics, 2023, 39, 385–394
3. Dynamic interactions in MHD Jeffrey fluid: Exploring peristalsis, electro osmosis and homogeneous/heterogeneous chemical reactions, Alexandria Engineering Journal 94 (2024) 354–365
4. Enhancement in heat generation through ternary hybrid nanofluid in a periodic channel, Case Studies in Thermal Engineering, 69, 106011, (2025).
5. Numerical analysis of ciliated transport of fluid in human organs, Physics of Fluids 37 (2025) doi.org/10.1063/5.0307957.

Is the work clearly and accurately presented and does it cite the current literature?

Yes

If applicable, is the statistical analysis and its interpretation appropriate?

Yes

Are all the source data underlying the results available to ensure full reproducibility?

Yes

Is the study design appropriate and is the work technically sound?

Yes

Are the conclusions drawn adequately supported by the results?

Yes

Are sufficient details of methods and analysis provided to allow replication by others?

Yes

Reviewer Expertise:

Fluid Mechanics

I confirm that I have read this submission and believe that I have an appropriate level of expertise to confirm that it is of an acceptable scientific standard, however I have significant reservations, as outlined above.

# Response

**Published:** 2026-02-04

**Authors:** Salwa Al-Tamimi

**Affiliations:** Not Applicable, Al Quadisiya, Iraq

We sincerely thank the reviewer for the constructive comments and valuable suggestions, which have significantly helped us to improve the quality and clarity of the manuscript. All comments have been carefully addressed, and the manuscript has been revised accordingly. Our detailed responses are provided below.

**Comment 1:**

 Geometry of figure is wrongly listed as “problem ometry”.

**Response:**

 Thank you for pointing out this typographical error. The geometry description of the figure has been corrected, and the incorrect term “problem ometry” has been replaced with the appropriate wording in the revised manuscript.

**Comment 2:**

 Commas and fullstops are missing after each equation.

**Response:**

 We agree with the reviewer. Proper punctuation has now been added after all equations throughout the manuscript to ensure consistency and improve readability.

**Comment 3:**

 Section 3 and 4 seems similar in heading. Section 3 should be revised as simplified equations instead of solution method. Section 4 can be listed as Solution Method.

**Response:**

 We appreciate this insightful comment. Following the reviewer’s suggestion, the manuscript structure has been revised. Section 3 has been reformulated to focus on the derivation and presentation of the simplified governing equations, while Section 4 has been clearly redefined and retitled as “Solution Method” to avoid overlap and improve clarity.

**Comment 4:**

 Introduction is very limited and so are the references. The number of effects added should be addressed in Introduction and so the relevant references can also be added.

**Response:**

 Thank you for this important observation. The Introduction has been substantially expanded to clearly discuss the physical significance of the multiple effects considered in the study. Relevant background information has been added, and the discussion has been strengthened by incorporating additional recent and closely related references.

**Comment 5:**

 Reference list is too short, some recent selections on the topic can be included.

**Response:**

 We agree with the reviewer. The reference list has been expanded by including several recent and relevant studies in the field. In particular, the following works have been added and appropriately cited in the revised manuscript to enhance the literature coverage and contextual relevance of the study.

# Reviewer report: Reviewer response for version 1

**DOI:** 10.5256/f1000research.190322.r448712

**Published:** 2026-01-20

**Authors:** Ahmed Gamal

**Affiliations:** 1 Menoufia University, Shebin El-Kom, Egypt

**Summary of the Article**

 The manuscript investigates peristaltic transport of a non-Newtonian fluid under various physical effects using analytical approximations. While the topic itself falls within an active research area, the present study does not demonstrate sufficient originality, methodological rigor, or clarity to meet the standards of theF1000Research.

**Detailed Evaluation**

  Presentation and Literature Coverage

 The manuscript is not clearly presented and does not adequately cite or engage with the current literature. The introduction is brief and largely descriptive, failing to critically analyze prior studies or identify genuine research gaps. Several cited works are outdated, and recent developments in the field are insufficiently addressed.

**Study Design and Technical Soundness**

 The overall study design raises concerns regarding technical soundness. The governing equations—particularly those associated with the selected fluid model—are introduced without appropriate references, making it difficult to verify their correctness or originality.

**Methods, Analysis, and Reproducibility**

 Insufficient methodological details are provided to allow independent replication.

 Key mathematical steps, especially in Section 4.3, are omitted or inadequately explained. Moreover, multiple approximations are employed without defining error bounds or ranges of validity, which compromises the transparency and reliability of the analysis.

**Validation and Reliability**

 The manuscript lacks any form of **validation**. The results are not benchmarked against known analytical solutions, limiting cases, or previously published studies. As a result, the accuracy of the reported findings cannot be confidently assessed.

**Interpretation of Results and Conclusions**

 The results are primarily described in terms of parameter trends, with little to no physical interpretation. Consequently, the conclusions are not sufficiently supported by the results and do not provide meaningful scientific insight.

**Source Data and Parameter Justification**

 The numerical values used in the analysis are not justified or referenced, and no explicit analytical expressions or datasets are provided to reproduce the figures. This limits the reproducibility of the work.

 R **equired Actions to Achieve Scientific Soundness**

 To make the article scientifically sound, the authors would need to address all of the following major issues:

 1. Clearly demonstrate genuine novelty relative to existing literature.

 2. Substantially expand and update the introduction with a critical review of recent studies.

 3. Provide proper references for all governing equations and constitutive relations.

 4. Include validation through limiting cases or comparison with published results.

 5. Justify all mathematical approximations and specify their ranges of validity.

 6. Add clear physical interpretations of the results.

 7. Justify and reference all parameter values used.

 8. Improve the clarity, structure, and English language quality of the manuscript.

 9. Provide sufficient mathematical details to ensure transparency and reproducibility.

Is the work clearly and accurately presented and does it cite the current literature?

No

If applicable, is the statistical analysis and its interpretation appropriate?

Not applicable

Are all the source data underlying the results available to ensure full reproducibility?

No

Is the study design appropriate and is the work technically sound?

Partly

Are the conclusions drawn adequately supported by the results?

No

Are sufficient details of methods and analysis provided to allow replication by others?

Partly

Reviewer Expertise:

Mechanical Engineering and Mathematical Engineering– with specific focus on :Peristaltic flow of complex/non-Newtonian fluids (e.g., Carreau and couple-stress fluids, )Heat and mass transfer in multi-physics systemsMagnetohydrodynamics (MHD) and porous media flowsNanofluids and hybrid nanofluidsAnalytical and numerical modeling techniques (e.g., FEM, perturbation methods)

I confirm that I have read this submission and believe that I have an appropriate level of expertise to state that I do not consider it to be of an acceptable scientific standard, for reasons outlined above.

# Response

**Published:** 2026-02-04

**Authors:** Salwa Al-Tamimi

**Affiliations:** Not Applicable, Al Quadisiya, Iraq

We sincerely thank the reviewer for the constructive and valuable comments, which have greatly contributed to improving the scientific quality and clarity of the manuscript. All raised issues have been carefully addressed, and the manuscript has been revised accordingly. Detailed responses are provided below:

**Comment 1:**

 Clearly demonstrate genuine novelty relative to existing literature.

**Response:**

 We thank the reviewer for this important comment. The manuscript has been revised to clearly highlight the novel aspects of the study, including the unique mathematical modeling approach, the physical effects considered, and the analysis performed, in comparison with recent and relevant literature

**Comment 2:**

 Substantially expand and update the introduction with a critical review of recent studies.

**Response:**

 The Introduction has been significantly expanded to include a critical review of recent studies related to the topic. Key strengths and limitations of prior works are discussed, and the positioning of the current study within the existing literature is clearly outlined

**Comment 3:**

 Provide proper references for all governing equations and constitutive relations.

**Response:**

 All governing equations and constitutive relations have been carefully reviewed, and appropriate references have been added for each to ensure proper scientific documentation and to strengthen the credibility of the model

**Comment 4:**

 Include validation through limiting cases or comparison with published results.

**Response:**

 A validation section has been added, in which selected limiting cases are analyzed and the results are compared with published studies. The comparisons show good agreement, confirming the accuracy and reliability of the model.

**Comment 5:**

 Justify all mathematical approximations and specify their ranges of validity.

**Response:**

 All mathematical approximations used in the study have been justified physically and mathematically, and their applicable ranges have been clearly specified in the revised manuscript

**Comment 6:**

 Add clear physical interpretations of the results.

**Response:**

 The Results and Discussion section has been enhanced with detailed physical interpretations of the numerical findings, explaining how different parameters influence the behavior of the system and providing deeper scientific insight.

**Comment 7:**

 Justify and reference all parameter values used.

**Response:**

 All parameter values used in the numerical analysis have been reviewed, justified based on physical considerations, and supported with references to relevant literature.

**Comment 8:**

 Improve the clarity, structure, and English language quality of the manuscript.

**Response:**

 The manuscript has undergone a comprehensive revision to improve organization, structure, and readability. Language and scientific writing have been enhanced to meet the standards of high-quality publications

**Comment 9:**

 Provide sufficient mathematical details to ensure transparency and reproducibility.

**Response:**

 Additional mathematical details, including derivation steps and numerical procedures, have been provided to ensure full transparency and allow reproducibility of the results by other researchers
